# Secreted LRPAP1 binds and triggers IFNAR1 degradation to facilitate virus evasion from cellular innate immunity

**DOI:** 10.1038/s41392-023-01630-1

**Published:** 2023-09-25

**Authors:** Huangcan Li, Xiong Wang, Yiran Wang, Yichen Li, Ying Chen, Yin-Ting Wong, Jufang He, Ming-Liang He

**Affiliations:** 1 Department of Biomedical Sciences, City University of Hong Kong, Hong Kong, China; 2 CityU Shenzhen Research Institute, Nanshan, Shenzhen, China; 3 Department of Neurosciences, City University of Hong Kong, Hong Kong, China

**Keywords:** Infectious diseases, Microbiology, Infectious diseases

## Abstract

The crucial role of interferon (IFN) signaling is well known in the restriction or eradication of pathogen invasion. Viruses take a variety of ways to antagonize host defense through eliminating IFN-signaling intracellularly for decades. However, the way by viruses target IFN-signaling extracellularly has not been discovered. Infection by both coronavirus SARS-CoV-2 and enterovirus 71 (EV71 or EV-A71) can cause severe diseases such as neurological disorders and even death in children.^1–3^ Here, we show evidence that the protease of SARS-CoV-2 (3CL^pro^) and EV71 (2A^pro^) upregulates the expression and secretion of LDL**-**receptor-related protein-associated protein 1 (LRPAP1). As a ligand, the N-terminus of secreted LRPAP1 binds with the extracellular domain of IFNAR1 that triggers the receptor ubiquitination and degradation and promotes virus infection both in vitro, ex vivo in the mouse brain, and in vivo in newborn mice. A small peptide from the N-terminus of LRPAP1 effectively binds and causes IFNAR1 degradation that enhances both DNA and RNA viral infections, including herpesvirus HSV-1, hepatitis B virus (HBV), EV71, and beta-coronavirus HCoV-OC43; whereas α2M, a LRPAP1 inhibitor, arrests virus infections by stabilizing IFNAR1. Our study demonstrates a new mechanism used by viruses for evading host cell immunity, supporting a strategy for developing pan-antiviral drugs.

## Introduction

The host innate immunity serves as the front line for preventing viral invasion and replication.^4^ The host cells can quickly recognize viral components through pattern recognition receptors (PRRs), such as Toll-like receptors (TLRs), retinoic acid-inducible gene I like receptors (RLRs), etc.^5–7^ Once a pathogen or its critical components is detected, it immediately triggers the expression and secretion of type I interferon (IFNα, IFNβ) and other cytokines in infected cells. Then the secreted interferons (IFNs) bind with and activate the heterodimeric transmembrane IFNα receptor (IFNAR) by autophosphorylation^8^; followed by phosphorylation of JAK and TYK kinases, and the signal transducer and activator of transcription proteins (STAT1/STAT2) to activate the transcription of hundreds of interferon-stimulated genes (ISGs) against virus infection. Such interferon signaling cascades constitute the main body of host’s innate immune response.

To establish successful infection, viruses use a variety of ways to attenuate host cell innate immunity by targeting intracellular interferon signaling.^9^ This process is extremely important for viruses even belonging to different families (e.g., enterovirus EV71, flavivirus Zika virus, coronavirus SARS-CoV-2, and herpes virus HSV-1) to infect nerve cells in the brain because the immune response of the central nervous system (CNS) is mostly based on the local cellular innate immunity.^10,11^ Although positive-sense (+) single-stranded viruses (e.g., EV71, ZIKV, and SARS-CoV-2) complete their life cycle in the cytosol, and DNA viruses (e.g., HSV-1) replicate their genome in the nucleus, they infect neural cells in the CNS and cause acute or chronic neurological disorders (e.g., paralysis, neurodegenerative diseases) and even deaths. After entry and uncoating, the genome RNA of (+) ssRNA viruses is translated into a polypeptide, which is further cleaved by virus-encoded protease into functional proteins. Viral proteases share important roles in counteracting innate immune responses. Enterovirus 2A^pro^ attenuates type I interferon through targeting MDA5, a retinoid acid-inducible gene I (RIG-I) like receptor.^12^ The SARS-CoV-2 main protease (M^pro^, also known as 3CL^pro^) is also reported to restrict the ubiquitination of RIG-I and promote the degradation of STAT1,^13^ indicating a possible shared mechanism in multiple virus proteases.

Direct targeting IFNAR1 by viruses is an effective strategy to attenuate the innate immune response. Although IFNAR1 degradation can be triggered by inside signals or other receptors,^14,15^ it cannot explain how viral proteins dramatically decrease cellular IFNAR1. We previously reported that 2A protease (2A^pro^) of EV71 is the main contributor to decreasing cellular IFNAR1.^16^ However, 2A^pro^ cannot directly digest IFNAR1, suggesting the involvement of other host proteins in the process. Here, we report that the low-density lipoprotein receptor related protein associated protein 1 (LRPAP1) interacts with 2A^pro^. It is reported that dysfunction or deficiency of LRPAP1 is associated with neurodegenerative diseases, including degenerative dementias (e.g., Alzheimer’s disease, Atherosclerosis, etc.) and myopia.^17,18^ Accumulating evidence indicates that these neurodegenerative diseases may be caused by attenuated innate immunity in response to chronic infections in the brain. Interestingly, EV71 infection and EV71-encoded 2A^pro^ promoted the secretion of LRPAP1 into the extracellular environment, leading to a more than 5-fold decrease in the expression level of IFNAR1 protein on the cell surface. More importantly, results from our study demonstrated a pan-antiviral strategy through targeting LRPAP1.

## Results

### EV71 increases LRPAP1 expression to promote virus infection

Because 2A^pro^ cannot directly cleave IFNAR1,^16^ we postulated that other host proteins participate in this important process. To identify proteins that interact with EV71 2A^pro^, we used recombinant 2A^pro^ (r2A) and protease-inactive 2A^C110A^ mutant (2Am) as baits for mass-spectrum (MS) and microscale-thermophoresis (MST) analysis. Interestingly, we revealed that LRPAP1, a chaperone-like protein, interacts with 2A^pro^ (Supplementary Fig. S1a–c and Supplementary Table S1). LRPAP1 can transport low-density lipoprotein receptor related proteins (LRPs) from the endoplasmic reticulum (ER) to the Golgi complex and functions as a potent antagonist of LRPs on cell surfaces.^19^

**Fig. 1:**
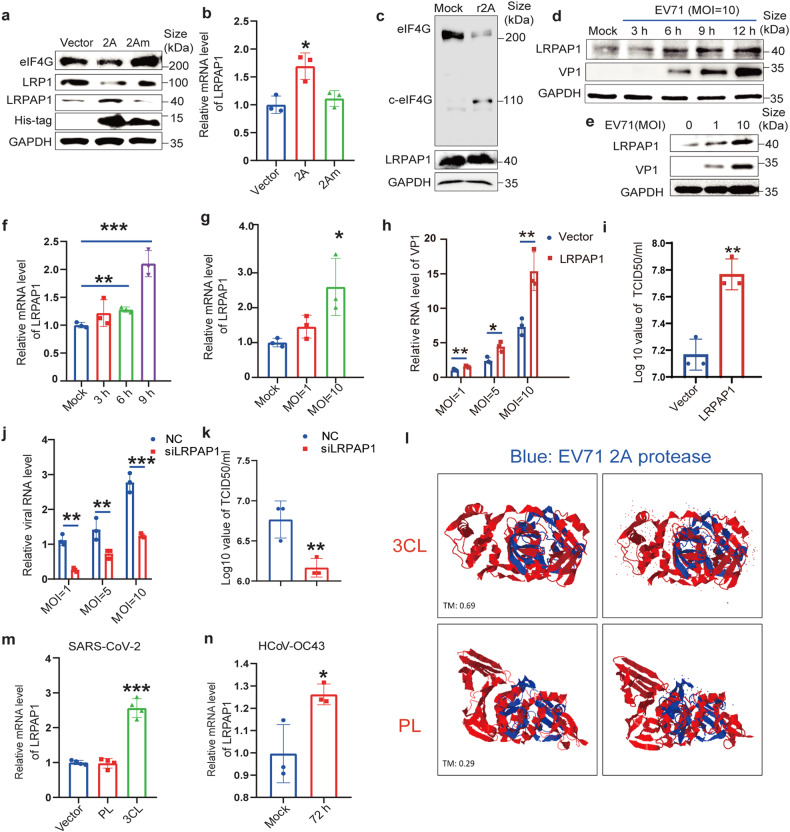
Both EV71 and coronavirus infection promote LRPAP1 expression. **a**, **b** Immunoblots and RT-qPCR analysis of the protein (**a**) and mRNA levels (**b**) of LRPAP1 in HEK-293T cells transfected with pcDNA4B (vector), pcDNA4B-2A or pcDNA4B-2Am, respectively. **c** Immunoblots of lysates in RD cells incubated with or without recombinant 2A^pro^ (r2A) for 1 h. **d**–**g** RD cells were infected with EV71 at the indicated MOI, and cell lysates were obtained at different time points and subjected to immunoblots (**d**, **e**) and RT-qPCR analysis (**f**, **g**). **h**–**k** The relative viral RNA level (**h**, **j**) and viral titer (**i**, **k**) in RD cells infected with EV71 at the indicated MOI for 9 h. RD cells were transfected with the indicated plasmid (empty vector (Vector) or pcDNA-LRPAP1 (LRPAP1)) or siRNA (scramble RNA (NC) or siRNA-LRPAP1 (siLRPAP1)) for 39 h before virus infection. **l** The merging superposition of indicated proteins (EV71 2 A, 4fvb; SARS-CoV-2 3CL, 6y2e; SARS-CoV-2 PL, 6w9c) were compared in 3D model (https://zhanglab.ccmb.med.umich.edu/TM-align/). The right panel indicated the superposition of the interested proteins (3CL (red)/2 A (blue) or PL (red)/2 A (blue)) with ligands and solvents. The TM-score above 0.5 means structural similarity. **m** The relative mRNA level of LRPAP1 in HEK-293T cells transfected with empty vector pCMV, pCMV-SARS-CoV-2-PL, or pCMV-SARS-CoV-2-3CL for 48 h. **n** The relative mRNA level of LRPAP1 in RD cells infected with HCoV-OC43 at an MOI of 1 for 72 h. Results were expressed as mean ± standard deviation (error bars) of at least three repeats. *P ≤ 0.05, **P ≤ 0.01, ***P ≤ 0.001, ****P ≤ 0.0001 (unpaired *t*-test)

Although transcriptomic and proteomic data showed that many viruses (e.g., HCV, LCMV, EBV, KSHV, and SARS-CoV-2) upregulate LRPAP1,^20,21^ its function in infections has not yet been characterized. Consistent with others’ findings,^19,22,23^ the increased expression of LRPAP1 induced by 2A^pro^ decreased LRP1 protein level (Fig. 1a and Supplementary Fig. S1d, e). Interestingly, 2A^pro^ cleaved eIF4G effectively but cannot directly cleave LRPAP1 (Fig. 1a–c and Supplementary Fig. S1f). 2A^pro^ (but not 2Am, Fig. 1a) as well as EV71 (Fig. 1d–g and Supplementary Fig. S1g–k) increased LRPAP1 expression at both mRNA and protein levels in RD and HEK-293T cells, whereas other viral proteins, such as 3C^pro^ and 3D (RdRp), showed no effect (Supplementary Fig. S1l, m). Moreover, we discovered that the ectopically expressed LRPAP1 strikingly promoted EV71 infection (Fig. 1h, i), whereas LRPAP1 knockdown markedly suppressed EV71 replication and propagation by over 80% and a similar result was confirmed by other two siRNA (Fig. 1j, k and Supplementary Fig. S1n–p).

To reveal if proteases encoded by different viruses have similar functions, we aligned EV71 2A^pro^ sequences with other viral proteases. Amazingly, a high sequence conservation was discovered among proteases in picornaviruses, including both rhinovirus (RV-C) and enterovirus (Coxsackievirus A16). Moreover, a high similarity was also found among EV71 2A^pro^ and coronavirus 3CL^pro^ (MERS-CoV and SARS-CoV-2) and flavivirus protease (ZIKA virus and Dengue virus) (Fig. S1q). These indicated a potentially common effect of multiple viral proteases on LRPAP1.

Similar to EV71, SARS-CoV-2 can also cause strong neurological disorders and even death.^24^ Previous studies have implicated that the similarities between coronaviral protease and enterovirus 2A^pro^ serve to suppress innate immunity.^13,25–27^ Except for comparable amino acid sequence, we also detected a similar 3D structure between enterovirus 2A^pro^ and SARS-CoV-2 3CL^pro^ (Fig. 1l). Therefore, we used coronavirus 3CL^pro^ as a representative to further explore the effect of multiple proteases on LRPAP1 and viral infection. Interestingly, SARS-CoV-2 3CL^pro^ also increased LRPAP1 expression, while PL^pro^ had no effect (Fig. 1m). Similar to EV71 2A^pro^, a decreased protein level, but not RNA level, of LRP1 was detected with SARS-CoV-2 3CL^pro^ transfection (Supplementary Fig. S1r, s). Moreover, 3CL^pro^ is an evolutionary conserved protein in different coronaviruses. We showed a high identity of amino acid sequence between 3CL^pro^ in SARS-CoV-2 and HCoV-OC43 (Supplementary Fig, S1t). An increased LRPAP1 expression was further detected by HCoV-OC43 infection, indicating the similar effects of coronavirus on promoting LRPAP1 expression (Fig. 1n).

### Secreted LRPAP1 assists both coronavirus and EV71 infection

**Fig. 2:**
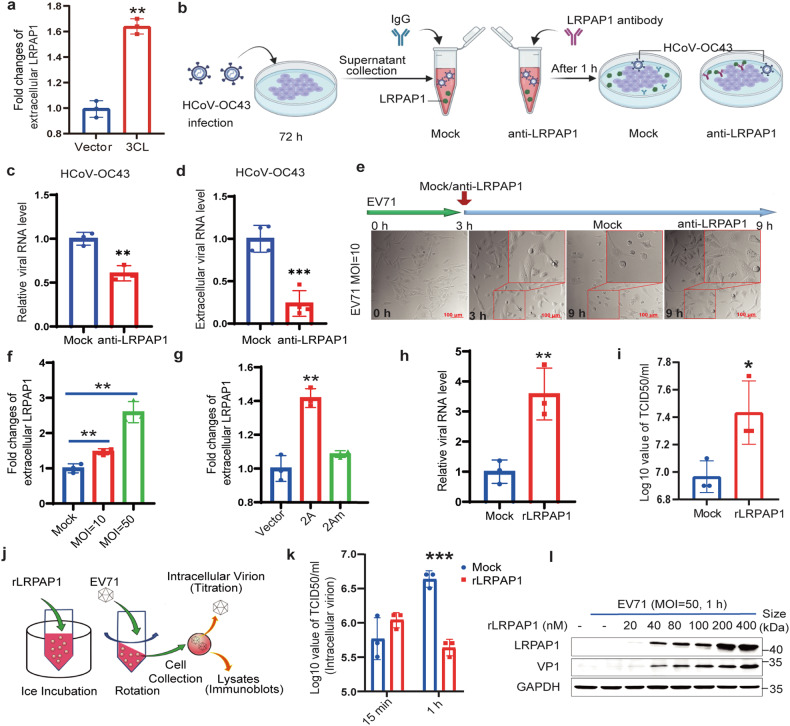
Extracellular LRPAP1 promotes both coronavirus and EV71 infection. **a** HEK-293T cells were transfected with an empty vector (Vector) or pCMV-Flag-3CL (3CL). The fold changes of extracellular LRPAP1 protein level were then measured from the medium via ELISA. **b**–**d** The schematic diagram of the effect on viral infection caused by extracellular LRPAP1 induced by HCoV-OC43 infection. Created with BioRender.com (**b**). RD cells were infected with HCoV-OC43 at an MOI of 2 for 72 h. The supernatant was collected and treated with either 0.5 μg/ml antibody against LRPAP1 (anti-LRPAP1) or IgG for 1 h. The supernatant was then used to infect new RD cells for 12 h. The intracellular (**c**) and extracellular (**d**) viral RNA level was measured. **e** RD cells were infected with EV71 at an MOI of 10 for 3 h. Then cells were treated with or without the antibody against LRPAP1 (anti-LRPAP1) for 6 h. The CPEs were indicated by rounding and detachment of cells (scale bar = 100 μm). **f**, **g** The fold changes of extracellular LRPAP1 protein level in RD cells infected with EV71 at the indicated viral load for 9 h (**f**) or HEK-293T cells transfected with the indicated plasmids for 48 h (**g**) were measured by ELISA. **h**, **i** The relative viral RNA level (**h**) and viral titer (**i**) in RD cells infected with EV71 after the treatment with or without recombinant LRPAP1. **j–l** The schematic diagram of recombinant LRPAP1 (rLRPAP1) induction for a short-term virus infection (**j**). HEK-293T cells were pre-treated with rLRPAP1 on ice for 1 h. Then cells were inoculated with EV71 at an MOI of 50 for 15 min or 1 h. Intracellular virions were harvested and subjected to viral titration (**k**). Lysates were subjected to immunoblotting with anti-VP1 and anti-LRPAP1 antibodies (**l**). Results were expressed as mean ± standard deviation (error bars) of at least three repeats. *P ≤ 0.05, **P ≤ 0.01, ***P ≤ 0.001, ****P ≤ 0.0001 (unpaired *t*-test)

LRPAP1 is secreted into the extracellular environment when excessive amounts are produced from the intracellular LRPs transportation system.^22^ We further confirmed an extracellular LRPAP1 increase in SARS-CoV-2 3CL^pro^ expressing cells (Fig. 2a). Moreover, the extracellular LRPAP1 was also collected after HCoV-OC43 infection (Fig. 2b). The intracellular and extracellular viral RNA level decreased about 40 and 70% after treated with LRPAP1 antibody (Fig. 2c, d), respectively. LRPAP1 antibody treatment also significantly protected cells from cytopathic effects (CPE) caused by virus infection (Supplementary Fig. S2a), indicating the extracellular LRPAP1 induced by HCoV-OC43 promoted viral infection.

Additionally, LRPAP1 antibody significantly decreased EV71 induced CPEs when it was added into the culture medium 3 h post infection (Fig. 2e). An increased extracellular LRPAP1 was also detected in both EV71-infected and 2A^pro^-expressing cells but not 2A^C110A^ expressing cells by ELISA assay (Fig. 2f, g). More importantly, the rLRPAP1 supplemented in the culture medium significantly promoted EV71 infection and EV71-induced CPE (Fig. 2h, i and Supplementary Fig. S2b). A short-term extracellular induction with rLRPAP1 promoted EV71 entry and uncoating rate within 1 h in HEK-293T cells (Fig. 2j–l and Supplementary Fig. S2c). Next, we further investigated the effect of rLRPAP1 in the EV71-infected C57BL/6 mice model (Supplementary Fig. S2d). Surprisingly, rLRPAP1 potently enhanced EV71 infection and exacerbated the disease in newborn C57BL/6 mice (Supplementary Fig. S2e, f). Moreover, C57BL/6 mouse co-injected with EV71 and rLRPAP1 showed severe physical disharmony after post-injection (Supplementary Fig. S2g, h and Video S1, 2). Taken together, these results suggested a crucial role for the secreted LRPAP1 in facilitating virus infection both in vitro and in vivo.

### Extracellular LRPAP1 dominates the downregulation of IFNAR1

**Fig. 3:**
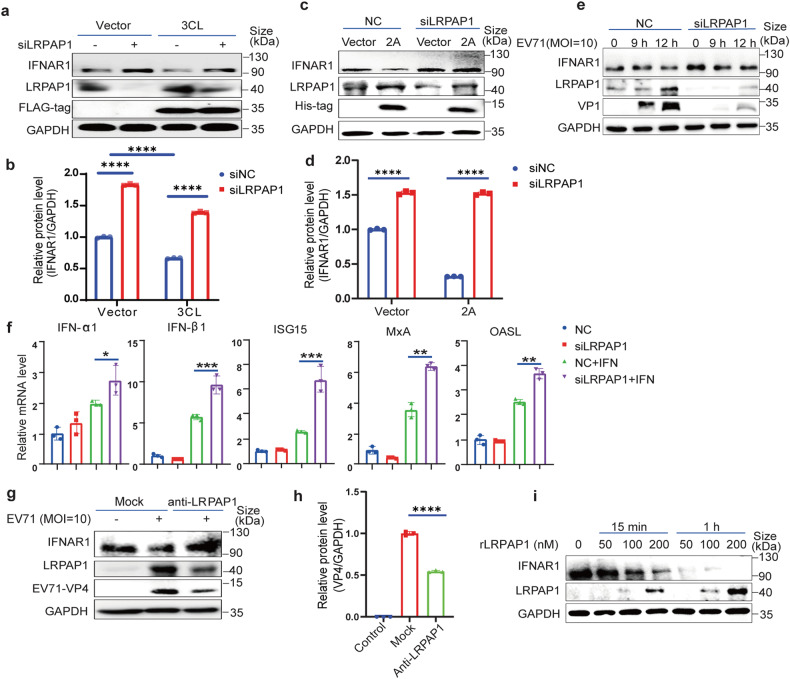
Extracellular LRPAP1 dominates the downregulation of IFNAR1. **a** Immunoblots of lysates in HEK-293T cells with the indicated gene expression. HEK-293T cells were transfected with scramble siRNA or siLRPAP1 for 24 h, and cells were then transfected with empty vector or CMV-flag-3CL for another 24 h. **b** Densitometric analysis of IFNAR1 levels in Fig. 3a was quantified and normalized with GAPDH using ImageJ. **c**–**e** Immunoblots of IFNAR1 in the cell lysates with LRPAP1-knockdown. HEK-293T cells were initially transfected with scramble siRNA (NC) or siLRPAP1 for 24 h. Then cells were transfected with an empty vector or 2A-expressing plasmid for a further 24 h (**c**), or cells were infected with the EV71 for the indicated time (**e**). Densitometric analysis of IFNAR1 levels in Fig. 3c was quantified and normalized with GAPDH using ImageJ (**d**). **f** RD cells were transfected with scramble siRNA or siLRPAP1 for 48 h and were treated with or without 1000 U/ml IFN-α2b for 1 h. The down-stream response of the type I IFN signaling pathway was evaluated by the relative mRNA level of the indicated ISGs. **g**, RD cells were infected with EV71 at an MOI of 10 for 3 h. Then cells were treated with or without the antibody against LRPAP1 (anti-LRPAP1) for 6 h. Lysates were subjected to immunoblotting with anti-IFNAR1, anti-EV71 VP4 and anti-LRPAP1. **h** Densitometric analysis of VP4 levels in Fig. 3g was quantified and normalized with GAPDH using ImageJ. **i** Immunoblots of lysates in HEK-293T cells which were treated with the indicated concentration of rLRPAP1 for 15 min or 1 h. Results were expressed as mean ± standard deviation (error bars) of at least three repeats. *P ≤ 0.05, **P ≤ 0.01, ***P ≤ 0.001, ****P ≤ 0.0001 (unpaired *t*-test)

Since the LRPAP1 antibody significantly inhibited coronavirus activities, we hypothesized that the secreted LRPAP1 served as an inhibitory ligand for IFNAR1. We found out that 3CL^pro^ increased LRPAP1 expression and decreased IFNAR1 level in wild type HEK-293T cells, compared by lane 1 and lane 3 (Fig. 3a, b). However, the expression of IFNAR1 increased in LRPAP1 knockdown cells (Fig. 3a, lane 1 and lane 2). Interestingly, the decreased IFNAR1 level induced by 3CL^pro^ transfection was rescued by LRPAP1 silencing, which agreed with our hypothesis.

Moreover, we compared the IFNAR1 and LRPAP1 levels that were ectopically expressed by 2A^pro^ alone and by both LRPAP1 and 2A^pro^ simultaneously. EV71 and 2A^pro^ stimulated LRPAP1 expression and reduced the IFNAR1 level, with LRPAP1 alone decreasing IFNAR1 to a marginal detectable level in HEK-293T cells (Fig. S3a–f). Further, the expression of LRPAP1 synergized with the 2A^pro^ impact, leading to a complete undetectability of IFNAR1 levels (Fig. S3c, d). On the contrary, LRPAP1 knockdown mitigated 2A^pro^-induced IFNAR1 downregulation (Fig. 3c–e and Fig. S3g). Excessive LRPAP1 expression promoted EV71 infection by suppressing the type I IFN signaling pathway and the expression of downstream interferon-stimulated genes (ISGs, Fig. S3h), whereas LRPAP1 knockdown enhanced ISGs’ expression (Fig. 3f). Taken together, these results indicated a critical role for LRPAP1 in downregulating IFNAR1 and IFN-signaling by 3CL^pro^ of SARS-CoV-2 and 2A^pro^ of enterovirus 71.

The effect of secreted LRPAP1 on IFNAR1 decrease was also reassessed via LRPAP1-antibody treatment. Consistent with CPE assay results in Fig. 2e, the IFNAR1 level was successfully restored to normal levels with LRPAP1-antibody treatment post EV71 infection. Surprisingly, LRPAP1-antibody also decreased the levels of intracellular LRPAP1 and virus protein VP4 (Fig. 3g, h). To further illustrate extracellular LRPAP1’s dominant role in downregulating IFNAR1, HEK-293T cells were incubated with the indicated concentrations of rLRPAP1 for up to 15 min or 1 h. IFNAR1 rapidly decreased within 15 min in a rLRPAP1 dose-dependent manner. Within just an hour of treatment with rLRPAP1, IFNAR1 level decreased to a marginal detectable level at a concentration of 50 nM and even to an undetectable level at 200 nM (Fig. 3i and S3i). These further illustrated that the extracellular LRPAP1 downregulated IFNAR1.

### LRPAP1 triggers IFNAR1 degradation by binding to the latter’s extracellular domain in vitro and ex vivo in the brain

**Fig. 4:**
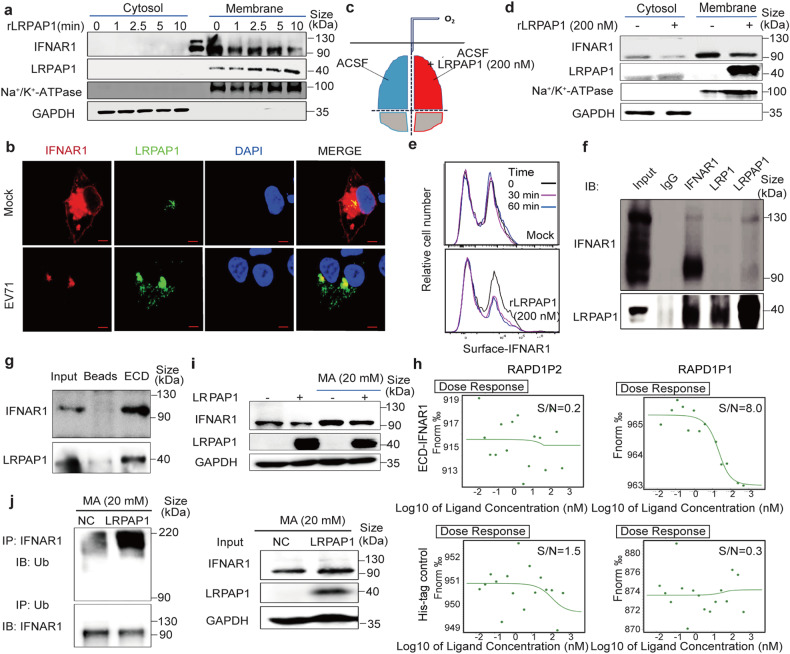
LRPAP1 binds with IFNAR1 and triggers IFNAR1 degradation. **a** Immunoblots of cytosol and membrane fraction in HEK-293T cells treated with 200 nM rLRPAP1 from 1 min to 10 min. **b** Immunofluorescence of RD cells infected with or without EV71 (MOI = 10) for 6 h. Cells were double stained with anti-IFNAR1 (Red) and anti-LRPAP1 (Green) (scale bar = 5 μm). **c**, **d** The C57BL/6 mouse cerebrum was separated into two hemispheres and immediately cultured in oxygen supplied ACSF with or without rLRPAP1 (200 nM) at 0 °C for 1 h (**c**). The cytosol and membrane fraction from brain tissue was then analysed by immunoblotting (**d**). **e** The flow cytometry analysis of cell surface IFNAR1 protein level on 4T1 cells after the treatment with or without rLRPAP1. **f** Immunoprecipitates (indicated antibodies) in HEK-293T cells ectopic-expressing LRPAP1 and IFNAR1 were analysed by immunoblotting. **g** The pull-down assay between the extracellular domain of IFNAR1 (ECD, Sino biological 13222-H08H) and lysates in HEK-293T cells overexpressing LRPAP1 were analysed by immunoblotting. **h** Microscale thermophoresis (MST) results for the binding affinity between the region of LRPAP1 (RAPD1P1, RAPD1P2) and ECD-IFNAR1. **i** Immunoblots of LRPAP1 ectopic-expressing HEK-293T cells treated with or without 20 mM MA for 4 h. **j** Immunoprecipitates (indicated antibodies) in HEK-293T cells ectopic-expressing ubiquitin, LRPAP1 and IFNAR1 were analysed by immunoblotting. Cells were treated with 20 mM MA for 4 h before harvested

To address how extracellular LRPAP1 interfered with IFNAR1 expression at the very early stages of virus infection, protein levels on the membrane and in the cytosol fractions were compared between cells without treatment and treated with 200 nM rLRPAP1 for 1 min to 10 min. We showed that IFNAR1 consistently decreased with time while rLRPAP1 became gradually enriched in the membrane fractions (Fig. 4a and S4a). Moreover, IFNAR1 colocalized well with LRPAP1 and sharply decreased in the cell membrane, which was accomplished by a dramatic increase in LRPAP1 after EV71 infection (Fig. 4b) or by rLRPAP1 treatment (Fig. S4b and Video S3, 4).

In the brain, the neuron cell’s innate immunity plays the main role in limiting or eradicating virus infection because nerve cells are host cells of viruses and the adaptive immunity is low there. Infections by many viruses (SARS-CoV-2, ZIKV, DENV, EV71, etc.) can cause severe neurological disorders and even death, such as aseptic meningitis, encephalitis, poliomyelitis-like paralysis, and mental disorders.^28^ To examine if LRPAP1 promoted virus infection in nerve cells in the brain, the effect of extracellular rLRPAP1 on membrane IFNAR1 was also confirmed ex vivo using mouse brains. The freshly prepared cerebrum from 8-week-old C57BL/6 mouse was incubated in oxygen supplied artificial cerebrospinal fluid (ACSF), with or without rLRPAP1 (200 nM) at 0 °C for 1 h. Compared to the control hemisphere of the same mouse, IFNAR1 levels were significantly lower in the rLRPAP1-treated hemisphere (Fig. 4c, d, and Supplementary Fig. S4c). To better delineate the interaction of rLRPAP1 and IFNAR1 on the cell surface, the APC-labelled extracellular domain of IFNAR1 was determined by flow cytometry in live 4T1 cells treated with and without rLRPAP1. Again, the extracellular rLRPAP1 significantly reduced cell surface IFNAR1 (Fig. 4e). We also noted that LRPAP1 had no effect on IFNAR1 mRNA levels with or without interferon treatment (Supplementary Fig. S4d, e). Taken together, the extracellular rLRPAP1 directly downregulated IFNAR1 on the cell membrane without affecting the transcription and mRNA stability of IFNAR1.

We further confirmed the interaction of LRPAP1 and IFNAR1 by co-immunoprecipitation assay (Fig. 4f). Their binding was also observed in the mouse cerebrum (Supplementary Fig. S4f). Furthermore, a pull-down assay revealed that rLRPAP1 directly bound with the extracellular domain of IFNAR1 (Residues 1–436, Fig. 4g). Having the potential binding region predicted through molecular docking (Supplementary Fig. S4g and Table S2, 3), we selected and synthesized peptides from N-terminal of LRPAP1, RAPD1P1 (residues 35–53) and RAPD1P2 (residues 55–75), the former with binding potential while the latter without. We showed that RAPD1P1 significantly decreased the level of both IFNAR1 and its downstream antiviral ISGs (Fig. S4h, i). Like rLRPAP1, RAPD1P1 also significantly promoted EV71 infection (Fig. S4j, k). Importantly, RAPD1P1, but not RAPD1P2, bound with the extracellular domain of IFNAR1 (Kd = 11.3 nM), as observed via microscale thermophoresis (MST) assay (Fig. 4h).

IFNAR1 degradation starts with its phosphorylation and is followed by endocytosis.^29,30^ Afterward, the phosphorylated IFNAR1 (p-IFNAR1) undergoes ubiquitination and is subjected to the lysosome for degradation.^31^ We first revealed that the binding between rLRPAP1 and IFNAR1 induced IFNAR1 endocytosis by detecting an increased level of early endosome marker (EEA1) (Supplementary Fig. S4l). Additionally, phosphorylated IFNAR1 is found to be promoted in an extracellular rLRPAP1 dose-dependent manner (Supplementary Fig. S4m, n). IFNAR1 ubiquitination was also upregulated by LRPAP1, indicating that LRPAP1 induced IFNAR1 degradation (Supplementary Fig. S4o). Further, the reduction of IFNAR1 as induced by LRPAP1 was prohibited by lysosome inhibitors, including methylamine (MA, Fig. 4i) and bafilomycin (Supplementary Fig. S4q), but not by proteasomal inhibitors MG132 or leupeptin (Supplementary Fig. S4p). As LRPAP1 dramatically increased IFNAR1 ubiquitination, and IFNAR1 was protected from degradation after MA treatment (Fig. 4j), collectively demonstrating that LRPAP1 served as an IFNAR1 ligand that induced lysosome-dependent IFNAR1 degradation.

### The N-terminal of LRPAP1 promotes infection by multiple (both DNA and RNA) viruses

**Fig. 5:**
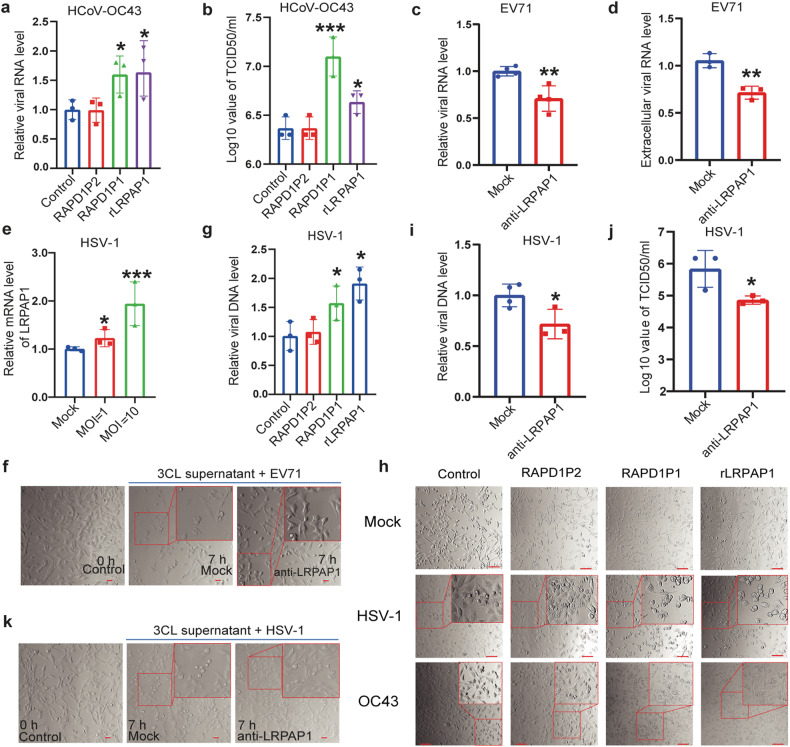
Extracellular LRPAP1 promotes multiple viruses’ infection. **a**, **b** The effect of rLRPAP1 and RAPD1P1 on HCoV-OC43 infection. RD cells were pre-treated with the scramble peptide (control), RAPD1P1, RAPD1P2, or rLRPAP1 for 1 h. Cells were then infected with HCoV-OC43 at an MOI of 1 for 72 h. HCoV-OC43 RNA levels were determined by RT-qPCR (**a**). Viral titer of HCoV-OC43 was measured by TCID_50_ assay (**b**). **c**, **d**, **f** HEK-293T cells were transfected with pCMV-SARS-CoV-2-3CL for 48 h. Supernatant was collected and incubated with 0.5 μg/ml LRPAP1 antibody or IgG for 1 h. Supernatant was then put into RD cells for 1 h and then infected with EV71 (MOI = 20) for 7 h. The intracellular (**c**) and extracellular (**d**) EV71 RNA was determined by RT-qPCR. The CPE of EV71 was indicated by rounding and detachment of cells (scale bar = 20 μm) (**f**). **e** The relative mRNA level of LRPAP1 in RD cells infected with HSV-1 for 24 h. **g** The effect of rLRPAP1 and RAPD1P1 on HSV-1 infection. RD cells were pre-treated with the scramble peptide (control), RAPD1P1, RAPD1P2, or rLRPAP1 for 1 h. Cells were then infected with HSV-1 at an MOI of 10 for 24 h. HSV-1 DNA levels were determined by RT-qPCR. **h** The effect of rLRPAP1 and LRPAP1 peptides (RAPD1P1, RAPD1P2) on the infection of HSV-1 and HCoV-OC43. Cells were pre-treated with 1 μM of the indicated peptides or 200 nM rLRPAP1 for 1 h, and cells were then inoculated with the indicated virus for the indicated time (HSV-1, 24 h; HCoV-OC43, 72 h). The CPE of HSV-1 was indicated by the rounding and detachment of cells, while the CPE of HCoV-OC43 was measured by the vacuoles in the cytoplasm (scale bar = 100 μm). **i–k** HEK-293T cells were transfected with pCMV-SARS-CoV-2-3CL for 48 h. Supernatant was collected and incubated with either 0.5 μg/ml LRPAP1 antibody or IgG for 1 h. Supernatant was then put into RD cells for 1 h and then infected with HSV-1 (MOI = 20) for 7 h. The intracellular HSV-1 DNA was determined by RT-qPCR (**i**). The viral titer of HSV-1 was measured by TCID_50_ assay (**j**). The CPE of HSV-1 was indicated by rounding and detachment of cells (scale bar = 20 μm) (**k**). Results were expressed as mean ± standard deviation (error bars) of at least three repeats. *P ≤ 0.05, **P ≤ 0.01, ***P ≤ 0.001, ****P ≤ 0.0001 (unpaired *t*-test)

We further confirmed the increased HCoV-OC43 RNA replication and viral propagation by using rLRPAP1 or PAPD1P1. Both the intracellular RNA level and viral titer increased significantly with rLRPAP1 or RAPD1P1, but not with RAPD1P2 (Fig. 5a, b). We also detected more obvious cytopathic effects caused by HCoV-OC43 infection after being treated with LRPAP1 and RAPD1P1 (Fig. 5h). Subsequently, the effect of secreted LRPAP1 induced by coronavirus 3CL^pro^ was tested on other viruses’ infection (Supplementary Fig. S5a). Both the intracellular and extracellular EV71 RNA levels were reduced by 30% and a less obvious CPE was also found with LRPAP1 antibody (Fig. 5c, d, and f). Subsequently, we also confirmed the correlation between 3CL^pro^ and IFNAR1. The expression of IFNAR1 decreased because of the promotion of LRPAP1 induced by 3CL^pro^ (Supplementary Fig. S5b–d).

On the broad inhibition of IFN signaling by multiple viruses,^32–35^ we raised the question of whether the promotion of LRPAP1 expression and secretion was a conserved strategy adopted by viruses to evade the host cell’s innate immunity. Other than RNA viruses (non-enveloped virus EV71, and enveloped coronavirus HCoV-OC43), we also revealed that LRPAP1 expression was promoted by DNA virus herpesvirus HSV-1 (Fig. 5e and Supplementary Fig. S5e, f). A more obvious cytopathic effect caused by HSV-1 infection was found after treated with LRPAP1 and RAPD1P1 (Fig. 5h). More strikingly, we found that LRPAP1, as well as RAPD1P1, promoted the DNA replication of HSV-1 (Fig. 5g). Subsequently, the impact of extracellular LRPAP1 induced by SARS-CoV-2 3CL^pro^ was further illustrated on HSV-1 infection (Supplementary Fig. S5a). The decreased intracellular HSV-1 DNA level and TCID_50_ were observed after adding LRPAP1 antibody, while a less obvious CPE was found with the infection of HSV-1 (Fig. 5i–k). These indicated that the promoted extracellular LRPAP1 caused by viruses to improve viral infection was a conserved strategy, which applied to non-enveloped viruses and enveloped viruses with RNA or DNA genome.

### Inhibiting LRPAP1 suppresses multiple viruses’ infection

**Fig. 6:**
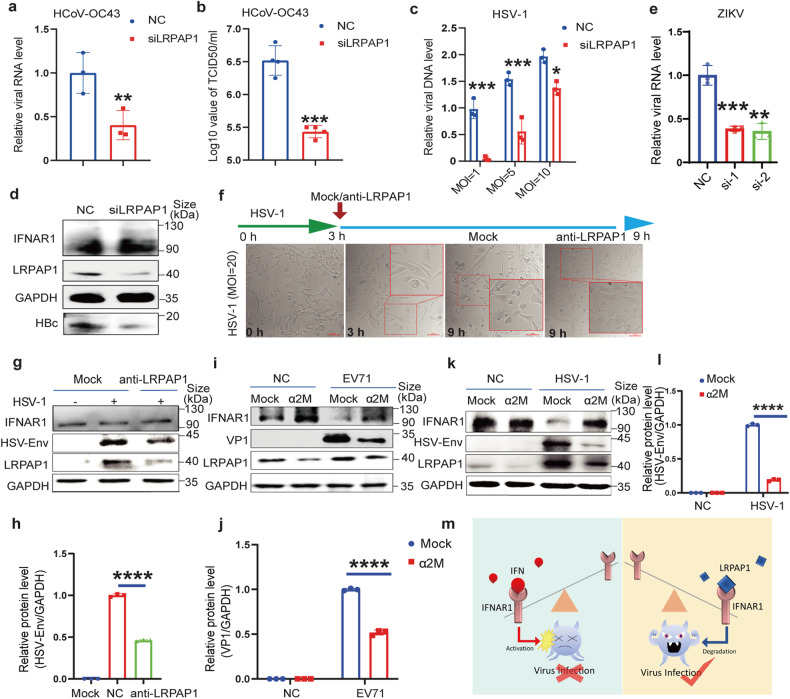
Inhibition of extracellular LRPAP1 reinforces the host cell’s innate immunity against multiple viruses’ infection. **a**–**c** RD cells were transfected with scramble siRNA or siLRPAP1 for 24 h. Then the cells were infected with HCoV-OC43 at an MOI of 1 for 72 h or HSV-1 at the indicated MOI for 24 h. Viral RNA level of HCoV-OC43 was analysed by RT-qPCR (**a**). Viral titer of HCoV-OC43 was measured by TCID_50_ assay (**b**). The relative viral DNA level of HSV-1 was analysed by RT-qPCR (**c**). **d** Immunoblots of lysates in HepAD38 cells transfected with scramble siRNA or siLRPAP1 for 48 h. **e** RD cells were transfected with scramble siRNA or siLRPAP1-1/-2 (si-1, si-2) for 24 h. Then the cells were infected with ZIKV at an MOI of 1 for 24 h. Viral RNA level of ZIKV was analysed by RT-qPCR. **f**, **g** The inhibitory effect of anti-LRPAP1 for HSV-1 infection. RD cells were inoculated with HSV-1 at an MOI of 20 for 3 h. Then cells were treated with or without 0.5 μg/ml LRPAP1 antibody for another 6 h. Cell morphology of RD cells treated with or without anti-LRPAP1 against HSV-1 infection. The CPE of HSV-1 was indicated by rounding and detachment of cells (scale bar = 100 μm). **f** Immunoblots of lysates in RD cells treated with or without antibody of LRPAP1 in the context of HSV-1 infection (**g**). **h** Densitometric analysis of HSV Envelope levels in Fig. 6g was quantified and normalized with GAPDH using ImageJ. **i–l** RD cells were infected with EV71 or HSV-1 at an MOI of 10 for 3 h. Then cells were treated with or without 20 nM α2M for further the indicated time (EV71, 6 h; HSV-1, 21 h). Immunoblots showing the effect of α2M on the infection of EV71 (**i**) or HSV-1 (**k**). Densitometric analysis of VP1 levels in Fig. 6(**i**) was quantified and normalized with GAPDH using ImageJ (**j**). Densitometric analysis of HSV Envelope levels in Fig. 6(**k**) was quantified and normalized with GAPDH using ImageJ (**l**). **m** The schematic diagram of virus circumventing type I IFN signaling through LRPAP1. Results were expressed as mean ± standard deviation (error bars) of at least three repeats. *P ≤ 0.05, **P ≤ 0.01, ***P ≤ 0.001, ****P ≤ 0.0001 (unpaired *t*-test)

Subsequently, we investigated if a broad antiviral effect can be achieved by inhibiting or depleting LRPAP1. First, we observed significantly decreased HCoV-OC43 RNA level and viral titer with LRPAP1 knockdown, while fewer obvious CPEs were also detected (Fig. 6a, b and Supplementary Fig. S6a, b). Similar result was found with HSV-1 infection (Fig. 6c). Moreover, we used a stably HBV expressed cell line, HepAD38,^36^ and detected an upregulated expression of IFNAR1 and a significantly decreased HBV level (a DNA retrovirus) in the LRPAP1 knockdown cells (Fig. 6d and Supplementary Fig. S6c–e). Interestingly, the RNA level of ZIKV (RNA flavivirus) decreased by over 60% with LRPAP1 knockdown (Fig. 6e). The increased expression of IFNAR1 with lower viral loads was also discovered in the knockdown cells after the infection of ZIKV (Supplementary Fig. S6f, g).

Consistently, we observed a decreased level of IFNAR1 with higher HBV loads after LRPAP1 overexpression (Supplementary Fig. S6h, i). We postulated that a pan-antiviral strategy is able to be achieved by targeting LRPAP1. To that end, we found a strong inhibitory effect from LRPAP1 antibody on HSV-1 infection, detected by a less obvious CPE and a decreased viral protein level (Fig. 6f–h). In addition, LRPAP1 was known to compete with other ligands’ binding in the extracellular environment, which includes alpha-2-macroglobulin (α2M) and lactoferrin (LF).^37–40^ Both α2M and LF are secreted proteins that are abundant in mammalian blood, spinal fluid, and other biological fluids. Herein, we verified that α2M, but not LF, markedly upregulated IFNAR1 under viral infections or treated with rLRPAP1 (Fig. 6i, k and Supplementary Fig. 6Sj–o). More importantly, α2M significantly inhibited both DNA and RNA virus propagation (Fig. 6i–l, and Supplementary Fig. S6n–r). Therefore, we concluded that the inhibition of LRPAP1 reduced multiple viral infections that were not limited to RNA viruses (coronavirus, enterovirus, flavivirus), but also applied to DNA viruses (herpes virus and hepadnavirus).

## Discussion

Viral proteases play crucial roles in enhancing viral replication through interacting, modifying, and cleaving host/virus proteins. EV71 2A^pro^ not only cleaves host proteins to enhance viral protein translation, but also interacts with many signaling proteins to regulate viral RNA process and translation.^41,42^ It was found that 2A^pro^ also inhibits the processing body (P body) in host cells and sequester the component protein to promote viral RNA synthesis.^43^ Here, we show that both enterovirus 2A^pro^ and SARS-CoV-2 3CL^pro^ promoted the production and secretion of the host protein LRPAP1, a lipoprotein, to assist viral infection through targeting innate immune response. On the other hand, we discovered that multiple viral proteases share similar sequences, especially those in the same family. Therefore, testing whether other virus-encoded proteases have related functions is a favorable question that can be addressed.

Lipoproteins can bind to pathogens, including bacteria, viruses, and parasitic particles, and prevent the entry of the invader. Viruses (e.g. HCV and HBV) can hijack host lipoprotein to facilitate their entry, trafficking, and replication.^44,45^ Previous research illustrate that SARS-CoV-2 spike protein S1 interacts with cholesterol and the high-density lipoprotein (HDL) scavenger receptor B type 1 (SR-B1) and promotes viral entry.^46^ Emerging evidence also suggests that plasma lipoproteins may participate in the host innate immune system. It is well known that viruses adapt various strategies to eradicate interferon signaling since it is crucial as a broad spectrum and the first line antiviral defense. SARS-CoV-2 proteins can target host proteins involved in interferon signaling, including inhibition of the translocation of IRF3 and the phosphorylation of STAT1/STAT2.^47^ However, all these well-known viral strategies were carried out ‘in-door’. The outdoor inhibitor ligands of a receptor, e.g., the WNT signaling, are of course the most effective way to balance the signaling activation and silence. Targeting IFNAR1 outside the cell membrane would be one of the most obvious and effective ways to facilitate host cell defense evasion, including interfering with IFN receptor transcription, blocking post-translational modifications, and IFNAR1 degradation.^47–50^ However, such an inhibitory ligand in interferon signaling has not yet been reported. In this study, we demonstrated a new ‘out-door’ mechanism of how virus-stimulated secretion of LRPAP1 acts as an inhibitory ligand to bind and induce IFNAR1 degradation (Fig. 6m), and further revealed the N-terminal region to interact with IFNAR1. More importantly, a synthesized peptide derived from the N-terminal sequence, RAPD1P1, potently inhibited IFNAR1 degradation and promoted viral infections. These results emphasized the importance of lipoprotein in viral infections and provide a future research direction.

LRPAP1 dependent IFNAR1 degradation may serve as a common way to evade host defense by both DNA and RNA viruses. We discovered significantly decreased infections after silencing LRPAP1, including picornavirus, coronavirus, flavivirus, herpesvirus, and hepadnavirus. Further investigating the function of LRPAP1 in innate immunity could provide novel insights into the pharmaceutical and clinical treatment options. The abundant plasma α2M protein (240 ~ 290 mg/100 mL in adult’s blood), a natural LRPAP1 inhibitor that is generated in the liver and involved in the clearance of the amyloid-beta peptide in the brain and reduction of cartilage degrading factors,^51–53^ is an FDA-approved drug for treating arthritis and other orthopedic problems.^54^ We showed promising antiviral effects of α2M at dosages within the biosafety range (<1.5 mg/100 ml) challenged by both DNA and RNA viruses. We expect that LRPAP1 inhibitors could be rapidly developed and used for the treatment of multiple viral and bacterial infections.

It is reported that LRPAP1 assists LRPs’ maturation and acts as a strong antagonist for LRPs on the cell membrane.^55,56^ The elevated LRPAP1 suppresses the extracellular domain of LRPs where the binding and internalization of ligands (e.g., amyloid-beta, apolipoprotein B- and apolipoprotein E -enriched LDL cholesterol) are ensuing inhibited.^22,57^ Ritonavir, an HIV protease inhibitor, directly increases apoB production and secretion through inhibiting apoB degradation,^58^ indicating the importance of viral proteases on LRPAP1 and lipid metabolism. We detected a decreased LRP1 level, reversely correlated with the increased LRPAP1 level followed by a viral infection or viral protease expression (e.g. 2A^pro^ and 3CL^pro^), suggesting that the interaction between proteases and LRPAP1 interferes with LRP1 maturation, triggering a compensation for the continued production of LRPAP1. LRP1 can activate Notch signaling and contribute to many diseases including cancers.^23^ We detected a decreased Notch level upon EV71 or ZIKV infections (Supplementary Fig. S6s, t). Our findings not only confirmed the crosstalk between LRP1 and Notch signaling pathway, but also provided new insights into Notch signaling upon virus infections.

In conclusion, we identify that viral proteases, EV71 2A^pro^ and SARS-CoV-2 3CL^pro^, promote the extracellular level of LRPAP1, which degrades IFNAR1 and inhibits the following interferon signaling. Moreover, inhibiting LRPAP1 by α2M decreases both RNA and DNA viral infections. α2M is expected as a pan-antiviral agent in clinical settings through the development of proper delivery systems. Therefore, we not only expand the relationship between lipoprotein metabolism and host innate immune response but also pave the way for the development of LRPAP1 inhibitors as pan-antiviral drugs.

## Materials and methods

### Cells and viruses

Rhabdomyosarcoma (RD), human embryonic kidney 293 cells with SV40 large T antigen (HEK-293T), adenocarcinomic human alveolar basal epithelial cells (A549), African green monkey kidney epithelial cells (Vero), HepG2 cells with inducible HBV (HepAD38), and murine mammary carcinoma cells (4T1) were maintained in Dulbecco’s modified Eagle’s medium (DMEM) containing 10% fetal bovine serum (FBS) with 100 U/ml penicillin and 100 μg/ml streptomycin. EV71 (SHZH98 strain; GenBank accession number AF302996.1.^42^) was obtained from the Shenzhen Center for Disease Control and Prevention (CDC), Shenzhen, China. HSV-1 (VR-1493™), HCoV-OC43 (VR-1558™), and ZIKV (VR-84™) were bought from ATCC. To prepare virus stocks, viruses were propagated on 90% confluent monolayer cells in DMEM with 2% FBS as described previously.^16^

### Plasmids and mutation

LRPAP1 was generated with pcDNA3.1 (Invitrogen) backbone for expression in mammalian cells, while pQTEV-LRPAP1 (Addgene) was used for the expression of recombinant protein. Ubiquitin and LRPAP1 mutations were generated with the pCMV (Invitrogen) backbone. IFNAR1 and viral proteins (2 A, 3 C, and 3D) expressing vectors were generated with the pcDNA4/His B (Invitrogen) backbone. Each cDNA fragment was directly cloned into the Not I and Xba I sites of the vector. Mutation of Cys^110^ to Ala^110^ of 2 A^pro^ (yielding 2 A^C110A^) was carried out by site-directed mutagenesis with a one-step mutagenesis kit (Invitrogen). Primer sequences for the constructions are available upon request. The SARS-CoV-2 genes were generated with pCDH-CMV backbone,^59^ which were kindly provided by Dr. Peihui Wang (Shandong University).

### Recombinant protein purification

The expression construct of 2A protease (pET28a-2A), 2A mutant (pET28a-2Am) or LRPAP1 (pQTEV-LRPAP1) was introduced into Lemo21 competent *E. coli* according to the protocol of NEB. The bacteria were cultured at 37 °C until the OD_600_ reached 0.4–0.6. Then the bacteria were incubated overnight at 16 °C with the addition of isopropyl-β-D-thiogalactopyranoside (IPTG) at a concentration of 0.4 mM to induce protein expression. Cell lysates were obtained by French Press at 1000 kPa at 4 °C. The lysates were collected by centrifugation at 20,000 rpm at 4 °C. The following protein purification was conducted as Ni-NTA Purification Protocol (QIAGEN). The purified protein was stock in a modified protein stabilization buffer (1 mM DTT, 0.5 mM EDTA, 50 mM NaCl, 50 mM Tris-HCl pH8.0, 35% (v/v) glycerol) at −20 °C.

### Peptides

The recombinant extracellular domain of IFNAR1 (ECD-IFNAR1) was bought from Sino Biological (13222-H08H). Peptides RAPD1P1 (*YSREKNQPKPSPKRESGEE*), and RAPD1P2 (*FRMEKLNQLWEKAQRLHLPPV*), scramble peptide (control ligand), and cell penetrating peptide RV5 (*RGDFV*) were synthesized by GenScript Biotech with a purity of 95%.

### Animal study

The C57BL/6 mice were purchased and were bred in the City University of Hong Kong. All animal experiment protocols were approved by the Ethics Committee of City University of Hong Kong with the license from Department of Health, Hong Kong (18-69 in DH/SHS/8/2/5 Pt.3). The studies were performed in accordance with the approved protocol. The studies were performed in accordance with the approved protocol. New born C57BL/6 mice were intraperitoneal injected with EV71 (2*10^8^ PFU) or EV71 + rLRPAP1 (200 nM) twice per week. The neurological status and behavior of each mouse was measured every day. On day 7, mice were sacrificed, and the intestine and blood were collected for viral testing.

### Virus infection

Viral infection in vitro was performed as previous described.^16^ Briefly, cells were washed twice with PBS and infected with EV71 or the indicated viruses at the multiplicity of infection (MOI) indicated in the figure legends. After adsorption for 1 h, the inoculum was removed, and cells were washed twice to remove the unattached viruses; thereafter the culture medium was added. For viral entry and uncoating assays, the inoculum and cells were harvested after the indicated period. For the in vivo test, newborn mice were intraperitoneal injected with rLRPAP1 (200 nM), EV71 (2*10^8^ PFU), or EV71 with rLRPAP1 twice per week.

### Western blot

Total cellular proteins were prepared using radioimmunoprecipitation assay (RIPA) buffer (50 mM Tris-HCl, pH 7.5, 150 mM NaCl, 1 mM EDTA, 1% Triton X-100, 0.1% SDS, 1× Roche protease inhibitor cocktail, 1× Roche Phostop) with occasional vortexing. Then proteins were subjected to Western blotting. The protein mentioned in Western blot analysis were detected with specific antibodies against VP1 (Abnova, PAB7631-D01P), ZIKV-Env (GeneTex, GTX133314), HSV-Env (Invitrogen, PA5-38569), HBx (Santa Cruz Biotechnology, sc-57760), HBs (EXBIO, 11-329-C100), HBc (Santa Cruz Biotechnology, sc-23947), LRP1 (Abcam, ab92544), LRPAP1 (Abcam, ab76500), IFNAR1 (Abcam, ab45172), eIF4G (Santa Cruz Biotechnology, sc-11373) and Ubquitin (Santa Cruz Biotechnology, sc-8017). Hes1 (Abcam, ab71559). Target proteins were detected with corresponding secondary antibodies (Santa Cruz Biotechnology) and finally visualized by color development with a chemiluminescence detection system (Amersham Biosciences).

### The mRNA extraction and qRT-PCR

The mRNA of indicated cells was extracted by RNAiso Plus (TaKaRa) following with the related protocol (Cat. #9108). The total RNA was reverse transcribed into cDNA by using a one-step reverse transcription system (Takara, Cat. #RR024A). Quantitative reverse transcription-PCR (qRT-PCR) was carried out by using an ABI 7500 Real-Time PCR system with SYBR green Master Mix (Applied Biosystems). The PCR was set up under the following thermal cycling conditions: 45 cycles of 95 °C for 30 s and 60 °C for 5 s. Fluorescence signals were collected by the machine during the extension phase of each PCR cycle. The threshold cycle (*C*_T_) value of each protein was normalized to that of glyceraldehyde-3-phosphate dehydrogenase (GAPDH). The qRT-PCR was performed by using indicated primer pairs (Table 1).

**Table 1:** The primers of the indicated genes

	sense(5′-3′)	antisense(5′-3′)
LRPAP1	GGACGAACTCGCCTGGAAGAAA	TTCCGTCCAGACCATACTTGGC
GAPDH	GATTCCACCCATGGCAAATTCCA	GGTGATGGGATTTCCATTGATGA
IFNAR1	TCCGCGTACAAGCATCTGAT	GACTGTTTTGGAGCACCGATA
IFN-α1	GCCTCGCCCTTTGCTTTACT	GGATCAGCTCATGGAGGACAGA
IFN-β1	CTTGGATTCCTACAAAGAAGCAGC	TCCTCCTTCTGGAACTGCTGCA
ISG15	ATGGGCTGGGACCTGACG	GCCAATCTTCTGGGTGATCTG
MxA	GCTTGCTTTCACAGATGTTTCG	AAGGGATGTGGCTGGAGATG
OASL	TCCACCTGCTTCACAGAACTACA	TGGGCTGTGTTGAAATGTGTTT
EV71 VP1^41^	GCAGCCCAAAAGAACTTCAC	ATTTCAGCAGCTTGGAGTGC
HSV-1 UL30	AGCCTGTACCCCAGCATCAT	TGGGCCTTCACGAAGAACA
HCoV-OC43 (NSP1)	TTGTGAGCGATTTGCGTGCG	ACACGTCCCTGGCTGAAAGC
ZIKV (Envelope)	TGCCCAACACAAGGTGAAGC	ACTGACAGCATTATCCGGTACTC

### Immunofluorescent imaging

Cellular expression of IFNAR1, viral protein VP1 and LRPAP1 at HEK-293T cells with and without the stimulation of EV71, the recombinant LRPAP1, and overexpression of LRPAP1 or 2A, were examined by the following protocol. Briefly, HEK-293T cells were washed twice with PBS and fixed with 4% paraformaldehyde for 20 min. Afterwards, cells were incubated with blocking buffer (10% (v/v) FBS, 1% (v/v) BSA, and 0.1% (v/v) Triton in PBS) for 2 h. Cells were then incubated with specific antibodies. The viral structural protein VP1, LRPAP1 and IFNAR1 were detected with specific antibodies against VP1 (Abnova, PAB7631-D01P), LRPAP1 (Atlas, HPA008001) and IFNAR1 (Novus, NBP1-83119). After washing with PBST (PBS + 0.1% Tween-20) for 3 times, cells were labeled with Alexa Fluor 488 (green), or Alexa Fluor 594 (red) conjugated secondary antibody (dilution of 1:1000) (Life Technologies) for 1 h at 4 °C with shaking. After three times washing with PBST (PBS + 0.1% Tween-20), cells were followed by nuclei staining with DAPI (0.5 μg/ml) for 5 min at room temperature. Finally, cells were mounted with Prolong Gold Antifade Reagent on slides. Images were taken by Carl Zeiss LSM 880 confocal microscope using 63× oil objective and analyzed by ZEN software.

As for live-cell imaging, HEK-293T cells were transfected with pcDH-eGFP-IFNAR1 for 48 h. Then cells were pre-stained with the cell membrane dye, CellMask (C10046, Invitrogen™) and nuclei dye, Hochest 33342 (62249, Thermo Scientific™). Cells were then pretreated with PBS or 200 nM rLRPAP1 on ice for 30 min. The live cells were continuously recorded in the Thermo fisher cell insight (CX7) linked with CO2 unit at 37 °C for 1 h.

### Co-immunoprecipitation

The interaction of LRPAP1, LRP1 and IFNAR1 was examined by immunoprecipitation using a protocol described as followed. HEK293T cells were cultured at around 90% confluence in a 100 mm tissue culture dish. Cells were washed with 10 ml of PBS and harvested in 0.5 ml of ice-cold lysis buffer (0.15 mM NaCl, 0.05 mM tris-HCl, pH 7.4, 1% SDS, 1% NP-40) and Protease Cocktail (Roche) and PhoSTOP (Roche). The total extract was pre-cleared with 10 μg normal rabbit IgG (Cell signaling, 2729 S) and 20 μl of protein A/G plus-agarose (Santa Cruz Biotechnology) at 4 °C on a rotor for 1 h. After gently removed protein A/G agarose beads by centrifugation at 500 rcf for 2 min, 0.2 mg of total extract was immunoprecipitated with the indicated antibodies overnight at 4 °C on a rotor. Approximately 20 μl of protein A/G plus-agarose (Santa Cruz Biotechnology) was added at 4 °C for one hour. Then, the beads were washed three times with washing buffer (lysis buffer contained 0.1% NP-40). The pellet was suspended with 40 μl PBS and 10 μl of 5× SDS-PAGE loading buffer and the samples were boiled for 10 min. The supernatant was collected and 20 μg of lysate was loaded onto 10% acrylamide gene electrophoresis and immunoblotting was performed using antibodies against IFNAR1 (Abcam, ab45172), LRP1 (Abcam, ab92544), LRPAP1 (Abcam, ab14404) as described before.

### Protein docking

The experimentally-determined 3D structures of LRPAP1 (2p03) and IFNAR1(3s98) were collected from Protein Data Bank (PDB) archive. The prediction of protein-protein docking was measured by H-dock (http://hdock.phys.hust.edu.cn/). The docking model was selected by the confidence score. A score above 0.7 indicates that the two proteins have a very high binding potential.

### Flow cytometry assay

The surface IFNAR1 was monitored by flow cytometry as previous described.^60^ Briefly, murine breast cancer cells (4T1) were stained with APC-conjugated IFNAR1 (Biolegend #127314, 1:100) on ice for 30 min. Cells were then treated with or without rLRPAP1 (200 nM) for the indicated time. Fluorescence-activated cells were then sorted and analyzed by Beckman Coulter CytoFLEX S Flow cytometer (Becton Dickinson). The result was analyzed by FlowJo (version 10.5.3).

### Membrane and cytosol protein isolation

Tissues were washed twice with PBS and made into homogenate by 2 ml grinder. Membrane proteins were separated from cytosol protein extraction by using the protocol of membrane protein extraction kit (89842, Thermo).

### LRPAP1 stimulated mouse brain ex vivo

An acutely prepared C57BL/6 mouse’s brain was separated into two hemispheres, following with an immediately incubation in the oxygen supplied cuvette containing freshly prepared artificial cerebrospinal fluid (ACSF) (127 mM NaCl, 1 mM KCl, 1.2 mM KH_2_PO_4_, 26 mM NaHCO_3_, 10 mM D-glucose) or ACSF plus 200 nM rLRPAP1 at 0 °C for 1 h.

### Microscale thermophoresis (MST) analysis

The polyhistidine (His)-tag peptides, the recombinant 2A, the recombinant 2Am, and ECD-IFNAR1, were incubated with the RED-tris-NTA 2nd Generation dye (Cat#MO-L018, Nanotemper-Technologies) for 30 min, respectively. Then these fluorescently labeled peptides were mixed with the serial diluted ligands, including the scramble peptide, the commercial penetrating peptide (RV5), LRPAP1 peptides (RAPD1P1, RAPD1P2), or rLRPAP1 without His-tag, respectively. The mixtures were captured by Monolith^TM^ NT.115 Series capillaries (Cat# MO-K022). Capillaries were then subjected to Monolith NT for MST analysis. According to Monolith guidelines, signal-to-noise (S/N) ratios greater than 5.0 were indicated as the valid binding.

### Statistical analysis

Results were expressed as mean ± standard deviation (SD). A two-tailed Student *t* test was applied for two-group comparisons. A *P* value of <0.05 was considered statistically significant.

### Supplementary information

Supplementary Video 3 Live-cell images with PBS

Supplementary Video 4 Live-cell images with rLRPAP1

Mice with EV71 injection

with EV71 and rLRPAP1 injection

Supplementary Materials

## Data Availability

Data are presented in the main text or supplementary figures. Other supporting data are also available at request from the corresponding author.
