# Supplementary material for: Secreted LRPAP1 binds and triggers IFNAR1 degradation to facilitate virus evasion from cellular innate immunity
Source: Signal Transduct Target Ther. 2023 Sep 25;8:374. doi: 10.1038/s41392-023-01630-1 (PMC10518340; doi:10.1038/s41392-023-01630-1)

Supplementary Materials for

**Secreted LRPAP1 binds and triggers IFNAR1 degradation to facilitate virus evasion from cellular innate immunity**

Huangcan Li^1,3,#^, Xiong Wang^1,#^, Yiran Wang^1,#^, Yichen Li^1^, Ying Chen^1^, Yin-Ting Wong^2^, Jufang He^1,2^, Ming-Liang He^1,3, *^

Correspondence to: Ming-liang He [minglihe@cityu.edu.hk](mailto:minglihe@cityu.edu.hk) or [mlhe7788@gmail.com](mailto:mlhe7788@gmail.com)

**This PDF file includes:**

Materials and Methods

Figures. S1 to S6

Tables S1 to S3

Captions for Movies S1 to S4

**Other Supplementary Materials for this manuscript include the following:**

Movies S1 to S4

**Materials and Methods**

Cells and viruses

Rhabdomyosarcoma (RD), human embryonic kidney 293 cells with SV40 large T antigen (HEK-293T), adenocarcinomic human alveolar basal epithelial cells (A549), African green monkey kidney epithelial cells (Vero), HepG2 cells with inducible HBV (HepAD38), and murine mammary carcinoma cells (4T1) were maintained in Dulbecco’s modified Eagle’s medium (DMEM) containing 10% fetal bovine serum (FBS) with 100 U/ml penicillin and 100 μg/ml streptomycin. EV71 (SHZH98 strain; GenBank accession number AF302996.1^43^.) was obtained from the Shenzhen Center for Disease Control and Prevention (CDC), Shenzhen, China. HSV-1 (VR-1493™), HCoV-OC43 (VR-1558™) and ZIKV (VR-84™) were bought from ATCC. To prepare virus stocks, viruses were propagated on 90% confluent monolayer cells in DMEM with 2% FBS as described previously^16^.

### Plasmids and mutation

LRPAP1 was generated with pcDNA3.1 (Invitrogen) backbone for expression in mammalian cells, while pQTEV-LRPAP1 (Addgene) was used for the expression of recombinant protein. Ubiquitin and LRPAP1 mutations were generated with the pCMV (Invitrogen) backbone. IFNAR1 and viral proteins (2A, 3C, and 3D) expressing vectors were generated with the pcDNA4/His B (Invitrogen) backbone. Each cDNA fragment was directly cloned into the Not I and Xba I sites of the vector. Mutation of Cys^110^ to Ala^110^ of 2A^pro^ (yielding 2A^C110A^) was carried out by site-directed mutagenesis with a one-step mutagenesis kit (Invitrogen). Primer sequences for the constructions are available upon request. The SARS-CoV-2 genes were generated with pCDH-CMV backbone^62^, which were kindly provided by Dr. Peihui Wang (University of Shang Dong).

### Recombinant protein purification

The expression construct of 2A protease (pET28a-2A), 2A mutant (pET28a-2Am) or LRPAP1 (pQTEV-LRPAP1) was introduced into Lemo21 competent *E. coli* according to the protocol of NEB. The bacteria were cultured at 37℃ until the OD_600_ reached 0.4-0.6. Then the bacteria were incubated overnight at 16 °C with the addition of isopropyl-β-D-thiogalactopyranoside (IPTG) at a concentration of 0.4 mM to induce protein expression. Cell lysates were obtained by French Press at 1000 kPa at 4°C. The lysates were collected by centrifugation at 20,000rpm at 4°C. The following protein purification was conducted as Ni-NTA Purification Protocol (QIAGEN). The purified protein was stock in a modified protein stabilization buffer (1mM DTT, 0.5mM EDTA, 50mM NaCl, 50mM Tris-HCl pH8.0, 35% (v/v) glycerol) at -20°C.

### Peptides

The recombinant extracellular domain of IFNAR1 (ECD-IFNAR1) was bought from Sino Biological (13222-H08H). Peptides RAPD1P1 (*YSREKNQPKPSPKRESGEE*), and RAPD1P2 (*FRMEKLNQLWEKAQRLHLPPV*), scramble peptide (control ligand), and cell penetrating peptide RV5 (*RGDFV*) were synthesized by GenScript Biotech with a purity of 95%.

**Animal study**

The C57BL/6 mice were purchased and were bred in the City University of Hong Kong. All animal experiment protocols were approved by the Ethics Committee of City University of Hong Kong with the license from Department of Health, Hong Kong (18-69 in DH/SHS/8/2/5 Pt.3). The studies were performed in accordance with the approved protocol. The studies were performed in accordance with the approved protocol. New born C57BL/6 mice were intraperitoneal injected with EV71 (2*10^8^ PFU) or EV71+ rLRPAP1 (200 nM) twice per week. The neurological status and behavior of each mouse was measured every day. On day 7, mice were sacrificed, and the intestine and blood were collected for viral testing.

### Virus infection

Viral infection *in vitro* was performed as previous described^16^. Briefly, cells were washed twice with PBS and infected with EV71 or the indicated viruses at the multiplicity of infection (MOI) indicated in the figure legends. After adsorption for 1 h, the inoculum was removed, and cells were washed twice to remove the unattached viruses; thereafter the culture medium was added. For viral entry and uncoating assays, the inoculum and cells were harvested after the indicated period. For the *in vivo* test, newborn mice were intraperitoneal injected with rLRPAP1 (200 nM), EV71 (2*10^8^ PFU), or EV71 with rLRPAP1 twice per week.

**Western blot**

Total cellular proteins were prepared using radioimmunoprecipitation assay (RIPA) buffer (50 mM Tris-HCl, pH 7.5, 150 mM NaCl, 1 mM EDTA, 1% Triton X-100, 0.1% SDS, 1× Roche protease inhibitor cocktail, 1× Roche Phostop) with occasional vortexing. Then proteins were subjected to Western blotting. The protein mentioned in Western blot analysis were detected with specific antibodies against VP1 (Abnova, PAB7631-D01P), ZIKV-Env (GeneTex, GTX133314), HSV-Env (Invitrogen, PA5-38569), HBx (Santa Cruz Biotechnology, sc-57760), HBs (EXBIO, 11-329-C100), HBc (Santa Cruz Biotechnology, sc-23947), LRP1 (Abcam, ab92544), LRPAP1 (Abcam, ab76500), IFNAR1 (Abcam, ab45172), eIF4G (Santa Cruz Biotechnology, sc-11373) and Ubquitin (Santa Cruz Biotechnology, sc-8017). Hes1 (Abcam, ab71559). Target proteins were detected with corresponding secondary antibodies (Santa Cruz Biotechnology) and finally visualized by color development with a chemiluminescence detection system (Amersham Biosciences).

### The mRNA extraction and qRT-PCR

The mRNA of indicated cells was extracted by RNAiso Plus (TaKaRa) following with the related protocol (Cat. #9108). The total RNA was reverse transcribed into cDNA by using a one-step reverse transcription system (Takara, Cat. #RR024A). Quantitative reverse transcription-PCR (qRT-PCR) was carried out by using an ABI 7500 Real-Time PCR system with SYBR green Master Mix (Applied Biosystems). The PCR was set up under the following thermal cycling conditions: 45 cycles of 95°C for 30 s and 60°C for 5 s. Fluorescence signals were collected by the machine during the extension phase of each PCR cycle. The threshold cycle (*C*_T_) value of each protein was normalized to that of glyceraldehyde-3-phosphate dehydrogenase (GAPDH). The qRT-PCR was performed by using indicated primer pairs (Table 1).

**Table 1. The primers of the indicated genes.**

|  | sense（5'-3'） | antisense（5'-3'） |
| --- | --- | --- |
| LRPAP1 | GGACGAACTCGCCTGGAAGAAA | TTCCGTCCAGACCATACTTGGC |
| GAPDH | GATTCCACCCATGGCAAATTCCA | GGTGATGGGATTTCCATTGATGA |
| IFNAR1 | TCCGCGTACAAGCATCTGAT | GACTGTTTTGGAGCACCGATA |
| IFN-α1 | GCCTCGCCCTTTGCTTTACT | GGATCAGCTCATGGAGGACAGA |
| IFN-β1 | CTTGGATTCCTACAAAGAAGCAGC | TCCTCCTTCTGGAACTGCTGCA |
| ISG15 | ATGGGCTGGGACCTGACG | GCCAATCTTCTGGGTGATCTG |
| MxA | GCTTGCTTTCACAGATGTTTCG | AAGGGATGTGGCTGGAGATG |
| OASL | TCCACCTGCTTCACAGAACTACA | TGGGCTGTGTTGAAATGTGTTT |
| EV71 VP1^42^ | GCAGCCCAAAAGAACTTCAC | ATTTCAGCAGCTTGGAGTGC |
| HSV-1 UL30 | AGCCTGTACCCCAGCATCAT | TGGGCCTTCACGAAGAACA |
| HCoV-OC43  (NSP1) | TTGTGAGCGATTTGCGTGCG | ACACGTCCCTGGCTGAAAGC |
| ZIKV (Envelop) | TGCCCAACACAAGGTGAAGC | ACTGACAGCATTATCCGGTACTC |

**Immunofluorescent imaging**

Cellular expression of IFNAR1, viral protein VP1 and LRPAP1 at HEK-293T cells with and without the stimulation of EV71, the recombinant LRPAP1, and overexpression of LRPAP1 or 2A, were examined by the following protocol. Briefly, HEK-293T cells were washed twice with PBS and fixed with 4% paraformaldehyde for 20 min. Afterwards, cells were incubated with blocking buffer (10% (v/v) FBS, 1% (v/v) BSA, and 0.1% (v/v) Triton in PBS) for 2 h. Cells were then incubated with specific antibodies. The viral structural protein VP1, LRPAP1 and IFNAR1 were detected with specific antibodies against VP1 (Abnova, PAB7631-D01P), LRPAP1 (Atlas, HPA008001) and IFNAR1 (Novus, NBP1-83119). After washing with PBST (PBS+0.1% Tween-20) for 3 times, cells were labeled with Alexa Fluor 488 (green), or Alexa Fluor 594 (red) conjugated secondary antibody (dilution of 1:1000) (Life Technologies) for 1 h at 4°C with shaking. After three times washing with PBST (PBS+0.1% Tween-20), cells were followed by nuclei staining with DAPI (0.5 μg/ml) for 5 minutes at room temperature. Finally, cells were mounted with Prolong Gold Antifade Reagent on slides. Images were taken by Carl Zeiss LSM 880 confocal microscope using 63×oil objective and analyzed by ZEN software.

As for live-cell imaging, HEK-293T cells were transfected with pcDH-eGFP-IFNAR1 for 48h. Then cells were pre-stained with the cell membrane dye, CellMask (C10046, Invitrogen™) and nuclei dye, Hochest 33342 (62249, Thermo Scientific™). Cells were then pretreated with PBS or 200 nM rLRPAP1 on ice for 30 min. The live cells were continuously recorded in the Thermo fisher cell insight (CX7) linked with CO2 unit at 37°C for 1 h.

### Co-immunoprecipitation

The interaction of LRPAP1, LRP1 and IFNAR1 was examined by immunoprecipitation using a protocol described as followed. HEK293T cells were cultured at around 90% confluence in a 100 mm tissue culture dish. Cells were washed with 10 ml of PBS and harvested in 0.5 ml of ice-cold lysis buffer (0.15 mM NaCl, 0.05 mM tris-HCl, pH 7.4, 1% SDS, 1% NP-40 and Protease Cocktail (Roche) and PhoSTOP (Roche). The total extract was pre-cleared with 10 μg normal rabbit IgG (Cell signaling, 2729S) and 20 μl of protein A/G plus-agarose (Santa Cruz Biotechnology) at 4°C on a rotor for 1 h. After gently removed protein A/G agarose beads by centrifugation at 500 rcf for 2 min, 0.2 mg of total extract was immunoprecipitated with the indicated antibodies overnight at 4°C on a rotor. Approximately 20 μl of protein A/G plus-agarose (Santa Cruz Biotechnology) was added at 4°C for one hour. Then, the beads were washed three times with washing buffer (lysis buffer contained 0.1% NP-40). The pellet was suspended with 40 μl PBS and 10 μl of 5x SDS-PAGE loading buffer and the samples were boiled for 10 min. The supernatant was collected and 20 μg of lysate was loaded onto 10% acrylamide gene electrophoresis and immunoblotting was performed using antibodies against IFNAR1 (Abcam, ab45172), LRP1 (Abcam, ab92544), LRPAP1 (Abcam, ab14404) as described before.

**Protein docking**

The experimentally-determined 3D structures of LRPAP1 (2p03) and IFNAR1(3s98) were collected from Protein Data Bank (PDB) archive. The prediction of protein-protein docking was measured by H-dock (http://hdock.phys.hust.edu.cn/http://hdock.phys.hust.edu.cn/). The docking model was selected by the confidence score. A score above 0.7 indicates that the two proteins have a very high binding potential.

**Flow cytometry assay**

The surface IFNAR1 was monitored by flow cytometry as previous described^63^. Briefly, murine breast cancer cells (4T1) were stained with APC-conjugated IFNAR1 (Biolegend #127314, 1:100) on ice for 30 min. Cells were then treated with or without rLRPAP1 (200 nM) for the indicated time. Fluorescence-activated cells were then sorted and analyzed by Beckman Coulter CytoFLEX S Flow cytometer (Becton Dickinson). The result was analyzed by FlowJo (version 10.5.3).

### Membrane and cytosol protein isolation

Tissues were washed twice with PBS and made into homogenate by 2 ml grinder. Membrane proteins were separated from cytosol protein extraction by using the protocol of membrane protein extraction kit (89842, Thermo).

### LRPAP1 stimulated mouse brain *ex vivo*

An acutely prepared C57BL/6 mouse’s brain was separated into two hemispheres, following with an immediately incubation in the oxygen supplied cuvette containing freshly prepared artificial cerebrospinal fluid (ACSF) (127 mM NaCl, 1 mM KCl, 1.2 mM KH_2_PO_4_, 26 mM NaHCO_3_, 10 mM D-glucose) or ACSF plus 200 nM rLRPAP1 at 0°C for 1 h.

### Microscale thermophoresis (MST) analysis

The polyhistidine (His)-tag peptides, the recombinant 2A, the recombinant 2Am, and ECD-IFNAR1, were incubated with the RED-tris-NTA 2^nd^ Generation dye (Cat#MO-L018, Nanotemper-Technologies) for 30 min, respectively. Then these fluorescently labeled peptides were mixed with the serial diluted ligands, including the scramble peptide, the commercial penetrating peptide (RV5), LRPAP1 peptides (RAPD1P1, RAPD1P2), or rLRPAP1 without His-tag, respectively. The mixtures were captured by Monolith^TM^ NT.115 Series capillaries (Cat# MO-K022). Capillaries were then subjected to Monolith NT. For MST analysis. According to Monolith guidelines, signal-to-noise (S/N) ratios greater than 5.0 were indicated as the valid binding.

### Statistical analysis

Results were expressed as mean ± standard deviation (SD). A two-tailed Student *t* test was applied for two-group comparisons. A *P* value of <0.05 was considered statistically significant.

**Fig. S1**


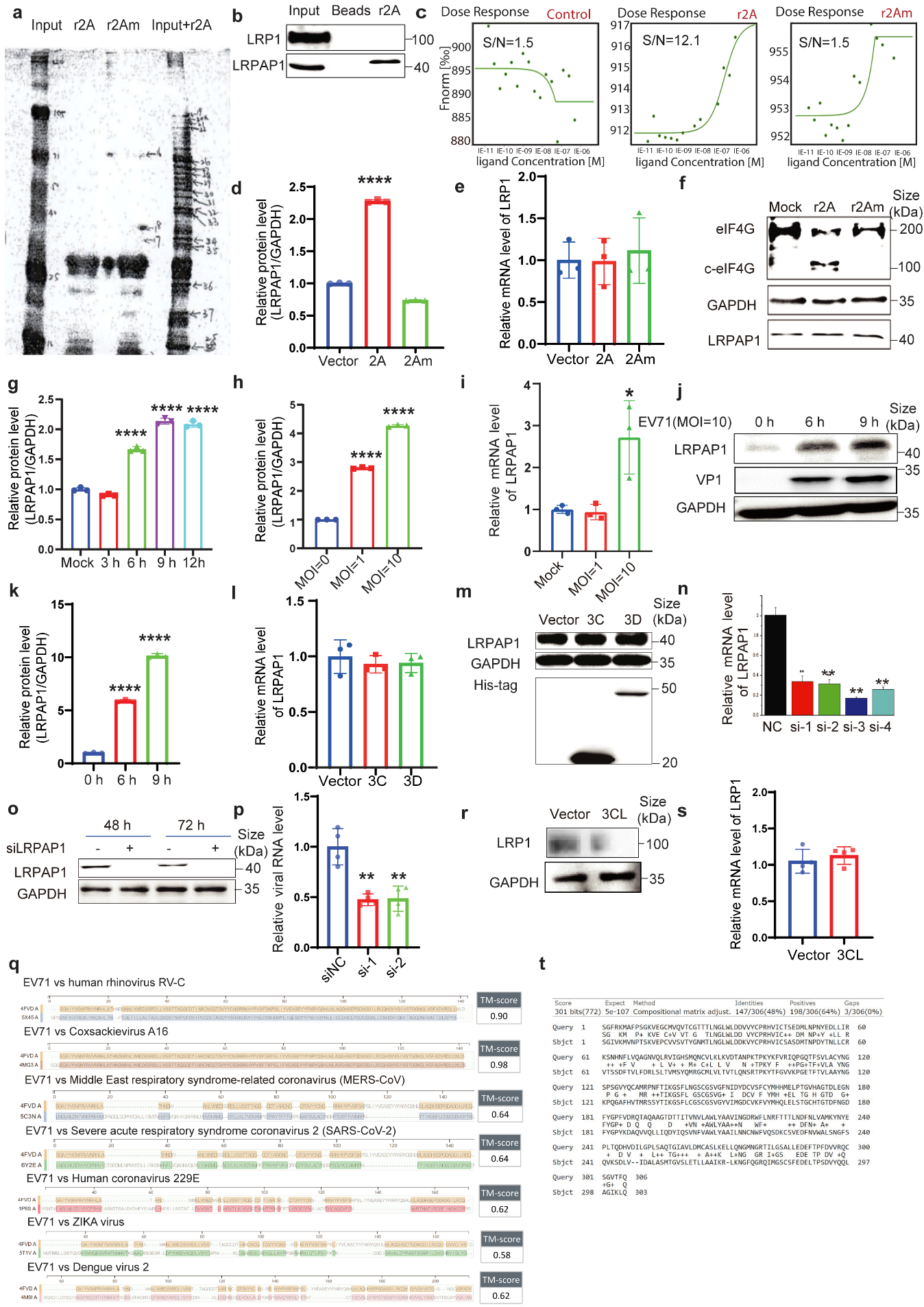


**Fig. S1.** **Viral protease 2A as well as EV71 increase LRPAP1 expression. a,** Pull-down assay for identifying 2A^pro^ interacting proteins. Proteins from recombinant 2A^pro^ (r2A) pull-down assay were separated by one-dimension PAGE-electrophoreses and visualized by silver staining. The indicated bands were cut out and applied for Mass Spectrum (MS) analysis. **b,** Immunoblots of candidate r2A-interacting protein LRPAP1. **c,** The binding affinity between the rLRPAP1 and the indicated His-tagged proteins (control peptides, r2A, and the recombinant 2A mutant (r2Am)) was analyzed by microscale thermophoresis (MST). **d,** Densitometric analysis of LRPAP1 levels in Fig. 1a was quantified and normalized with GAPDH using ImageJ. **e,** The relative mRNA level of LRP1 in HEK-293T cells transfected with pcDNA4B (vector), pcDNAB-2A or -2Am for 48 h. **f**, Immunoblots of HEK-293T cell lysates treated with the r2A or r2Am at 30°C in the cleavage buffer for 1 h. The bioactivity of r2A was determined by the cleavage of eIF4G. **g,** Densitometric analysis of LRPAP1 levels in Fig. 1d was quantified and normalized with GAPDH using ImageJ. **h**, Densitometric analysis of LRPAP1 levels in Fig. 1e was quantified and normalized with GAPDH using ImageJ. **i, j,** The RT-qPCR analysis (**i**) and the immunoblots of lysates in HEK-293T cells infected with EV71 at an MOI of 1 or 10 for the indicated time (**j**). **k,** Densitometric analysis of LRPAP1 levels in Fig. S1j was quantified and normalized with GAPDH using ImageJ. **l, m,** The relative mRNA (**l**) and protein (**m**) levels of LRPAP1 in HEK-293T cells transfected with pcDNA4B (Vector), pcDNA4B-3C or -3D for 48 h. **n, o,** The efficiency of siLRPAP1. siLRPAP1 (si-3) with the most significant effect was selected for the following experiments if it is not indicated. (**n**). The immunoblotting of LRPAP1 knockdown efficiency was estimated in RD cells after 48 h to 72 h transfection (**o**). **p,** RD cells were transfected with either siRNA (scramble RNA (NC) or siRNA-LRPAP1 (si-1 or si-2)) for 39 h and then were infected by EV71 at MOI=10 for 9 h. The relative viral RNA level was then tested. **q,** The indicated protein structures (EV71 2A, 4fvb; human rhinovirus RV-C 2A, 5x45; Coxsackievirus A16 (Cox A16) 2A, 4mg3; Middle East respiratory syndrome-related coronavirus (MERS-CoV) 3CL, 5c3n; SARS-CoV-2 3CL, 6y2e; Human coronavirus 229E 3CL, 1p9s; ZIKA virus NS2B-NS3, 5t1v; Dengue virus 2 NS2B-NS3, 4m9i) were compared via sequence independent algorithm. The highlighted amino acids indicated the aligned residue pairs. The TM-score above 0.5 means structural similarity. **r, s,** The protein (**r**) and mRNA (**s**) levels of LRP1 in HEK-293T cells transfected with empty vector pCMV, or pCMV-SARS-CoV-2-3CL for 48 h. **t,** The amino acid sequence alignment between 3CL^pro^ in SARS-CoV-2 and HCoV-OC43 by NCBI. Query indicated SARS-CoV-2 3CL^pro^, while subject was HCoV-OC43 3CL^pro^. Results were expressed as mean ± standard deviation (error bars) of at least three repeats. *P ≤ 0.05, **P ≤ 0.01, ***P ≤ 0.001, ****P ≤ 0.0001 (unpaired t-test).

**Fig. S2**

**
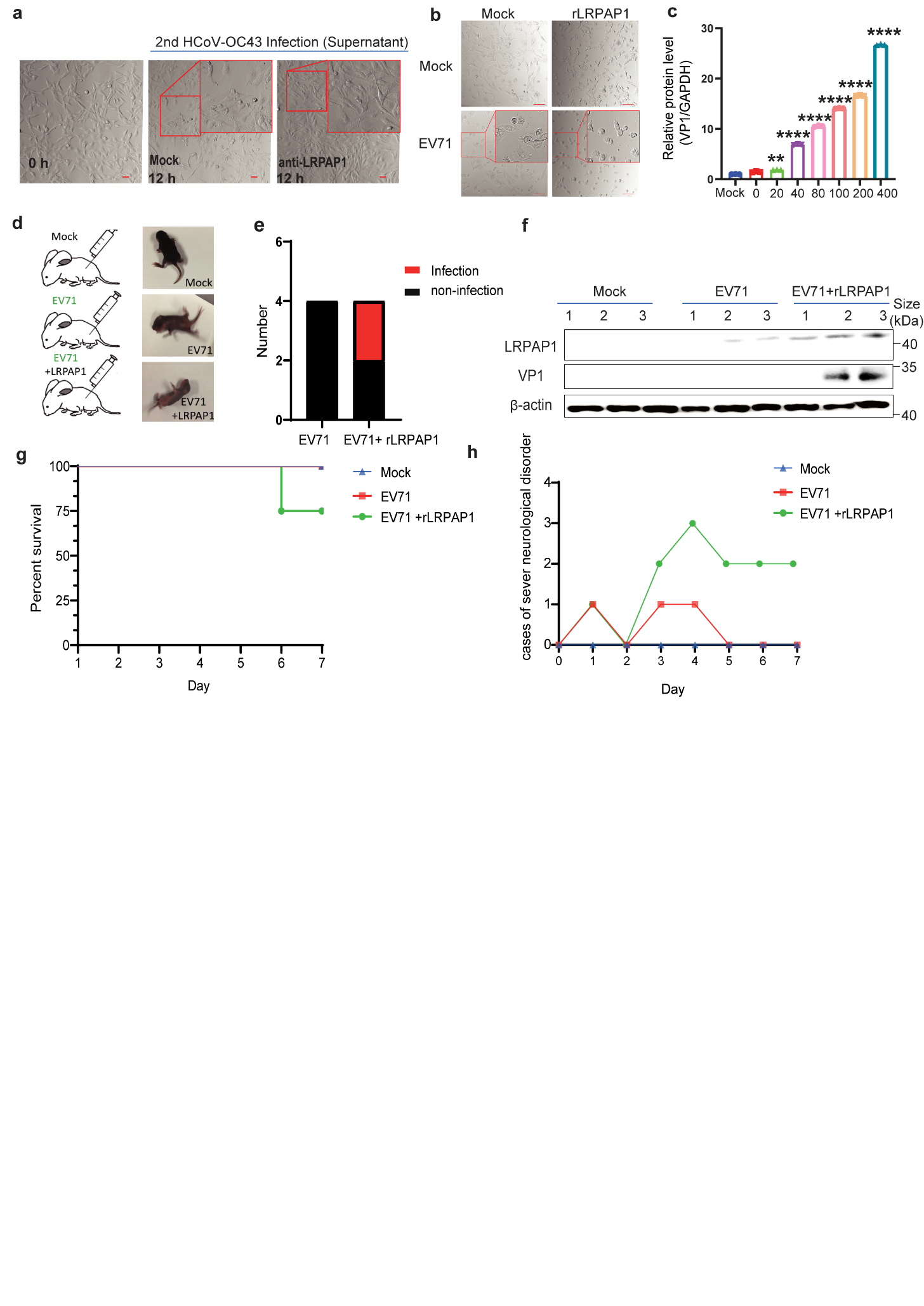
**

**Fig. S2. LRPAP1 promotes EV71 infection both *in vivo* and *in vitro*. a,** RD cells were infected with HCoV-OC43 at an MOI of 2 for 72 h. Supernatant was collected and treated with either 0.5 μg/ml antibody against LRPAP1 (anti-LRPAP1) or IgG for 1 h. The supernatant was then used to infect new RD cells for 12 h. The CPE was shown as vacuoles in the cytoplasm 12 h after treatment with mock or anti-LRPAP1 antibody (scale bar = 20 μm). **b,** The effect of rLRPAP1 on EV71 infection. RD cells were pre-treated with or without 200 nM rLRPAP1 for 1 h, and cells were then infected with EV71 at an MOI of 10 for 9 h. The CPE of EV71 was indicated by the rounding and detachment of cells (scale bar = 100 μm). **c,** Densitometric analysis of VP1 levels in Fig. 2l was quantified and normalized with GAPDH using ImageJ. **d-h,** The schematic diagram of mice after the different treatment. C57BL/6 mice were intraperitoneal injected with EV71 (2*10^8^ PFU) or EV71+ rLRPAP1 (200 nM) twice per week (**d**). The neurological status and behavior of each mouse was measured every day. On day 7, mice were sacrificed, and the intestine and blood were collected for viral testing. The number of mice, which were successfully infected or not, were determined by RT-qPCR assay from blood serum samples. Ct value less than 38 was considered as successful infection, otherwise was non-infected (**e**). The expression of viral protein (VP1) was measured from the lysates of intestine by immunoblotting (**f**). The survival rate of each group in 7 days. Compared to the other two groups, the survival rate of mice co-injected with rLRPAP1 and EV71 dropped to 75% (**g**). Number of mice with severe neurological deficits such as circling, rolling, and paralyzed legs per day. After two injections (Day 3), 50% of mice co-injected with rLRPAP1 and EV71 showed sever neurological deficits **(h)**. *P ≤ 0.05, **P ≤ 0.01, ***P ≤ 0.001, ****P ≤ 0.0001 (unpaired t-test).

**Fig. S3**

**
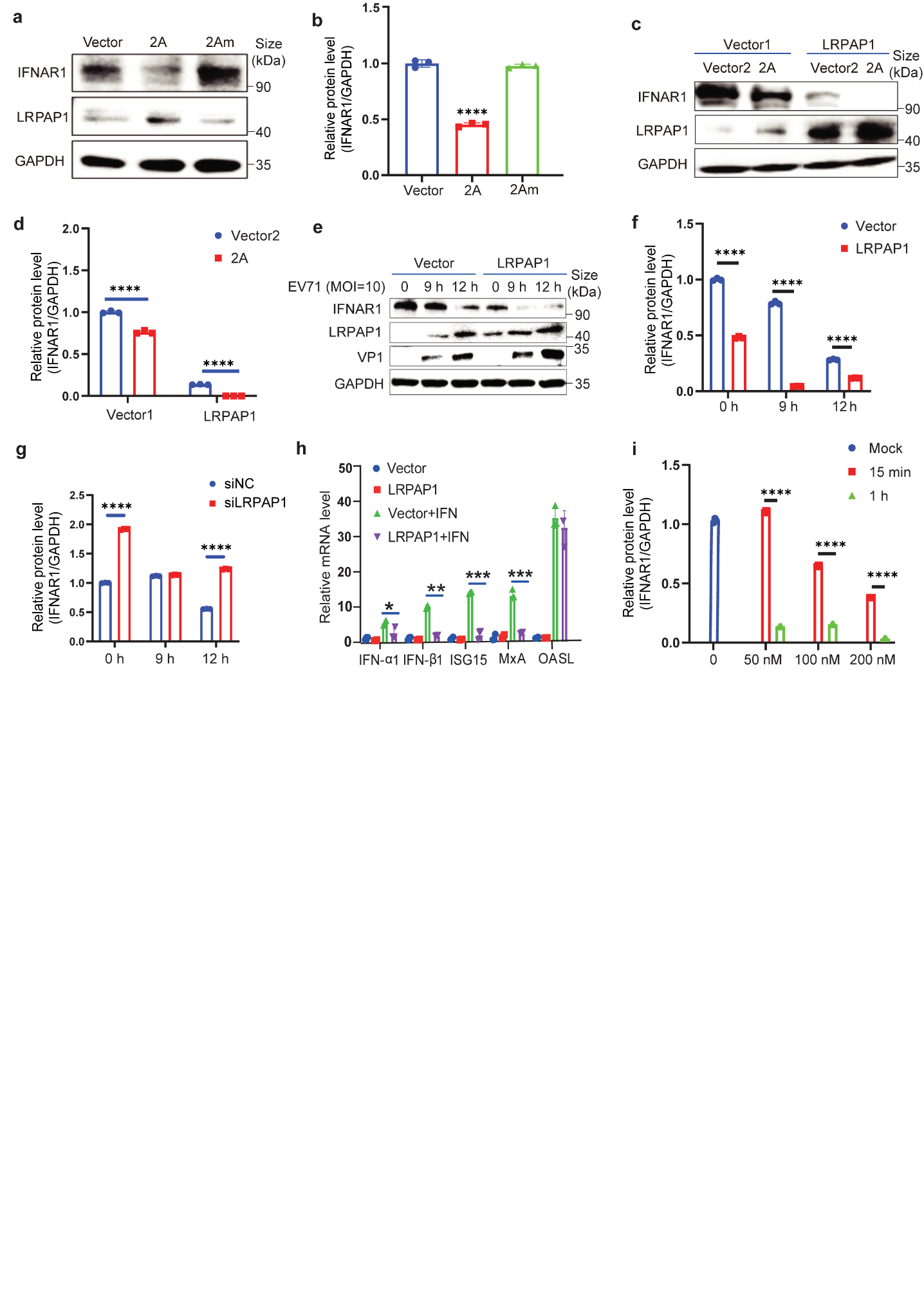
Fig. S3. Overexpression LRPAP1 inhibits type I IFN signaling via the downregulation of IFNAR1. a,** The IFNAR1 protein level in HEK-293T cells with overexpressing 2A or 2Am. **b,** Densitometric analysis of IFNAR1 levels in Fig. S3a was quantified and normalized with GAPDH using ImageJ. **c,** The IFNAR1 and LRPAP1 levels in HEK-293T cells expressed 2A alone, or both LRPAP1 and 2A simultaneously. **d,** Densitometric analysis of IFNAR1 levels in Fig. S3c was quantified and normalized with GAPDH using ImageJ. **e, f,** RD cells were transfected with pcDNA3.1 (Vector) or pcDNA-LRPAP1 for 24 h, and then infected with EV71 at an MOI of 10 for 9 h and 12 h. Western blotting was performed against IFNAR1, LRPAP1 and viral structural protein VP1 with relative antibodies (**e**). Densitometric analysis of IFNAR1 levels in Fig. S3e was quantified and normalized with GAPDH using ImageJ (**f**). **g**, Densitometric analysis of IFNAR1 levels in Fig. 3e was quantified and normalized with GAPDH using ImageJ. **h,** RD cells were transfected with pcDNA3.1 (Vector) or pcDNA-LRPAP1 for 48 h and were then treated with or without 1000 U/ml IFN-α2b for 1 h. The downstream response of type I IFN signaling pathway was evaluated by the relative mRNA level of the indicated ISGs. **i,** Densitometric analysis of IFNAR1 levels in Fig. 3i was quantified and normalized with GAPDH using ImageJ. Results were expressed as mean ± standard deviation (error bars) of at least three repeats. *P ≤ 0.05, **P ≤ 0.01, ***P ≤ 0.001, ****P ≤ 0.0001 (unpaired t-test).

**Fig. S4**


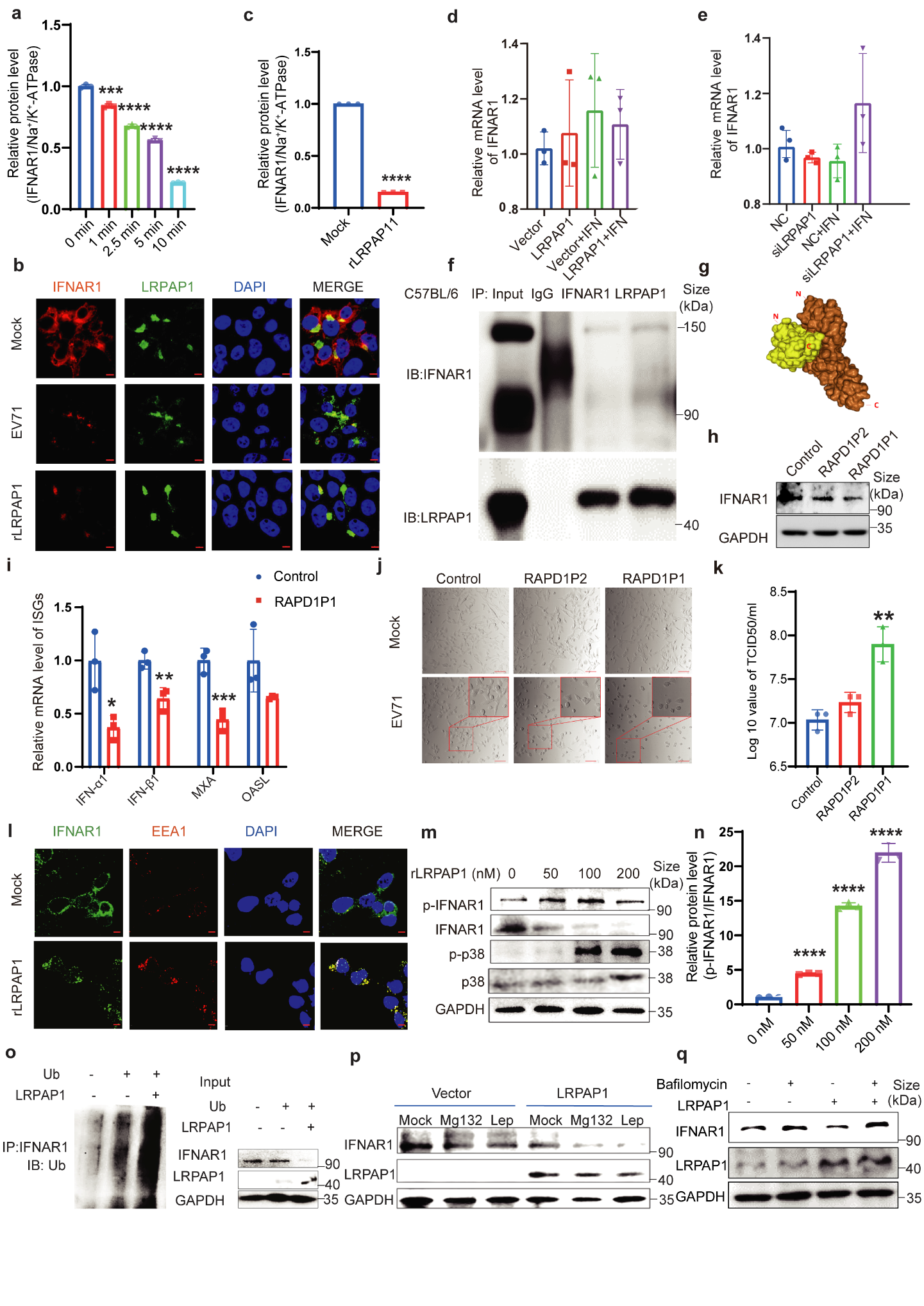


**Fig. S4. LRPAP1 binds and induces the lysosome-dependent degradation of IFNAR1.** **a,** Densitometric analysis of IFNAR1 levels in Fig. 4a was quantified and normalized with Na^+^/K^+^ ATPase using ImageJ. **b,** Immunofluorescent image of RD cells either infected with EV71 (MOI=10) for 6 h or incubated with rLRPAP1 (200 nM) for 1 h. Cells were double-stained with anti-IFNAR1 (red) and anti-LRPAP1 (green) (scale bar = 5 μm). **c,** Densitometric analysis of IFNAR1 levels in Fig. 4d was quantified and normalized with Na^+^/K^+^ ATPase using ImageJ. **d, e,** The relative mRNA level of IFNAR1 in HEK-293T cells in the context of LRPAP1 overexpression or knockdown. HEK-293T cells were transfected with pcDNA3.1 (Vector) or pcDNA- LRPAP1 (**d**) or treated with scramble siRNA (NC) or siRNA-LRPAP1 (siLRPAP1) for 48 h (**e**). Cells were then treated with or without 1000 U/ml IFN-α2b for 1 h before harvest. **f,** Immunoprecipitation of cerebrum tissue lysates from 8-week-old C57BL/6 mouse. Cerebrum was homogenized by a 2 ml tissue grinder and subjected to immunoprecipitation with the antibodies of LRPAP1 and IFNAR1. **g,** The highest confidence docking model between LRPAP1 (yellow) and IFNAR1 (brown) were generated by H-dock. The confidence score for the binding model is 0.7822. **h,** The effect of LRPAP1 peptides on the expression of IFNAR1. Immunoblots of lysates in RD cells treated with the scramble peptide (Control) or the indicated peptides of LRPAP1 (1 μM) for 1 h. **i,** The effect of RAPD1P1 on the downstream response of type I IFN signaling. RD cells were treated with 1000 U/ml IFN-α2b and the indicated peptide (control or RAPD1P1) for 1 h. The downstream response of type I IFN signaling pathway was evaluated by the relative mRNA level of the indicated ISGs. **j, k,** The effect of LRPAP1 peptides on viral infection. RD cells were pre-treated with the indicated peptide for 1 h, then cells were inoculated with or without EV71(MOI=10) for 9 h. The CPE of EV71 was indicated by the rounding and detachment of cells (scale bar = 100 μm). (**j**). The supernatant from cells infected with EV71 was then subjected to viral titration (**k**). **l,** Immunofluorescent image of the early endosome marker, EEA1, and IFNAR1 in HEK-293T cells treated with or without rLRPAP1 (200 nM) for 1 h. Cells were double-stained with EEA1 (red) and IFNAR1 (green) using respective antibodies. Nuclei were visualized by DAPI and appeared in blue (scale bar = 5 μm). **m,** Immunoblots of lysates in HEK-293T cells treated with the indicated concentration of rLRPAP1 for 1 h. The phosphorylated IFNAR1 (p-IFNAR1) was correlated with the increased phosphorylation of p38 (p-p38) levels. **n,** Densitometric analysis of p-IFNAR1 levels in Fig. S4m was quantified and normalized with IFNAR1 using ImageJ. **o,** Immunoprecipitates (indicated antibodies) in HEK-293T cells overexpressing ubiquitin, LRPAP1 and IFNAR1 were analysed by immunoblotting. **p,** Immunoblots of lysates in HEK-293T cells treated with proteasome inhibitors. HEK-293T cells were transfected with pcDNA3.1 or pcDNA-LRPAP1 for 48 h. Cells were then treated with Mg132 (40 μM), or Leupeptin (Lep, 50 μM), for 4 h before harvested. **q,** Immunoblots of LRPAP1 ectopic-expressing HEK-293T cells treated with or without 1μM Bafilomycin for 4 h. Results were expressed as mean ± standard deviation (error bars) of at least three repeats. *P ≤ 0.05, **P ≤ 0.01, ***P ≤ 0.001, ****P ≤ 0.0001 (unpaired t-test).

**Fig. S5**


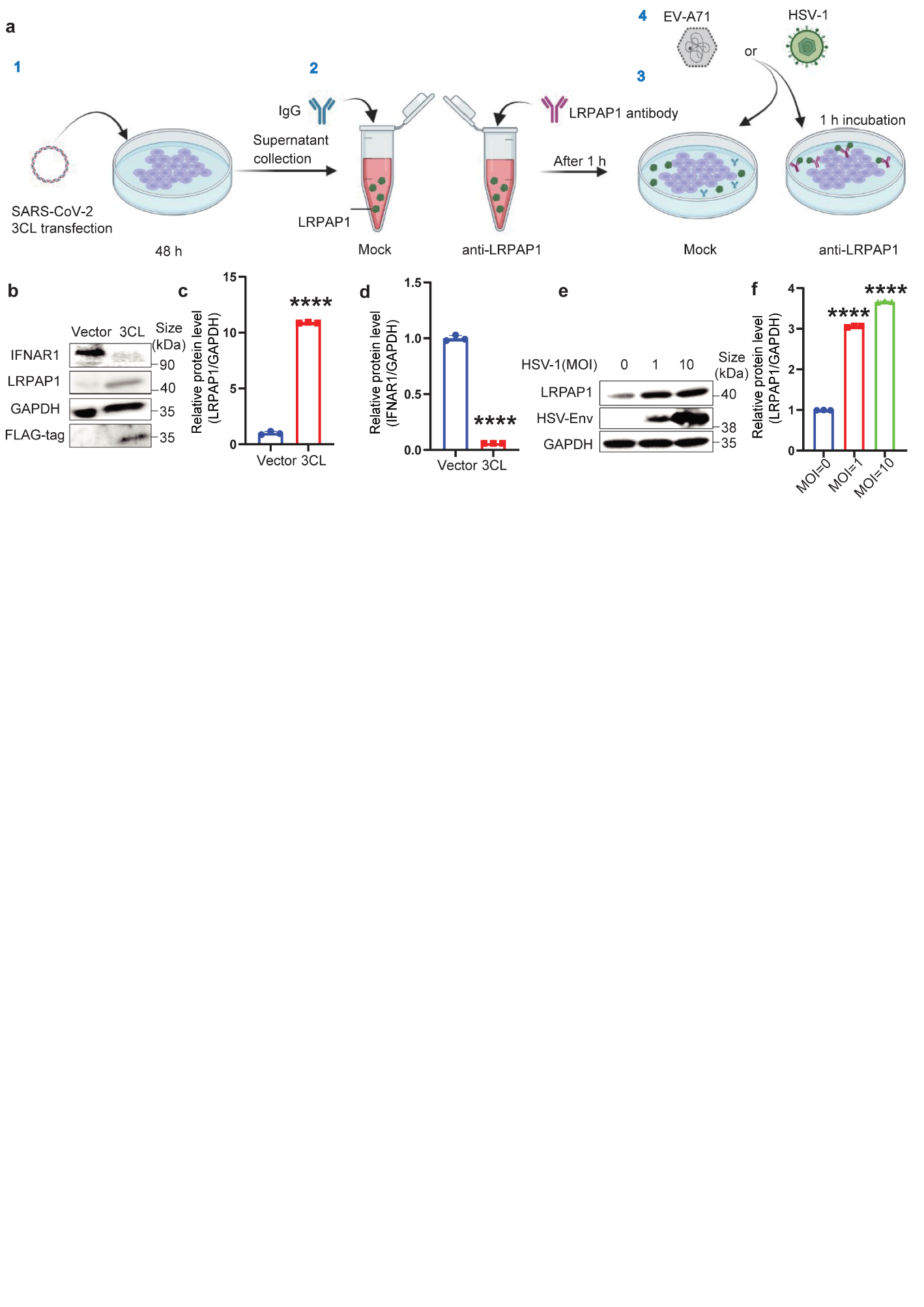
**Fig. S5. The strategy of evading the host cell’s innate immunity via extracellular LRPAP1 is conserved across viruses.** **a,** the schematic diagram for the effect of extracellular LRPAP1 caused by 3CL^pro^ transfection on EV-A71 and HSV-1 infection. Created with BioRender.com. **b,** Immunoblots of lysates in HEK-293T cells transfected with pCMV (Vector) or pCMV-Flag-3CL. **c,** Densitometric analysis of LRPAP1 levels in Fig. S5b was quantified and normalized with GAPDH using ImageJ. **d,** Densitometric analysis of IFNAR1 levels in Fig. S5b was quantified and normalized with GAPDH using ImageJ. **e,** immunoblots of lysates in RD cells infected with HSV-1 at the indicated MOI for 24 h. **f,** Densitometric analysis of LRPAP1 levels in Fig. S5e was quantified and normalized with GAPDH using ImageJ. Results were expressed as mean ± standard deviation (error bars) of at least three repeats. *P ≤ 0.05, **P ≤ 0.01, ***P ≤ 0.001, ****P ≤ 0.0001 (unpaired t-test).

**Fig. S6**

**
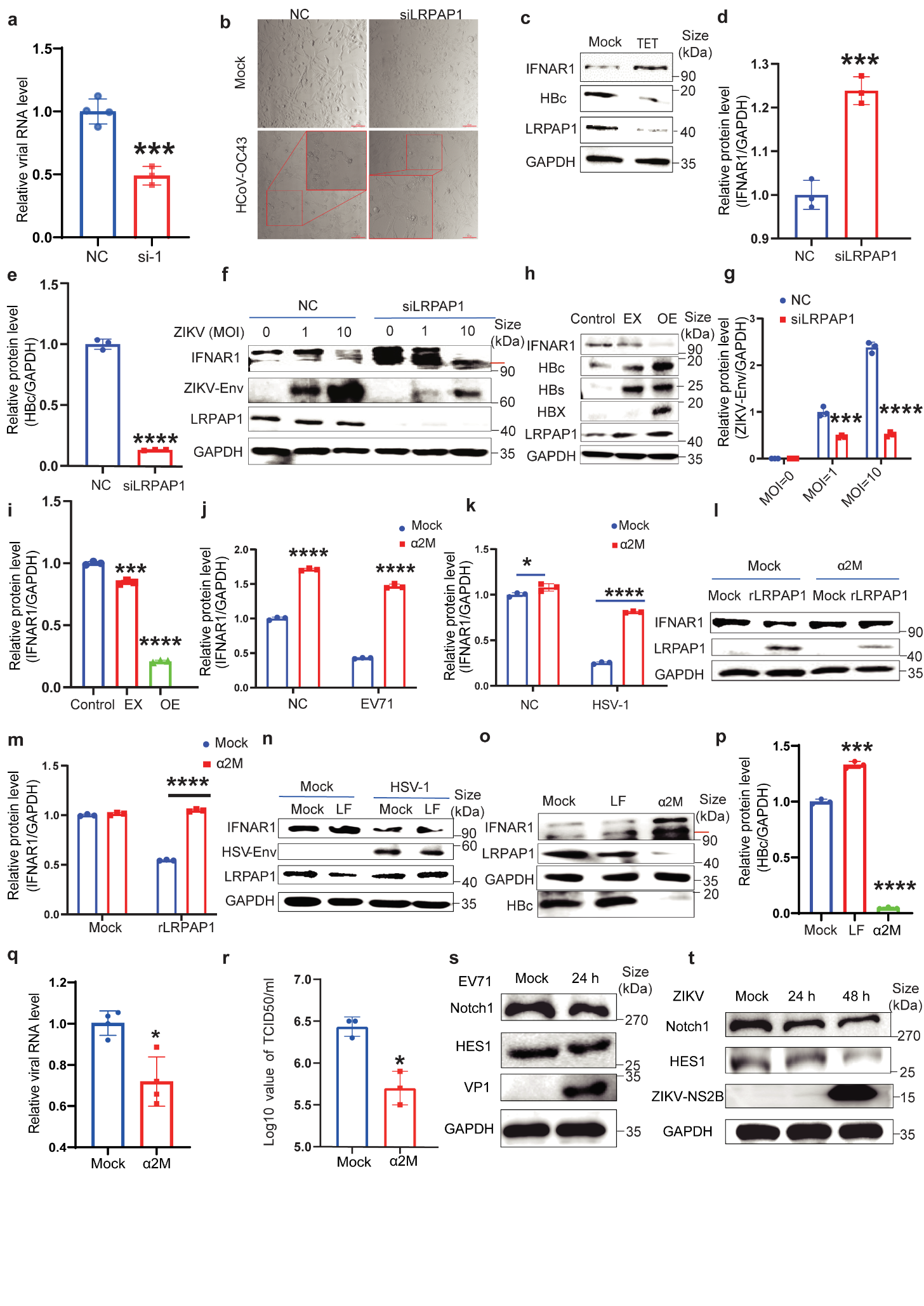
**

**Fig. S6. The inhibition of extracellular LRPAP1 serves as a potent pan-antiviral approach.**

**a,** The effect of siLRPAP1 on the infection of HCoV-OC43. RD cells were transfected with scramble siRNA or siLRPAP1 for 24 h. Then the cells were infected with HCoV-OC43 at an MOI of 10 for 48 h. Viral RNA level of HCoV-OC43 was analysed by RT-qPCR. **b,** RD cells were transfected with scramble siRNA or siLRPAP1 for 24 h, and cells were then inoculated with HCoV-OC43 at an MOI of 1 for 72 h. The CPE of HCoV-OC43 was identified by the vacuoles in the cytoplasm (scale bar = 50 μm). **c,** Immunoblots of HepAD38 for proving the induction of HBV. Tetracycline (TET) was used for inhibiting the integrated HBV genome transcription. **d,** Densitometric analysis of IFNAR1 levels in Fig. 6d was quantified and normalized with GAPDH using ImageJ. **e,** Densitometric analysis of HBc levels in Fig. 6d was quantified and normalized with GAPDH using ImageJ. **f,** Immunoblots of RD cells infected with ZIKV. RD cells were transfected with scramble siRNA or siLRPAP1 for 24 h, and cells were then inoculated with ZIKV at the indicated MOI for 48 h. Cell lysates were harvested and subjected to immunoblotting with anti-IFNAR1, anti-LRPAP1 and ZIKA-Env. The main band was indicated by the red line. **g,** Densitometric analysis of ZIKV Envelope levels in Fig. S6f was quantified and normalized with GAPDH using ImageJ. **h,** Immunoblots of lysates in HepAD38 cells transfected with pcDNA-LRPAP1(OE) for 48h or treated with rLRPAP1 (EX) for 24 h. **i,** Densitometric analysis of IFNAR1 levels in Fig. S6h was quantified and normalized with GAPDH using ImageJ. **j,** Densitometric analysis of IFNAR1 levels in Fig. 6i was quantified and normalized with GAPDH using ImageJ. **k,** Densitometric analysis of IFNAR1 levels in Fig. 6k was quantified and normalized with GAPDH using ImageJ. **l,** Immunoblots of lysates in HEK-293T cells treated with rLRPAP1 (200 nM), α2M (20 nM), or the combination of two proteins, respectively, for 1 h. **m,** Densitometric analysis of IFNAR1 levels in Fig. S6l was quantified and normalized with GAPDH using ImageJ. **n,** The effect of lactoferrin (LF) on HSV-1 infection. RD cells were infected with HSV-1 for 6 h at an MOI of 10. Immunoblots of lysates in RD cells treated with 400 nM LF for 18 h.  **o,** Immunoblots of lysates in HepAD38 cells treated with LF (400 nM) or α2M (20 nM) for 24 h. The main band was indicated by the red line. **p,** Densitometric analysis of HBc levels in Fig. S6o was quantified and normalized with GAPDH using ImageJ. **q, r,** The effect of α2M on the infection of HCoV-OC43. RD cells were pre-treated with or without α2M (20 nM) for 1 h. Then cells were infected with HCoV-OC43 at an MOI of 1 for 72 h. The viral RNA level of HCoV-OC43 (**q**) was analysed by RT-qPCR, and viral titer of HCoV-OC43 was measured by TCID_50_ assay (**r**). **s, t,** Immunoblots of lysates in RD cells infected with either EV71 or ZIKV at MOI=1 for the indicated time. Results were expressed as mean ± standard deviation (error bars) of at least three repeats. *P ≤ 0.05, **P ≤ 0.01, ***P ≤ 0.001, ****P ≤ 0.0001 (unpaired t-test).

**Table S1. Proteins identified by Mass spectrum.**

| Spots No. | Protein Name | Abbreviation | NCBI accession | Theoretical molecular mass (Da) | Protein Core | Masses matched/searched | Sequence coverage |
| --- | --- | --- | --- | --- | --- | --- | --- |
| 34 | Transcription initiation Factor11A subunit1 | TFIID | NP_056943 | 41545 | 58 | 1/1 | 2% |
| 19 | Microtubule-actin cross-linking factor1, isoform 1 | MACF1 | NP_036222.3 | 623626 | 76 | 14/66 | 6% |
| 21 | Talin-2 | TLN2 | NP_055874 | 273781 | 66 | 10/27 | 6% |
| 36 | 60S acidic ribosomal protein L5 | RL5 | NP_000960.2 | 34569 | 94 | 13/51 | 7% |
| 22 | Condensin-2 complex subunit D3 | CNDD3 | NP_056076.1 | 170950 | 59 | 11/24 | 9% |
| 37 | 60S acidic ribosomal protein P2 | RLA2 | NP_000995 | 11658 | 69 | 1/1 | 13% |
| 30 | Zinc finger protein 70 | ZNF70 | NP_068735.1 | 52480 | 61 | 27/40 | 15% |
| 32 | Zinc finger protein 70 | ZNF70 | NP_068735.1 | 50802 | 70 | 8/42 | 15% |
| 26 | Lamin isoform A | LMNA | NP_733821 | 74380 | 58 | 15/74 | 21% |
| 31 | Zinc finger protein 101 | ZN101 | NP_149981.2 | 51902 | 62 | 8/29 | 22% |
| 33 | Elongation factor Tu | EFTU | NP_001393 | 49852 | 87 | 7/47 | 24% |
| 35 | Receptor associated protein | RAP | NP_002328.1 | 41441 | 63 | 8/40 | 24% |
| 27 | Zinc finger protein 37 homolog | ZFP37 | NP_001269444.1 | 73102 | 62 | 11/66 | 26% |
| 13 | 60S acidic ribosomal protein P0 | RLA0 | NP_000993 | 11658 | 47 | 8/44 | 26% |
| 28 | Heat shock cognate 71kDa protein* | HSPA8 | NP_006588.1 | 70294 | 116 | 13/32 | 27% |
| 29 | Heterogeneous nuclear ribonucleoprotein L | HNRPL | NP_001524.2 | 64720 | 102 | 14/66 | 33% |

* Dong Q, Men R, Dan X, Chen Y, Li H, Chen G, Zee B, Wang MHT, He ML. Hsc70 regulates the IRES activity and serves as an antiviral target of enterovirus A71 infection.

*Antiviral Res.* 2018 Feb;150:39-46.

**Table S2. Potential docking site for IFNAR1 in LRPAP1**

| •>2P03_1 _LRPAP1 | | | | | | |
| --- | --- | --- | --- | --- | --- | --- |
| Residue-Number | Residue-Type | Final-Predictions | protein-binding-propensity | ligand-binding-propensity | protein-binding-residues |  |
| •1 | Y | 0.7256 | 0.0518 | 0.3306 | 1 |  |
| •2 | S | 0.8219 | 0.0305 | 0.2329 | 1 |  |
| •3 | R | 0.742 | 0.0725 | 0.3809 | 1 |  |
| •4 | E | 0.8013 | 0.0611 | 0.321 | 1 |  |
| •5 | K | 0.7322 | 0.0427 | 0.3134 | 1 |  |
| •6 | N | 0.7425 | 0.0794 | 0.269 | 1 |  |
| •7 | Q | 0.6999 | 0.056 | 0.3259 | 1 |  |
| •8 | P | 0.7861 | 0.0254 | 0.2831 | 1 |  |
| •9 | K | 0.6272 | 0.0367 | 0.3105 | 1 |  |
| •10 | P | 0.5773 | 0.0238 | 0.2846 | 1 |  |
| •11 | S | 0.5684 | 0.0359 | 0.2317 | 1 |  |
| •12 | P | 0.5634 | 0.0214 | 0.2537 | 1 |  |
| •13 | k | 0.5829 | 0.0268 | 0.1824 | 0 |  |
| •14 | R | 0.4529 | 0.0101 | 0.2505 | 1 |  |
| •15 | e | 0.5014 | 0.0079 | 0.2139 | 0 |  |
| •16 | s | 0.5148 | 0.0064 | 0.1432 | 0 |  |
| •17 | g | 0.3858 | 0.0038 | 0.1254 | 0 |  |
| •18 | e | 0.3288 | 0.0062 | 0.1581 | 0 |  |
| •19 | e | 0.3876 | 0.0135 | 0.1443 | 0 |  |
| •20 | f | 0.454 | 0.0334 | 0.0893 | 0 |  |
| •21 | r | 0.4294 | 0.0273 | 0.1222 | 0 |  |
| •22 | m | 0.4036 | 0.0082 | 0.1886 | 0 |  |
| •23 | e | 0.5125 | 0.0079 | 0.1213 | 0 |  |
| •24 | k | 0.5314 | 0.0252 | 0.0888 | 0 |  |
| •25 | l | 0.4043 | 0.0177 | 0.0839 | 0 |  |
| •26 | n | 0.432 | 0.0215 | 0.1024 | 0 |  |
| •27 | q | 0.4587 | 0.008 | 0.1054 | 0 |  |
| •28 | l | 0.5209 | 0.0136 | 0.0768 | 0 |  |
| •29 | w | 0.4366 | 0.0166 | 0.1221 | 0 |  |
| •30 | e | 0.471 | 0.0075 | 0.123 | 0 |  |
| •31 | k | 0.5476 | 0.0311 | 0.1125 | 0 |  |
| •32 | a | 0.4522 | 0.0208 | 0.0891 | 0 |  |
| •33 | q | 0.5547 | 0.0079 | 0.1503 | 0 |  |
| •34 | R | 0.5647 | 0.009 | 0.2746 | 1 |  |
| •35 | l | 0.4701 | 0.0028 | 0.1769 | 0 |  |
| •36 | H | 0.5405 | 0.0179 | 0.2593 | 1 |  |
| •37 | l | 0.4237 | 0.0093 | 0.1649 | 0 |  |
| •38 | p | 0.4118 | 0.0065 | 0.1699 | 0 |  |
| •39 | p | 0.4293 | 0.0037 | 0.2227 | 0 |  |
| •40 | v | 0.4672 | 0.0035 | 0.2208 | 0 |  |
| •41 | R | 0.4432 | 0.0172 | 0.2735 | 1 |  |
| •42 | l | 0.3568 | 0.019 | 0.1321 | 0 |  |
| •43 | a | 0.3668 | 0.0065 | 0.1464 | 0 |  |
| •44 | e | 0.3826 | 0.0114 | 0.2147 | 0 |  |
| •45 | l | 0.4272 | 0.0244 | 0.1046 | 0 |  |
| •46 | h | 0.3748 | 0.0268 | 0.1569 | 0 |  |
| •47 | a | 0.3428 | 0.0054 | 0.1111 | 0 |  |
| •48 | d | 0.4127 | 0.0415 | 0.1128 | 0 |  |
| •49 | l | 0.4728 | 0.0476 | 0.0978 | 0 |  |
| •50 | k | 0.5495 | 0.0365 | 0.1267 | 0 |  |
| •51 | i | 0.4738 | 0.019 | 0.1525 | 0 |  |
| •52 | q | 0.4464 | 0.0392 | 0.1466 | 0 |  |
| •53 | e | 0.5488 | 0.0487 | 0.1173 | 0 |  |
| •54 | r | 0.4995 | 0.0351 | 0.2032 | 0 |  |
| •55 | d | 0.5052 | 0.02 | 0.1588 | 0 |  |
| •56 | e | 0.541 | 0.0253 | 0.1391 | 0 |  |
| •57 | l | 0.5536 | 0.0066 | 0.1456 | 0 |  |
| •58 | a | 0.5051 | 0.0068 | 0.1153 | 0 |  |
| •59 | w | 0.4556 | 0.0251 | 0.1683 | 0 |  |
| •60 | k | 0.4657 | 0.027 | 0.1089 | 0 |  |
| •61 | k | 0.4862 | 0.0095 | 0.1287 | 0 |  |
| •62 | l | 0.4589 | 0.0096 | 0.113 | 0 |  |
| •63 | k | 0.4653 | 0.0157 | 0.0994 | 0 |  |
| •64 | l | 0.4137 | 0.0062 | 0.1009 | 0 |  |
| •65 | d | 0.489 | 0.0175 | 0.0865 | 0 |  |
| •66 | g | 0.5373 | 0.0106 | 0.0767 | 0 |  |
| •67 | l | 0.4338 | 0.0094 | 0.0932 | 0 |  |
| •68 | d | 0.4308 | 0.0315 | 0.0852 | 0 |  |
| •69 | e | 0.4312 | 0.0289 | 0.0869 | 0 |  |
| •70 | d | 0.5126 | 0.0336 | 0.07 | 0 |  |
| •71 | g | 0.5021 | 0.0104 | 0.0641 | 0 |  |
| •72 | e | 0.5691 | 0.0166 | 0.0699 | 0 |  |
| •73 | k | 0.565 | 0.0205 | 0.0514 | 0 |  |
| •74 | e | 0.5474 | 0.0135 | 0.0574 | 0 |  |
| •75 | a | 0.6331 | 0.013 | 0.0509 | 0 |  |
| •76 | r | 0.5694 | 0.0147 | 0.0859 | 0 |  |
| •77 | l | 0.5265 | 0.0162 | 0.0578 | 0 |  |
| •78 | i | 0.4678 | 0.0108 | 0.077 | 0 |  |
| •79 | r | 0.367 | 0.0106 | 0.1261 | 0 |  |
| •80 | n | 0.4063 | 0.0137 | 0.0787 | 0 |  |
| •81 | l | 0.3468 | 0.0094 | 0.0739 | 0 |  |
| •82 | n | 0.3634 | 0.0078 | 0.0971 | 0 |  |
| •83 | v | 0.3359 | 0.005 | 0.0898 | 0 |  |
| •84 | i | 0.3812 | 0.0316 | 0.0702 | 0 |  |
| •85 | l | 0.3641 | 0.0363 | 0.0655 | 0 |  |
| •86 | a | 0.367 | 0.0105 | 0.0637 | 0 |  |
| •87 | k | 0.3911 | 0.0465 | 0.0738 | 0 |  |
| •88 | y | 0.3331 | 0.0559 | 0.0726 | 0 |  |
| •89 | g | 0.4903 | 0.0303 | 0.0438 | 0 |  |
| •90 | l | 0.4755 | 0.0471 | 0.0531 | 0 |  |
| •91 | d | 0.509 | 0.0142 | 0.0599 | 0 |  |
| •92 | g | 0.6553 | 0.0111 | 0.0508 | 0 |  |
| •93 | k | 0.5703 | 0.0212 | 0.055 | 0 |  |
| •94 | k | 0.4979 | 0.0221 | 0.0545 | 0 |  |
| •95 | d | 0.5526 | 0.0196 | 0.0673 | 0 |  |
| •96 | a | 0.494 | 0.0067 | 0.0822 | 0 |  |
| •97 | r | 0.4622 | 0.0208 | 0.1646 | 0 |  |
| •98 | q | 0.4868 | 0.0081 | 0.1543 | 0 |  |
| •99 | v | 0.4378 | 0.0048 | 0.1582 | 0 |  |
| •100 | t | 0.3259 | 0.0172 | 0.1602 | 0 |  |
| •101 | s | 0.3023 | 0.0121 | 0.1411 | 0 |  |
| •102 | n | 0.3509 | 0.0271 | 0.199 | 0 |  |
| •103 | s | 0.3585 | 0.0111 | 0.1561 | 0 |  |
| •104 | l | 0.2772 | 0.0075 | 0.1972 | 0 |  |
| •105 | s | 0.3073 | 0.0099 | 0.1522 | 0 |  |
| •106 | g | 0.3155 | 0.0058 | 0.1459 | 0 |  |
| •107 | t | 0.3416 | 0.0131 | 0.1476 | 0 |  |
| •108 | q | 0.2733 | 0.0118 | 0.136 | 0 |  |
| •109 | e | 0.3672 | 0.0117 | 0.1197 | 0 |  |
| •110 | d | 0.4494 | 0.009 | 0.1023 | 0 |  |
| •111 | g | 0.5372 | 0.008 | 0.0861 | 0 |  |
| •112 | l | 0.4676 | 0.0356 | 0.0753 | 0 |  |
| •113 | d | 0.5421 | 0.015 | 0.0704 | 0 |  |
| •114 | d | 0.5366 | 0.0273 | 0.0575 | 0 |  |
| •115 | p | 0.7479 | 0.0083 | 0.0601 | 0 |  |
| •116 | r | 0.8802 | 0.0247 | 0.0881 | 0 |  |
| •117 | l | 0.7772 | 0.0115 | 0.0555 | 0 |  |
| •118 | e | 0.7896 | 0.0102 | 0.0747 | 0 |  |
| •119 | k | 0.7757 | 0.0193 | 0.0656 | 0 |  |
| •120 | l | 0.7255 | 0.0501 | 0.0637 | 0 |  |
| •121 | w | 0.7623 | 0.0502 | 0.0912 | 0 |  |
| •122 | h | 0.7628 | 0.0211 | 0.1267 | 0 |  |
| •123 | k | 0.7407 | 0.0293 | 0.1027 | 0 |  |
| •124 | a | 0.7363 | 0.0569 | 0.0693 | 0 |  |
| •125 | k | 0.6809 | 0.0335 | 0.1229 | 0 |  |
| •126 | t | 0.5586 | 0.0334 | 0.1724 | 0 |  |
| •127 | s | 0.5642 | 0.0596 | 0.1265 | 0 |  |
| •128 | g | 0.5762 | 0.0308 | 0.0984 | 0 |  |
| •129 | k | 0.5911 | 0.0781 | 0.1518 | 0 |  |
| •130 | f | 0.5691 | 0.1382 | 0.1162 | 0 |  |
| •131 | s | 0.6111 | 0.0477 | 0.0841 | 0 |  |
| •132 | g | 0.5841 | 0.0084 | 0.1021 | 0 |  |
| •133 | e | 0.5824 | 0.0208 | 0.1128 | 0 |  |
| •134 | e | 0.6471 | 0.0516 | 0.0649 | 0 |  |
| •135 | l | 0.6119 | 0.0331 | 0.0588 | 0 |  |
| •136 | d | 0.655 | 0.0097 | 0.0872 | 0 |  |
| •137 | k | 0.6327 | 0.0099 | 0.082 | 0 |  |
| •138 | l | 0.5692 | 0.0231 | 0.0658 | 0 |  |
| •139 | w | 0.5767 | 0.0181 | 0.1007 | 0 |  |
| •140 | r | 0.4691 | 0.0144 | 0.1276 | 0 |  |
| •141 | e | 0.6119 | 0.0629 | 0.0749 | 0 |  |
| •142 | f | 0.6152 | 0.042 | 0.0696 | 0 |  |
| •143 | l | 0.5521 | 0.0089 | 0.09 | 0 |  |
| •144 | h | 0.6697 | 0.0649 | 0.0845 | 0 |  |
| •145 | h | 0.6599 | 0.0756 | 0.0911 | 0 |  |
| •146 | k | 0.528 | 0.0359 | 0.0879 | 0 |  |
| •147 | e | 0.6077 | 0.0195 | 0.0999 | 0 |  |
| •148 | k | 0.6332 | 0.0424 | 0.0942 | 0 |  |
| •149 | v | 0.5972 | 0.0116 | 0.0867 | 0 |  |
| •150 | h | 0.5785 | 0.018 | 0.1707 | 0 |  |
| •151 | e | 0.5583 | 0.0173 | 0.1208 | 0 |  |
| •152 | y | 0.4972 | 0.017 | 0.1322 | 0 |  |
| •153 | n | 0.5182 | 0.0061 | 0.1649 | 0 |  |
| •154 | v | 0.4078 | 0.0037 | 0.1751 | 0 |  |
| •155 | l | 0.3971 | 0.0135 | 0.1527 | 0 |  |
| •156 | l | 0.3496 | 0.0079 | 0.1592 | 0 |  |
| •157 | e | 0.4266 | 0.0064 | 0.1966 | 0 |  |
| •158 | t | 0.4484 | 0.0057 | 0.1581 | 0 |  |
| •159 | l | 0.3568 | 0.0044 | 0.1462 | 0 |  |
| •160 | s | 0.4398 | 0.0051 | 0.1266 | 0 |  |
| •161 | r | 0.4329 | 0.0138 | 0.1879 | 0 |  |
| •162 | t | 0.3917 | 0.0061 | 0.1147 | 0 |  |
| •163 | e | 0.441 | 0.0073 | 0.1424 | 0 |  |
| •164 | e | 0.5035 | 0.0087 | 0.1317 | 0 |  |
| •165 | i | 0.5704 | 0.0051 | 0.1008 | 0 |  |
| •166 | h | 0.4198 | 0.0183 | 0.1338 | 0 |  |
| •167 | e | 0.4171 | 0.0223 | 0.1279 | 0 |  |
| •168 | n | 0.5407 | 0.0332 | 0.1117 | 0 |  |
| •169 | v | 0.4914 | 0.0135 | 0.1157 | 0 |  |
| •170 | i | 0.4514 | 0.0251 | 0.1672 | 0 |  |
| •171 | s | 0.4403 | 0.0151 | 0.1327 | 0 |  |
| •172 | p | 0.3821 | 0.0132 | 0.1487 | 0 |  |
| •173 | s | 0.398 | 0.0233 | 0.1402 | 0 |  |
| •174 | d | 0.4796 | 0.0162 | 0.1508 | 0 |  |
| •175 | l | 0.3715 | 0.0077 | 0.1704 | 0 |  |
| •176 | s | 0.3594 | 0.0123 | 0.1484 | 0 |  |
| •177 | d | 0.4113 | 0.0125 | 0.1642 | 0 |  |
| •178 | i | 0.351 | 0.0076 | 0.1594 | 0 |  |
| •179 | k | 0.3406 | 0.0168 | 0.1918 | 0 |  |
| •180 | g | 0.3586 | 0.0055 | 0.163 | 0 |  |
| •181 | s | 0.4095 | 0.0126 | 0.1891 | 0 |  |
| •182 | v | 0.3956 | 0.0112 | 0.1763 | 0 |  |
| •183 | l | 0.4127 | 0.0151 | 0.1835 | 0 |  |
| •184 | h | 0.4086 | 0.0287 | 0.1799 | 0 |  |
| •185 | s | 0.4356 | 0.0167 | 0.1173 | 0 |  |
| •186 | r | 0.5431 | 0.0716 | 0.1472 | 0 |  |
| •187 | h | 0.5677 | 0.032 | 0.1543 | 0 |  |
| •188 | t | 0.6072 | 0.0099 | 0.1286 | 0 |  |
| •189 | e | 0.6406 | 0.0165 | 0.1077 | 0 |  |
| •190 | l | 0.6395 | 0.0336 | 0.0742 | 0 |  |
| •191 | k | 0.5492 | 0.0369 | 0.088 | 0 |  |
| •192 | e | 0.7374 | 0.0184 | 0.095 | 0 |  |
| •193 | k | 0.733 | 0.0136 | 0.088 | 0 |  |
| •194 | l | 0.6409 | 0.0094 | 0.0866 | 0 |  |
| •195 | r | 0.6115 | 0.0287 | 0.168 | 0 |  |
| •196 | s | 0.66 | 0.0131 | 0.109 | 0 |  |
| •197 | i | 0.5819 | 0.0226 | 0.1284 | 0 |  |
| •198 | n | 0.5334 | 0.0157 | 0.1634 | 0 |  |
| •199 | q | 0.6429 | 0.0141 | 0.1727 | 0 |  |
| •200 | g | 0.7278 | 0.0135 | 0.0904 | 0 |  |
| •201 | l | 0.5955 | 0.0226 | 0.1255 | 0 |  |
| •202 | d | 0.7333 | 0.0237 | 0.1085 | 0 |  |
| •203 | r | 0.6884 | 0.0463 | 0.1489 | 0 |  |
| •204 | l | 0.7448 | 0.0535 | 0.0919 | 0 |  |
| •205 | R | 0.7405 | 0.0205 | 0.2346 | 1 |  |
| •206 | R | 0.7819 | 0.0253 | 0.2388 | 1 |  |
| •207 | v | 0.7504 | 0.017 | 0.1331 | 0 |  |
| •208 | s | 0.6871 | 0.016 | 0.1164 | 0 |  |
| •209 | H | 0.5741 | 0.0392 | 0.2348 | 1 |  |
| •210 | Q | 0.52 | 0.0186 | 0.2474 | 1 |  |
| •211 | g | 0.5134 | 0.0144 | 0.1432 | 0 |  |
| •212 | Y | 0.4536 | 0.0214 | 0.2741 | 1 |  |
| •213 | s | 0.4749 | 0.0122 | 0.1845 | 0 |  |
| •214 | t | 0.4095 | 0.014 | 0.165 | 0 |  |
| •215 | e | 0.46 | 0.0146 | 0.1963 | 0 |  |
| •216 | a | 0.4531 | 0.0059 | 0.1406 | 0 |  |
| •217 | e | 0.5082 | 0.0377 | 0.1725 | 0 |  |
| •218 | f | 0.4745 | 0.0549 | 0.115 | 0 |  |
| •219 | e | 0.5295 | 0.0198 | 0.1403 | 0 |  |
| •220 | e | 0.5644 | 0.0477 | 0.1073 | 0 |  |
| •221 | p | 0.6596 | 0.0212 | 0.105 | 0 |  |
| •222 | r | 0.759 | 0.0624 | 0.1784 | 0 |  |
| •223 | v | 0.7781 | 0.0496 | 0.0785 | 0 |  |
| •224 | i | 0.6678 | 0.013 | 0.1899 | 0 |  |
| •225 | d | 0.7125 | 0.015 | 0.2207 | 0 |  |
| •226 | l | 0.5999 | 0.0645 | 0.1504 | 0 |  |
| •227 | W | 0.5318 | 0.0703 | 0.2466 | 1 |  |
| •228 | d | 0.5379 | 0.0135 | 0.1875 | 0 |  |
| •229 | l | 0.4561 | 0.0102 | 0.2176 | 0 |  |
| •230 | a | 0.3957 | 0.038 | 0.1207 | 0 |  |
| •231 | q | 0.4536 | 0.0228 | 0.1912 | 0 |  |
| •232 | s | 0.4787 | 0.0104 | 0.1685 | 0 |  |
| •233 | a | 0.3457 | 0.0082 | 0.1061 | 0 |  |
| •234 | n | 0.4648 | 0.0369 | 0.1064 | 0 |  |
| •235 | l | 0.4946 | 0.0264 | 0.077 | 0 |  |
| •236 | t | 0.5 | 0.0202 | 0.0774 | 0 |  |
| •237 | d | 0.5359 | 0.0073 | 0.09 | 0 |  |
| •238 | k | 0.5482 | 0.0112 | 0.0706 | 0 |  |
| •239 | e | 0.67 | 0.0332 | 0.0595 | 0 |  |
| •240 | l | 0.5624 | 0.0285 | 0.0543 | 0 |  |
| •241 | e | 0.6433 | 0.0215 | 0.0835 | 0 |  |
| •242 | a | 0.7294 | 0.016 | 0.0491 | 0 |  |
| •243 | f | 0.6973 | 0.0176 | 0.0538 | 0 |  |
| •244 | r | 0.5241 | 0.0184 | 0.0834 | 0 |  |
| •245 | e | 0.6147 | 0.0125 | 0.0683 | 0 |  |
| •246 | e | 0.7078 | 0.053 | 0.0553 | 0 |  |
| •247 | l | 0.5366 | 0.0381 | 0.0556 | 0 |  |
| •248 | k | 0.574 | 0.0117 | 0.0779 | 0 |  |
| •249 | h | 0.7362 | 0.0556 | 0.0806 | 0 |  |
| •250 | f | 0.563 | 0.0285 | 0.0672 | 0 |  |
| •251 | e | 0.5835 | 0.0265 | 0.0765 | 0 |  |
| •252 | a | 0.6664 | 0.0058 | 0.0669 | 0 |  |
| •253 | k | 0.6552 | 0.0234 | 0.087 | 0 |  |
| •254 | i | 0.4959 | 0.0156 | 0.0965 | 0 |  |
| •255 | e | 0.5708 | 0.0266 | 0.1211 | 0 |  |
| •256 | k | 0.6628 | 0.0738 | 0.083 | 0 |  |
| •257 | h | 0.5916 | 0.0681 | 0.114 | 0 |  |
| •258 | n | 0.7607 | 0.0217 | 0.1178 | 0 |  |
| •259 | h | 0.7438 | 0.0554 | 0.1228 | 0 |  |
| •260 | y | 0.6011 | 0.0322 | 0.138 | 0 |  |
| •261 | q | 0.6159 | 0.0244 | 0.1357 | 0 |  |
| •262 | k | 0.4968 | 0.0168 | 0.1153 | 0 |  |
| •263 | q | 0.5378 | 0.029 | 0.1241 | 0 |  |
| •264 | l | 0.4552 | 0.0175 | 0.1114 | 0 |  |
| •265 | e | 0.536 | 0.0173 | 0.1324 | 0 |  |
| •266 | i | 0.5356 | 0.0119 | 0.1199 | 0 |  |
| •267 | a | 0.5497 | 0.0105 | 0.0906 | 0 |  |
| •268 | h | 0.4622 | 0.0256 | 0.1592 | 0 |  |
| •269 | e | 0.4817 | 0.0207 | 0.1339 | 0 |  |
| •270 | k | 0.4845 | 0.0263 | 0.1108 | 0 |  |
| •271 | l | 0.4219 | 0.0127 | 0.1317 | 0 |  |
| •272 | r | 0.4593 | 0.0246 | 0.2067 | 0 |  |
| •273 | h | 0.5612 | 0.0398 | 0.1862 | 0 |  |
| •274 | a | 0.4506 | 0.0084 | 0.1326 | 0 |  |
| •275 | e | 0.4558 | 0.0294 | 0.1654 | 0 |  |
| •276 | s | 0.5099 | 0.0107 | 0.1644 | 0 |  |
| •277 | v | 0.5065 | 0.0116 | 0.205 | 0 |  |
| •278 | g | 0.4682 | 0.0175 | 0.1489 | 0 |  |
| •279 | d | 0.4647 | 0.0357 | 0.1463 | 0 |  |
| •280 | g | 0.5111 | 0.0188 | 0.1347 | 0 |  |
| •281 | e | 0.5804 | 0.0605 | 0.1539 | 0 |  |
| •282 | r | 0.6147 | 0.1085 | 0.1648 | 0 |  |

**Table S3. Potential docking site for LRPAP1 in IFNAR1.**

| >3S98_IFNAR1 | |  |  |  | |  | | |  |  |
| --- | --- | --- | --- | --- | --- | --- | --- | --- | --- | --- |
| Residue-Number | Residue-Type | Final-Predictions | protein-binding-propensity | | ligand-binding-propensity | | protein-binding-residues |  |  |  |
| 1 | a | 0.0333 | 0.0059 | | 0.1094 | | 0 |  |  |  |
| 2 | d | 0.0397 | 0.0034 | | 0.1552 | | 0 |  |  |  |
| 3 | p | 0.0369 | 0.0022 | | 0.1665 | | 0 |  |  |  |
| 4 | l | 0.0424 | 0.0156 | | 0.1309 | | 0 |  |  |  |
| 5 | k | 0.0334 | 0.0243 | | 0.1203 | | 0 |  |  |  |
| 6 | s | 0.0255 | 0.0073 | | 0.1243 | | 0 |  |  |  |
| 7 | p | 0.0226 | 0.0234 | | 0.1175 | | 0 |  |  |  |
| 8 | q | 0.0218 | 0.0186 | | 0.1437 | | 0 |  |  |  |
| 9 | k | 0.0144 | 0.0342 | | 0.0937 | | 0 |  |  |  |
| 10 | v | 0.0081 | 0.0144 | | 0.0775 | | 0 |  |  |  |
| 11 | e | 0.0111 | 0.0226 | | 0.0995 | | 0 |  |  |  |
| 12 | v | 0.0094 | 0.0127 | | 0.0927 | | 0 |  |  |  |
| 13 | d | 0.0092 | 0.0111 | | 0.1014 | | 0 |  |  |  |
| 14 | i | 0.0082 | 0.0467 | | 0.0955 | | 0 |  |  |  |
| 15 | i | 0.0051 | 0.0327 | | 0.0968 | | 0 |  |  |  |
| 16 | d | 0.0052 | 0.1162 | | 0.0968 | | 0 |  |  |  |
| 17 | d | 0.0104 | 0.0481 | | 0.1052 | | 0 |  |  |  |
| 18 | n | 0.0154 | 0.0418 | | 0.1262 | | 0 |  |  |  |
| 19 | f | 0.0204 | 0.0245 | | 0.1344 | | 0 |  |  |  |
| 20 | i | 0.0237 | 0.0349 | | 0.1129 | | 0 |  |  |  |
| 21 | l | 0.0327 | 0.0438 | | 0.1123 | | 0 |  |  |  |
| 22 | r | 0.0272 | 0.0444 | | 0.18 | | 0 |  |  |  |
| 23 | w | 0.027 | 0.1077 | | 0.1409 | | 0 |  |  |  |
| 24 | n | 0.038 | 0.0417 | | 0.1385 | | 0 |  |  |  |
| 25 | r | 0.0325 | 0.0531 | | 0.213 | | 0 |  |  |  |
| 26 | s | 0.0364 | 0.0264 | | 0.1397 | | 0 |  |  |  |
| 27 | d | 0.0406 | 0.0204 | | 0.1915 | | 0 |  |  |  |
| 28 | e | 0.0225 | 0.03 | | 0.1672 | | 0 |  |  |  |
| 29 | s | 0.0241 | 0.0252 | | 0.1416 | | 0 |  |  |  |
| 30 | v | 0.0281 | 0.0163 | | 0.1473 | | 0 |  |  |  |
| 31 | g | 0.0345 | 0.0073 | | 0.1221 | | 0 |  |  |  |
| 32 | n | 0.041 | 0.031 | | 0.1277 | | 0 |  |  |  |
| 33 | v | 0.0262 | 0.0237 | | 0.1051 | | 0 |  |  |  |
| 34 | t | 0.0316 | 0.0531 | | 0.1193 | | 0 |  |  |  |
| 35 | f | 0.0432 | 0.1075 | | 0.1282 | | 0 |  |  |  |
| 36 | s | 0.045 | 0.0597 | | 0.1012 | | 0 |  |  |  |
| 37 | f | 0.0398 | 0.0906 | | 0.1315 | | 0 |  |  |  |
| 38 | d | 0.0422 | 0.0739 | | 0.1134 | | 0 |  |  |  |
| 39 | y | 0.0529 | 0.1295 | | 0.1406 | | 0 |  |  |  |
| 40 | q | 0.0542 | 0.0538 | | 0.1248 | | 0 |  |  |  |
| 41 | k | 0.0555 | 0.0396 | | 0.1524 | | 0 |  |  |  |
| 42 | t | 0.0365 | 0.0317 | | 0.149 | | 0 |  |  |  |
| 43 | g | 0.0337 | 0.0176 | | 0.1265 | | 0 |  |  |  |
| 44 | m | 0.0281 | 0.0227 | | 0.1759 | | 0 |  |  |  |
| 45 | d | 0.0251 | 0.0142 | | 0.1516 | | 0 |  |  |  |
| 46 | n | 0.016 | 0.0169 | | 0.1486 | | 0 |  |  |  |
| 47 | w | 0.0231 | 0.0565 | | 0.1642 | | 0 |  |  |  |
| 48 | i | 0.0207 | 0.0136 | | 0.1127 | | 0 |  |  |  |
| 49 | k | 0.0215 | 0.0182 | | 0.1253 | | 0 |  |  |  |
| 50 | l | 0.0217 | 0.0219 | | 0.1051 | | 0 |  |  |  |
| 51 | s | 0.0212 | 0.0244 | | 0.1191 | | 0 |  |  |  |
| 52 | g | 0.0241 | 0.0296 | | 0.1303 | | 0 |  |  |  |
| 53 | c | 0.026 | 0.3209 | | 0.0961 | | 0 |  |  |  |
| 54 | q | 0.0233 | 0.0784 | | 0.1725 | | 0 |  |  |  |
| 55 | n | 0.0303 | 0.0643 | | 0.1599 | | 0 |  |  |  |
| 56 | i | 0.0341 | 0.0944 | | 0.1284 | | 0 |  |  |  |
| 57 | t | 0.044 | 0.0917 | | 0.1345 | | 0 |  |  |  |
| 58 | s | 0.043 | 0.0317 | | 0.1586 | | 0 |  |  |  |
| 59 | t | 0.0436 | 0.0714 | | 0.1563 | | 0 |  |  |  |
| 60 | k | 0.0376 | 0.0837 | | 0.1869 | | 0 |  |  |  |
| 61 | c | 0.044 | 0.2494 | | 0.132 | | 0 |  |  |  |
| 62 | n | 0.0347 | 0.2081 | | 0.1797 | | 0 |  |  |  |
| 63 | f | 0.0278 | 0.1913 | | 0.201 | | 0 |  |  |  |
| 64 | s | 0.0315 | 0.0853 | | 0.1412 | | 0 |  |  |  |
| 65 | s | 0.0324 | 0.0271 | | 0.1502 | | 0 |  |  |  |
| 66 | l | 0.0332 | 0.0271 | | 0.1817 | | 0 |  |  |  |
| 67 | k | 0.0261 | 0.0224 | | 0.1712 | | 0 |  |  |  |
| 68 | l | 0.0203 | 0.009 | | 0.1779 | | 0 |  |  |  |
| 69 | n | 0.0247 | 0.0131 | | 0.1449 | | 0 |  |  |  |
| 70 | v | 0.0207 | 0.0047 | | 0.1137 | | 0 |  |  |  |
| 71 | y | 0.0195 | 0.01 | | 0.1502 | | 0 |  |  |  |
| 72 | e | 0.0343 | 0.0098 | | 0.1187 | | 0 |  |  |  |
| 73 | e | 0.0416 | 0.0101 | | 0.1151 | | 0 |  |  |  |
| 74 | i | 0.0679 | 0.0292 | | 0.088 | | 0 |  |  |  |
| 75 | k | 0.0557 | 0.0132 | | 0.1157 | | 0 |  |  |  |
| 76 | l | 0.0672 | 0.011 | | 0.1066 | | 0 |  |  |  |
| 77 | r | 0.0555 | 0.0403 | | 0.0923 | | 0 |  |  |  |
| 78 | i | 0.0421 | 0.0244 | | 0.0779 | | 0 |  |  |  |
| 79 | r | 0.0417 | 0.0291 | | 0.1088 | | 0 |  |  |  |
| 80 | a | 0.0509 | 0.0268 | | 0.0841 | | 0 |  |  |  |
| 81 | e | 0.0679 | 0.038 | | 0.1043 | | 0 |  |  |  |
| 82 | k | 0.0483 | 0.0256 | | 0.1047 | | 0 |  |  |  |
| 83 | e | 0.0315 | 0.0302 | | 0.1048 | | 0 |  |  |  |
| 84 | n | 0.0357 | 0.0223 | | 0.1283 | | 0 |  |  |  |
| 85 | t | 0.0169 | 0.0249 | | 0.1192 | | 0 |  |  |  |
| 86 | s | 0.0154 | 0.0506 | | 0.096 | | 0 |  |  |  |
| 87 | s | 0.0153 | 0.0192 | | 0.11 | | 0 |  |  |  |
| 88 | w | 0.0168 | 0.0623 | | 0.1518 | | 0 |  |  |  |
| 89 | y | 0.0116 | 0.0218 | | 0.1472 | | 0 |  |  |  |
| 90 | e | 0.0135 | 0.0204 | | 0.1446 | | 0 |  |  |  |
| 91 | v | 0.0164 | 0.0171 | | 0.11 | | 0 |  |  |  |
| 92 | d | 0.0129 | 0.0181 | | 0.1627 | | 0 |  |  |  |
| 93 | s | 0.0147 | 0.0273 | | 0.1824 | | 0 |  |  |  |
| 94 | f | 0.0136 | 0.1158 | | 0.1735 | | 0 |  |  |  |
| 95 | t | 0.0129 | 0.0223 | | 0.1705 | | 0 |  |  |  |
| 96 | p | 0.0196 | 0.0314 | | 0.153 | | 0 |  |  |  |
| 97 | f | 0.0317 | 0.0186 | | 0.1814 | | 0 |  |  |  |
| 98 | R | 0.0352 | 0.0239 | | 0.2904 | | 1 |  |  |  |
| 99 | k | 0.0334 | 0.0341 | | 0.1894 | | 0 |  |  |  |
| 100 | a | 0.0335 | 0.0353 | | 0.1421 | | 0 |  |  |  |
| 101 | Q | 0.0332 | 0.0342 | | 0.27 | | 1 |  |  |  |
| 102 | i | 0.0231 | 0.0547 | | 0.1629 | | 0 |  |  |  |
| 103 | g | 0.0413 | 0.0499 | | 0.1369 | | 0 |  |  |  |
| 104 | p | 0.0331 | 0.0499 | | 0.1469 | | 0 |  |  |  |
| 105 | p | 0.027 | 0.05 | | 0.138 | | 0 |  |  |  |
| 106 | e | 0.0241 | 0.0231 | | 0.1498 | | 0 |  |  |  |
| 107 | v | 0.0265 | 0.0148 | | 0.0946 | | 0 |  |  |  |
| 108 | h | 0.0199 | 0.0236 | | 0.1255 | | 0 |  |  |  |
| 109 | l | 0.016 | 0.0187 | | 0.1041 | | 0 |  |  |  |
| 110 | e | 0.0134 | 0.0134 | | 0.1114 | | 0 |  |  |  |
| 111 | a | 0.0085 | 0.0092 | | 0.0813 | | 0 |  |  |  |
| 112 | e | 0.0092 | 0.0156 | | 0.0958 | | 0 |  |  |  |
| 113 | d | 0.0138 | 0.011 | | 0.0853 | | 0 |  |  |  |
| 114 | k | 0.0115 | 0.0146 | | 0.086 | | 0 |  |  |  |
| 115 | a | 0.0165 | 0.0066 | | 0.0753 | | 0 |  |  |  |
| 116 | i | 0.0216 | 0.0183 | | 0.0825 | | 0 |  |  |  |
| 117 | v | 0.0258 | 0.0074 | | 0.0798 | | 0 |  |  |  |
| 118 | i | 0.0346 | 0.0207 | | 0.0792 | | 0 |  |  |  |
| 119 | h | 0.0299 | 0.0541 | | 0.0959 | | 0 |  |  |  |
| 120 | i | 0.0264 | 0.0502 | | 0.0902 | | 0 |  |  |  |
| 121 | s | 0.0302 | 0.0431 | | 0.0987 | | 0 |  |  |  |
| 122 | p | 0.0331 | 0.0919 | | 0.1141 | | 0 |  |  |  |
| 123 | g | 0.037 | 0.0215 | | 0.112 | | 0 |  |  |  |
| 124 | t | 0.0258 | 0.0335 | | 0.13 | | 0 |  |  |  |
| 125 | k | 0.01 | 0.0284 | | 0.1195 | | 0 |  |  |  |
| 126 | d | 0.0127 | 0.0172 | | 0.1158 | | 0 |  |  |  |
| 127 | s | 0.0111 | 0.0173 | | 0.1342 | | 0 |  |  |  |
| 128 | v | 0.0074 | 0.0074 | | 0.1362 | | 0 |  |  |  |
| 129 | m | 0.008 | 0.0155 | | 0.1503 | | 0 |  |  |  |
| 130 | w | 0.0091 | 0.0144 | | 0.1794 | | 0 |  |  |  |
| 131 | a | 0.0082 | 0.0053 | | 0.1206 | | 0 |  |  |  |
| 132 | l | 0.0064 | 0.009 | | 0.1776 | | 0 |  |  |  |
| 133 | d | 0.0052 | 0.0274 | | 0.1817 | | 0 |  |  |  |
| 134 | g | 0.0099 | 0.0189 | | 0.1344 | | 0 |  |  |  |
| 135 | l | 0.0164 | 0.0113 | | 0.192 | | 0 |  |  |  |
| 136 | s | 0.017 | 0.0132 | | 0.1293 | | 0 |  |  |  |
| 137 | f | 0.0133 | 0.0213 | | 0.1341 | | 0 |  |  |  |
| 138 | t | 0.0152 | 0.014 | | 0.1099 | | 0 |  |  |  |
| 139 | y | 0.0158 | 0.0778 | | 0.1211 | | 0 |  |  |  |
| 140 | s | 0.0172 | 0.0222 | | 0.1 | | 0 |  |  |  |
| 141 | l | 0.0197 | 0.032 | | 0.113 | | 0 |  |  |  |
| 142 | v | 0.0198 | 0.0106 | | 0.1151 | | 0 |  |  |  |
| 143 | i | 0.021 | 0.0247 | | 0.117 | | 0 |  |  |  |
| 144 | w | 0.0237 | 0.0754 | | 0.121 | | 0 |  |  |  |
| 145 | k | 0.0182 | 0.0553 | | 0.1109 | | 0 |  |  |  |
| 146 | n | 0.0133 | 0.0587 | | 0.147 | | 0 |  |  |  |
| 147 | s | 0.0138 | 0.0642 | | 0.1371 | | 0 |  |  |  |
| 148 | s | 0.0274 | 0.0519 | | 0.1447 | | 0 |  |  |  |
| 149 | g | 0.0339 | 0.0165 | | 0.1541 | | 0 |  |  |  |
| 150 | v | 0.0229 | 0.0136 | | 0.1358 | | 0 |  |  |  |
| 151 | e | 0.0178 | 0.0295 | | 0.1366 | | 0 |  |  |  |
| 152 | e | 0.0262 | 0.0112 | | 0.1372 | | 0 |  |  |  |
| 153 | r | 0.0258 | 0.0132 | | 0.2055 | | 0 |  |  |  |
| 154 | i | 0.0176 | 0.0053 | | 0.1551 | | 0 |  |  |  |
| 155 | e | 0.0206 | 0.0071 | | 0.1901 | | 0 |  |  |  |
| 156 | n | 0.0237 | 0.0095 | | 0.1529 | | 0 |  |  |  |
| 157 | i | 0.0225 | 0.009 | | 0.1533 | | 0 |  |  |  |
| 158 | y | 0.0182 | 0.0176 | | 0.2046 | | 0 |  |  |  |
| 159 | s | 0.0219 | 0.0075 | | 0.1391 | | 0 |  |  |  |
| 160 | r | 0.0262 | 0.0114 | | 0.216 | | 0 |  |  |  |
| 161 | h | 0.0271 | 0.0183 | | 0.1362 | | 0 |  |  |  |
| 162 | k | 0.0187 | 0.018 | | 0.125 | | 0 |  |  |  |
| 163 | i | 0.0222 | 0.0327 | | 0.108 | | 0 |  |  |  |
| 164 | y | 0.0219 | 0.0217 | | 0.1454 | | 0 |  |  |  |
| 165 | k | 0.0255 | 0.0264 | | 0.1234 | | 0 |  |  |  |
| 166 | l | 0.0278 | 0.0474 | | 0.128 | | 0 |  |  |  |
| 167 | s | 0.0267 | 0.017 | | 0.1294 | | 0 |  |  |  |
| 168 | p | 0.0211 | 0.02 | | 0.1347 | | 0 |  |  |  |
| 169 | e | 0.0226 | 0.0179 | | 0.1402 | | 0 |  |  |  |
| 170 | t | 0.0278 | 0.0277 | | 0.0971 | | 0 |  |  |  |
| 171 | t | 0.0239 | 0.0159 | | 0.1138 | | 0 |  |  |  |
| 172 | y | 0.0249 | 0.1021 | | 0.1352 | | 0 |  |  |  |
| 173 | c | 0.0332 | 0.1044 | | 0.0901 | | 0 |  |  |  |
| 174 | l | 0.0195 | 0.0386 | | 0.1161 | | 0 |  |  |  |
| 175 | k | 0.0178 | 0.0579 | | 0.1387 | | 0 |  |  |  |
| 176 | v | 0.0225 | 0.0633 | | 0.1096 | | 0 |  |  |  |
| 177 | k | 0.0178 | 0.0583 | | 0.1415 | | 0 |  |  |  |
| 178 | a | 0.0159 | 0.0225 | | 0.1238 | | 0 |  |  |  |
| 179 | a | 0.0189 | 0.0092 | | 0.1361 | | 0 |  |  |  |
| 180 | l | 0.0214 | 0.014 | | 0.1614 | | 0 |  |  |  |
| 181 | l | 0.013 | 0.0123 | | 0.1465 | | 0 |  |  |  |
| 182 | t | 0.018 | 0.0123 | | 0.1605 | | 0 |  |  |  |
| 183 | s | 0.0094 | 0.0116 | | 0.1474 | | 0 |  |  |  |
| 184 | W | 0.0115 | 0.0359 | | 0.2851 | | 1 |  |  |  |
| 185 | k | 0.0149 | 0.0553 | | 0.1782 | | 0 |  |  |  |
| 186 | i | 0.0155 | 0.0173 | | 0.1974 | | 0 |  |  |  |
| 187 | g | 0.0143 | 0.0402 | | 0.1302 | | 0 |  |  |  |
| 188 | v | 0.0211 | 0.034 | | 0.1858 | | 0 |  |  |  |
| 189 | Y | 0.0225 | 0.179 | | 0.2662 | | 1 |  |  |  |
| 190 | s | 0.0174 | 0.2023 | | 0.1127 | | 0 |  |  |  |
| 191 | p | 0.0169 | 0.0495 | | 0.1786 | | 0 |  |  |  |
| 192 | v | 0.0227 | 0.0443 | | 0.1675 | | 0 |  |  |  |
| 193 | h | 0.0188 | 0.1011 | | 0.2094 | | 0 |  |  |  |
| 194 | c | 0.0317 | 0.2457 | | 0.103 | | 0 |  |  |  |
| 195 | i | 0.0231 | 0.0412 | | 0.1341 | | 0 |  |  |  |
| 196 | k | 0.02 | 0.0432 | | 0.1903 | | 0 |  |  |  |
| 197 | t | 0.0186 | 0.0563 | | 0.1252 | | 0 |  |  |  |
| 198 | t | 0.042 | 0.0305 | | 0.1174 | | 0 |  |  |  |
| 199 | v | 0.0219 | 0.0119 | | 0.1181 | | 0 |  |  |  |
| 200 | e | 0.0241 | 0.0134 | | 0.1466 | | 0 |  |  |  |
| 201 | n | 0.0285 | 0.0106 | | 0.1341 | | 0 |  |  |  |
| 202 | e | 0.0344 | 0.014 | | 0.1366 | | 0 |  |  |  |
| 203 | l | 0.027 | 0.0098 | | 0.1194 | | 0 |  |  |  |
| 204 | p | 0.0264 | 0.0066 | | 0.1334 | | 0 |  |  |  |
| 205 | p | 0.0217 | 0.0075 | | 0.1271 | | 0 |  |  |  |
| 206 | p | 0.0179 | 0.0137 | | 0.1134 | | 0 |  |  |  |
| 207 | e | 0.0273 | 0.0169 | | 0.1192 | | 0 |  |  |  |
| 208 | n | 0.0311 | 0.02 | | 0.1051 | | 0 |  |  |  |
| 209 | i | 0.0361 | 0.012 | | 0.108 | | 0 |  |  |  |
| 210 | e | 0.0326 | 0.0128 | | 0.1147 | | 0 |  |  |  |
| 211 | v | 0.0291 | 0.007 | | 0.1023 | | 0 |  |  |  |
| 212 | s | 0.0263 | 0.0068 | | 0.1078 | | 0 |  |  |  |
| 213 | v | 0.0206 | 0.0186 | | 0.1054 | | 0 |  |  |  |
| 214 | q | 0.0164 | 0.0519 | | 0.1245 | | 0 |  |  |  |
| 215 | n | 0.0155 | 0.0548 | | 0.117 | | 0 |  |  |  |
| 216 | q | 0.0203 | 0.0264 | | 0.165 | | 0 |  |  |  |
| 217 | n | 0.0291 | 0.0196 | | 0.1645 | | 0 |  |  |  |
| 218 | y | 0.0171 | 0.0455 | | 0.1866 | | 0 |  |  |  |
| 219 | v | 0.0182 | 0.014 | | 0.1423 | | 0 |  |  |  |
| 220 | l | 0.0107 | 0.0311 | | 0.1586 | | 0 |  |  |  |
| 221 | k | 0.009 | 0.0215 | | 0.1771 | | 0 |  |  |  |
| 222 | w | 0.0069 | 0.0546 | | 0.2085 | | 0 |  |  |  |
| 223 | d | 0.0101 | 0.0324 | | 0.1593 | | 0 |  |  |  |
| 224 | Y | 0.0059 | 0.0292 | | 0.287 | | 1 |  |  |  |
| 225 | t | 0.0081 | 0.0233 | | 0.1965 | | 0 |  |  |  |
| 226 | Y | 0.0095 | 0.0768 | | 0.2271 | | 1 |  |  |  |
| 227 | a | 0.0141 | 0.0259 | | 0.1597 | | 0 |  |  |  |
| 228 | n | 0.0178 | 0.048 | | 0.2126 | | 0 |  |  |  |
| 229 | m | 0.0175 | 0.0383 | | 0.1837 | | 0 |  |  |  |
| 230 | t | 0.0247 | 0.0702 | | 0.151 | | 0 |  |  |  |
| 231 | f | 0.0254 | 0.1164 | | 0.1387 | | 0 |  |  |  |
| 232 | q | 0.0288 | 0.0663 | | 0.1528 | | 0 |  |  |  |
| 233 | v | 0.0409 | 0.0333 | | 0.1174 | | 0 |  |  |  |
| 234 | q | 0.0356 | 0.0866 | | 0.1231 | | 0 |  |  |  |
| 235 | w | 0.0586 | 0.0771 | | 0.1459 | | 0 |  |  |  |
| 236 | l | 0.0672 | 0.0431 | | 0.0938 | | 0 |  |  |  |
| 237 | h | 0.0676 | 0.0528 | | 0.1299 | | 0 |  |  |  |
| 238 | a | 0.0362 | 0.0162 | | 0.1042 | | 0 |  |  |  |
| 239 | f | 0.0402 | 0.0465 | | 0.1384 | | 0 |  |  |  |
| 240 | l | 0.0312 | 0.0198 | | 0.1145 | | 0 |  |  |  |
| 241 | k | 0.0359 | 0.0363 | | 0.11 | | 0 |  |  |  |
| 242 | r | 0.0347 | 0.0269 | | 0.1565 | | 0 |  |  |  |
| 243 | n | 0.0321 | 0.0142 | | 0.1226 | | 0 |  |  |  |
| 244 | p | 0.0342 | 0.0088 | | 0.1471 | | 0 |  |  |  |
| 245 | g | 0.0395 | 0.0099 | | 0.137 | | 0 |  |  |  |
| 246 | n | 0.0544 | 0.0264 | | 0.1327 | | 0 |  |  |  |
| 247 | h | 0.0468 | 0.0408 | | 0.1482 | | 0 |  |  |  |
| 248 | l | 0.0424 | 0.0208 | | 0.1322 | | 0 |  |  |  |
| 249 | y | 0.0453 | 0.013 | | 0.1753 | | 0 |  |  |  |
| 250 | k | 0.0441 | 0.0386 | | 0.1198 | | 0 |  |  |  |
| 251 | w | 0.0495 | 0.0649 | | 0.1514 | | 0 |  |  |  |
| 252 | k | 0.0451 | 0.0217 | | 0.1392 | | 0 |  |  |  |
| 253 | q | 0.056 | 0.0138 | | 0.1721 | | 0 |  |  |  |
| 254 | i | 0.0468 | 0.0261 | | 0.1191 | | 0 |  |  |  |
| 255 | p | 0.0415 | 0.0145 | | 0.1305 | | 0 |  |  |  |
| 256 | d | 0.0527 | 0.0286 | | 0.1389 | | 0 |  |  |  |
| 257 | c | 0.0505 | 0.0872 | | 0.1104 | | 0 |  |  |  |
| 258 | e | 0.041 | 0.0556 | | 0.1647 | | 0 |  |  |  |
| 259 | n | 0.085 | 0.0458 | | 0.1494 | | 0 |  |  |  |
| 260 | v | 0.0667 | 0.0294 | | 0.1246 | | 0 |  |  |  |
| 261 | k | 0.0495 | 0.0376 | | 0.1504 | | 0 |  |  |  |
| 262 | t | 0.0495 | 0.0164 | | 0.1483 | | 0 |  |  |  |
| 263 | t | 0.0448 | 0.0301 | | 0.1257 | | 0 |  |  |  |
| 264 | q | 0.043 | 0.0292 | | 0.1911 | | 0 |  |  |  |
| 265 | c | 0.058 | 0.1212 | | 0.1263 | | 0 |  |  |  |
| 266 | v | 0.0421 | 0.0488 | | 0.1415 | | 0 |  |  |  |
| 267 | f | 0.0423 | 0.1232 | | 0.1882 | | 0 |  |  |  |
| 268 | p | 0.036 | 0.05 | | 0.1685 | | 0 |  |  |  |
| 269 | q | 0.037 | 0.0493 | | 0.208 | | 0 |  |  |  |
| 270 | n | 0.0334 | 0.031 | | 0.1775 | | 0 |  |  |  |
| 271 | v | 0.03 | 0.0239 | | 0.1214 | | 0 |  |  |  |
| 272 | f | 0.0319 | 0.0265 | | 0.1555 | | 0 |  |  |  |
| 273 | q | 0.0281 | 0.0175 | | 0.1468 | | 0 |  |  |  |
| 274 | k | 0.0229 | 0.0135 | | 0.1255 | | 0 |  |  |  |
| 275 | g | 0.0324 | 0.0307 | | 0.0927 | | 0 |  |  |  |
| 276 | i | 0.0255 | 0.0239 | | 0.0923 | | 0 |  |  |  |
| 277 | y | 0.0492 | 0.0717 | | 0.1039 | | 0 |  |  |  |
| 278 | l | 0.0404 | 0.0161 | | 0.0831 | | 0 |  |  |  |
| 279 | l | 0.0467 | 0.0371 | | 0.0718 | | 0 |  |  |  |
| 280 | r | 0.0377 | 0.132 | | 0.0845 | | 0 |  |  |  |
| 281 | v | 0.0357 | 0.0667 | | 0.0764 | | 0 |  |  |  |
| 282 | q | 0.0501 | 0.0952 | | 0.0965 | | 0 |  |  |  |
| 283 | a | 0.046 | 0.0939 | | 0.0767 | | 0 |  |  |  |
| 284 | s | 0.0866 | 0.0444 | | 0.1113 | | 0 |  |  |  |
| 285 | d | 0.1037 | 0.0479 | | 0.1543 | | 0 |  |  |  |
| 286 | g | 0.052 | 0.0445 | | 0.104 | | 0 |  |  |  |
| 287 | n | 0.0609 | 0.0663 | | 0.1603 | | 0 |  |  |  |
| 288 | n | 0.0414 | 0.0637 | | 0.1625 | | 0 |  |  |  |
| 289 | t | 0.0389 | 0.0445 | | 0.1434 | | 0 |  |  |  |
| 290 | s | 0.0475 | 0.1092 | | 0.1103 | | 0 |  |  |  |
| 291 | f | 0.0467 | 0.0556 | | 0.2199 | | 0 |  |  |  |
| 292 | w | 0.048 | 0.1299 | | 0.2206 | | 0 |  |  |  |
| 293 | s | 0.0415 | 0.0746 | | 0.1131 | | 0 |  |  |  |
| 294 | e | 0.0441 | 0.049 | | 0.1706 | | 0 |  |  |  |
| 295 | e | 0.0555 | 0.0688 | | 0.1686 | | 0 |  |  |  |
| 296 | i | 0.0461 | 0.0213 | | 0.1393 | | 0 |  |  |  |
| 297 | k | 0.0477 | 0.0319 | | 0.1406 | | 0 |  |  |  |
| 298 | f | 0.0391 | 0.0662 | | 0.1618 | | 0 |  |  |  |
| 299 | d | 0.0353 | 0.0351 | | 0.1563 | | 0 |  |  |  |
| 300 | t | 0.0392 | 0.0485 | | 0.1571 | | 0 |  |  |  |
| 301 | e | 0.048 | 0.0202 | | 0.1818 | | 0 |  |  |  |
| 302 | i | 0.0419 | 0.015 | | 0.1581 | | 0 |  |  |  |
| 303 | q | 0.0337 | 0.0125 | | 0.2023 | | 0 |  |  |  |
| 304 | a | 0.0358 | 0.0095 | | 0.1575 | | 0 |  |  |  |
| 305 | F | 0.0265 | 0.0075 | | 0.2989 | | 1 |  |  |  |
| 306 | l | 0.0193 | 0.0085 | | 0.1913 | | 0 |  |  |  |

**Movie S1.**

Mice with EV71 injection

Movie S2.

Mice with EV71 and rLRPAP1 injection

Movie S3.

Live-cell images with PBS

Movie S4.

Live-cell images with rLRPAP1

Original films of western blot


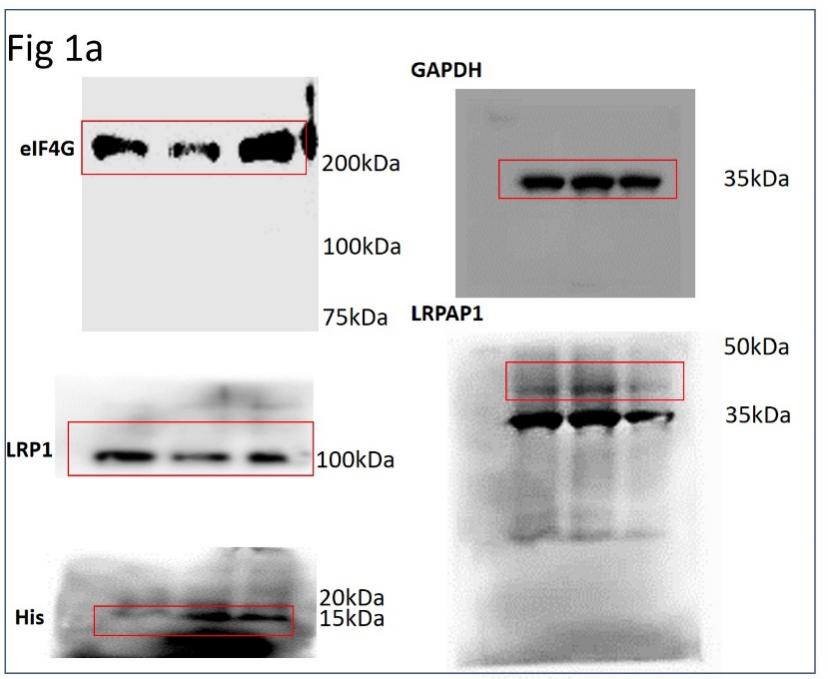


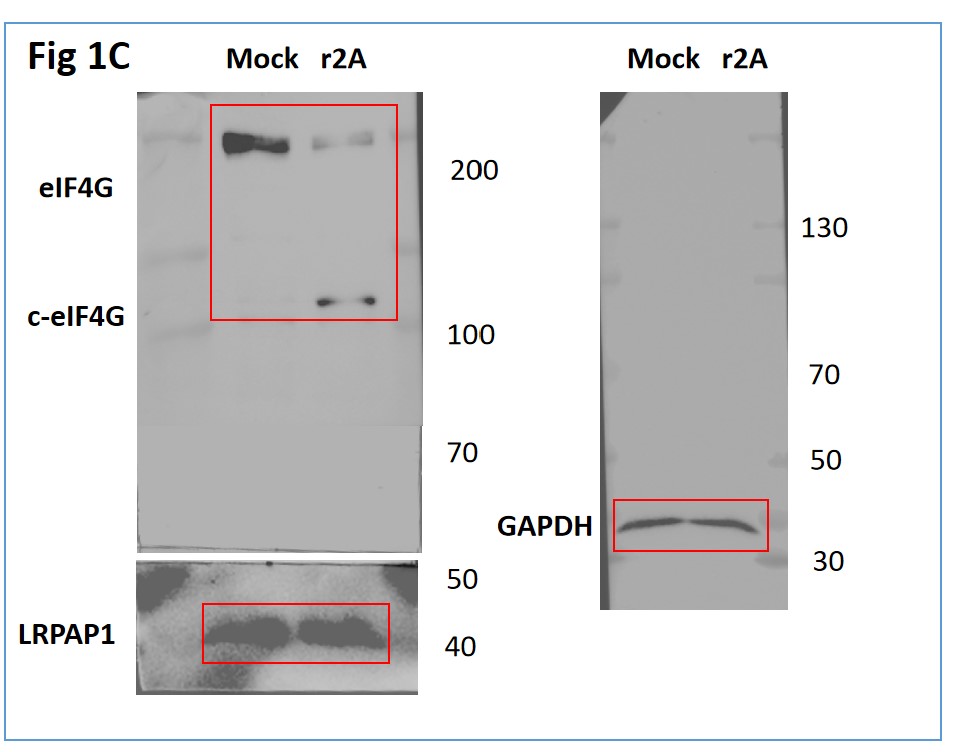

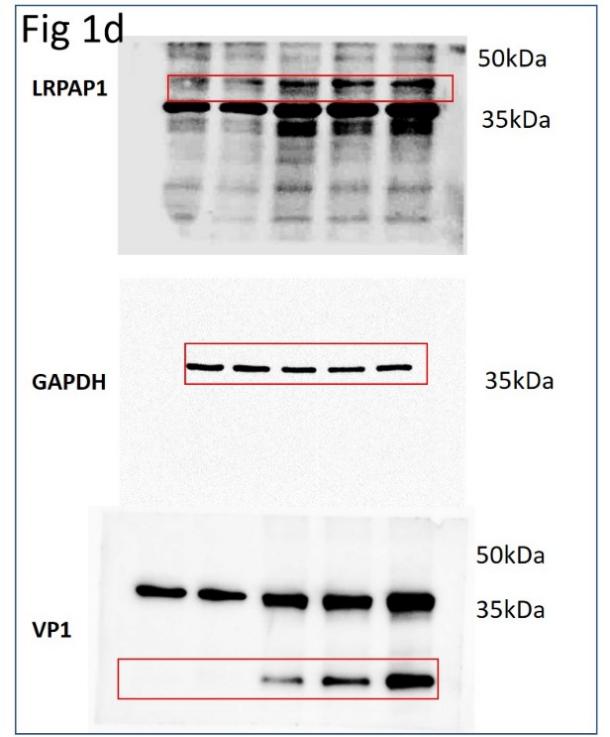

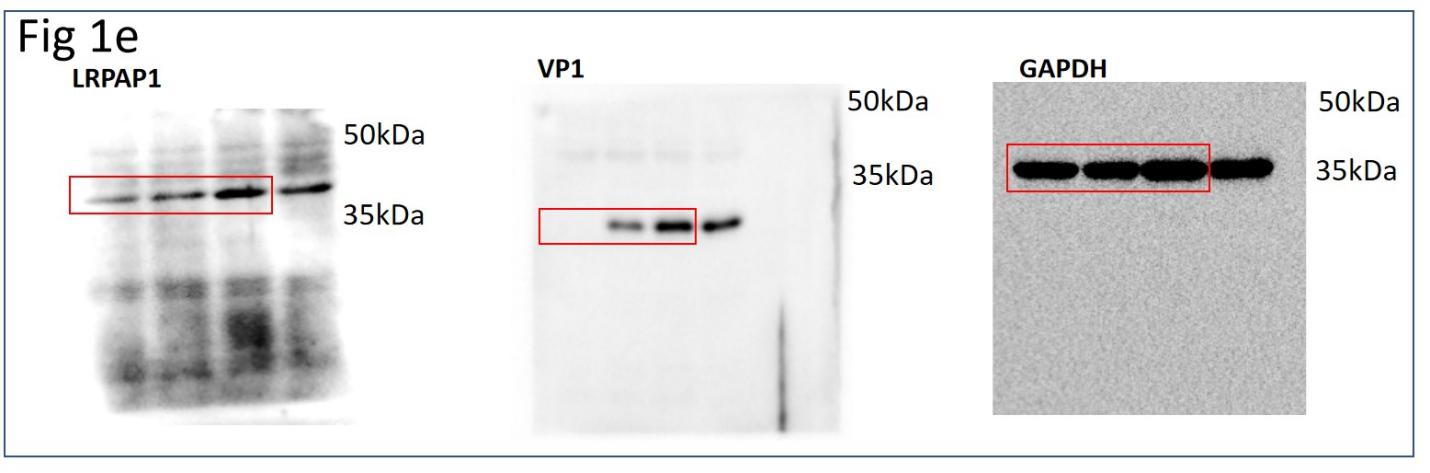


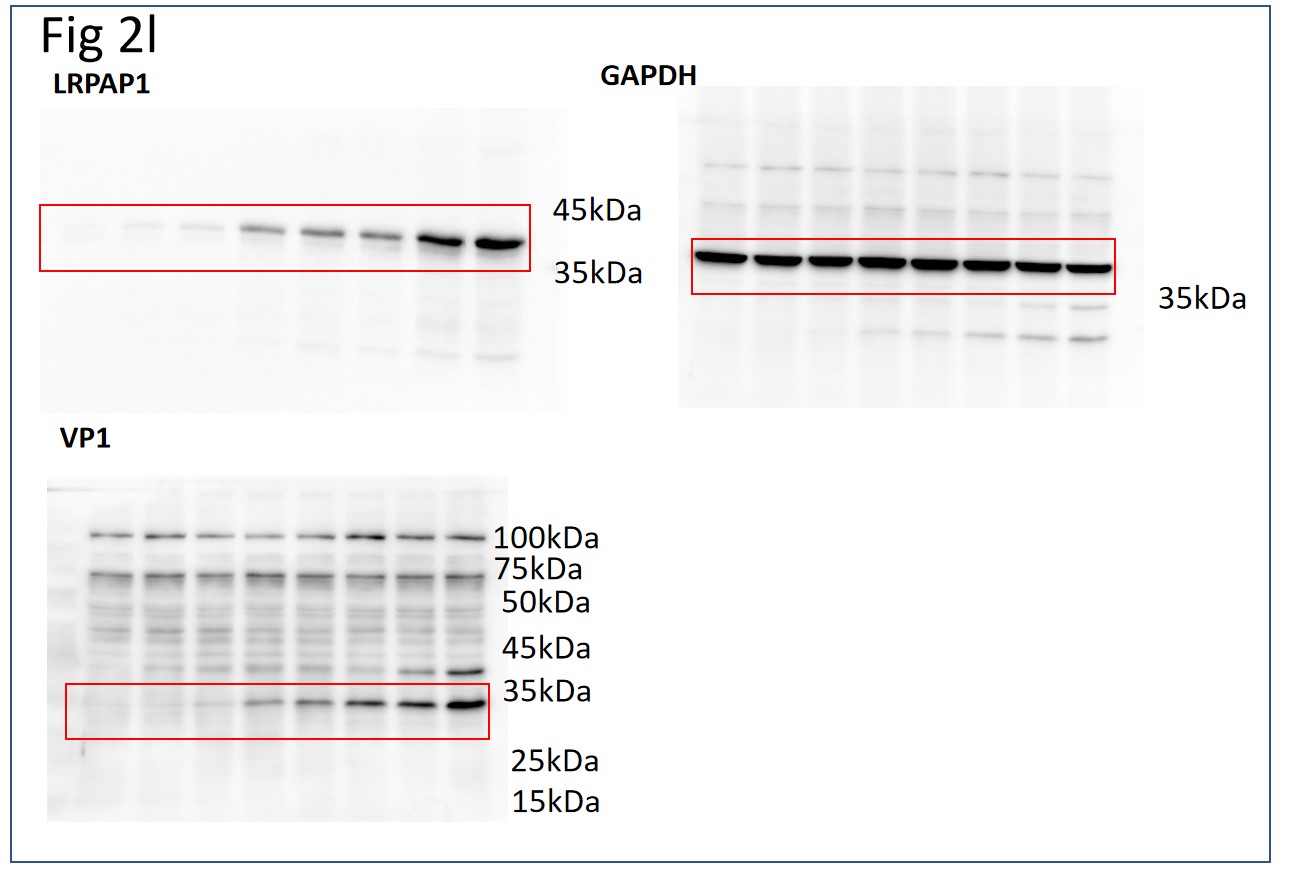


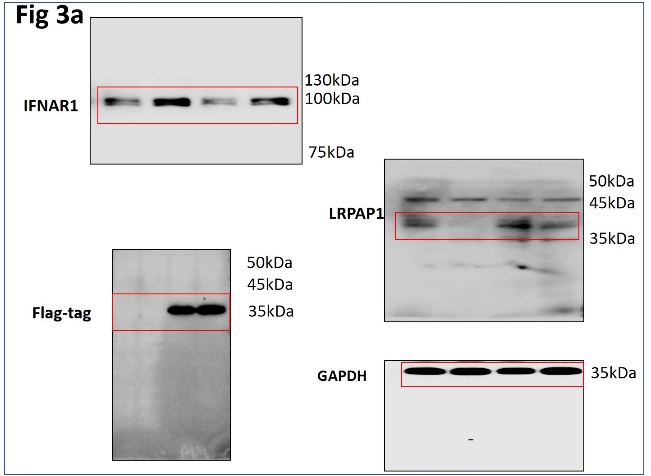

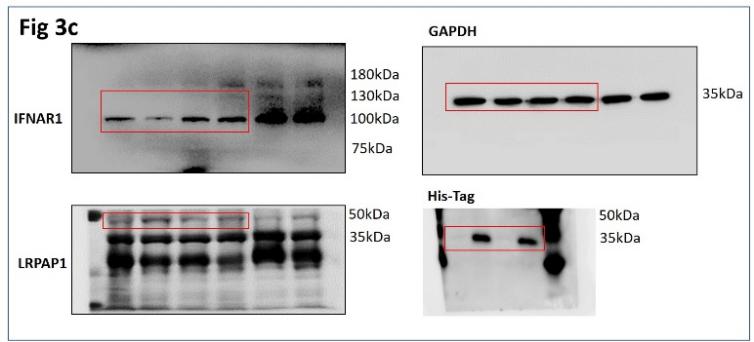


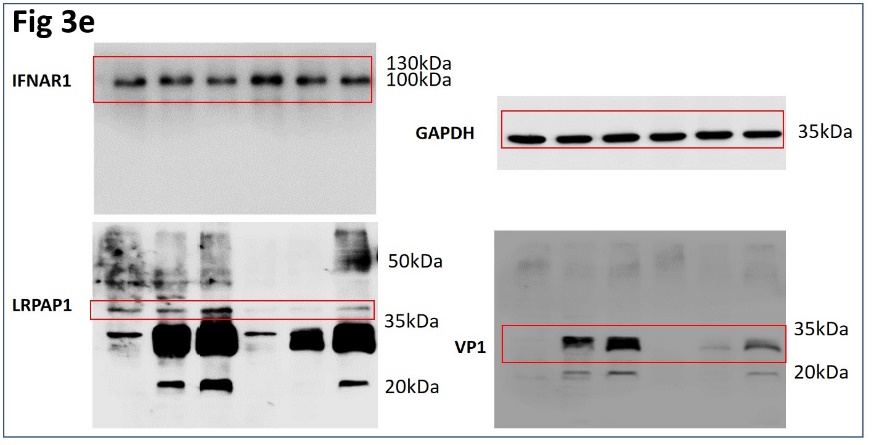

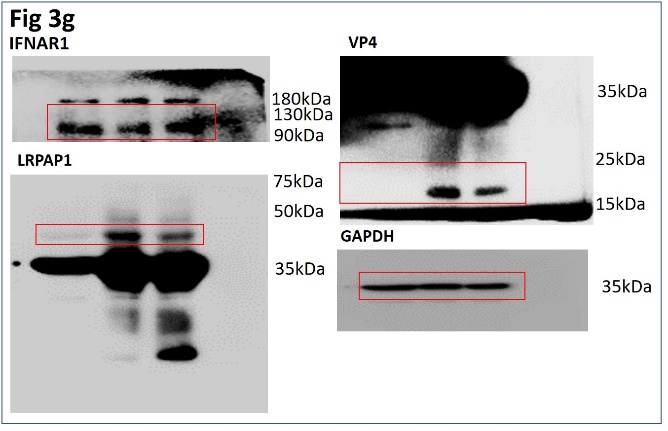

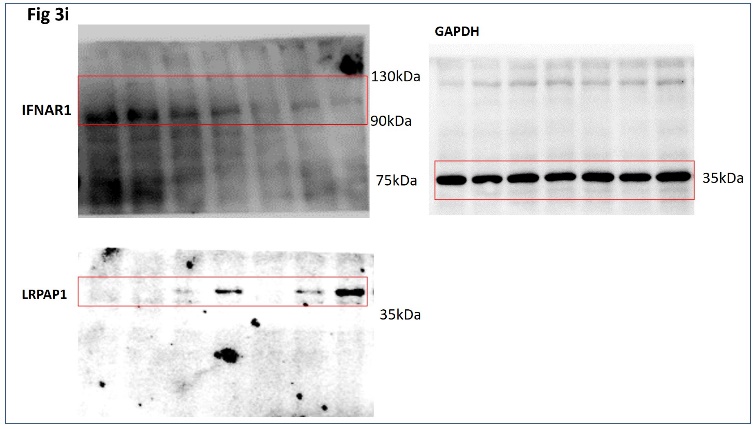


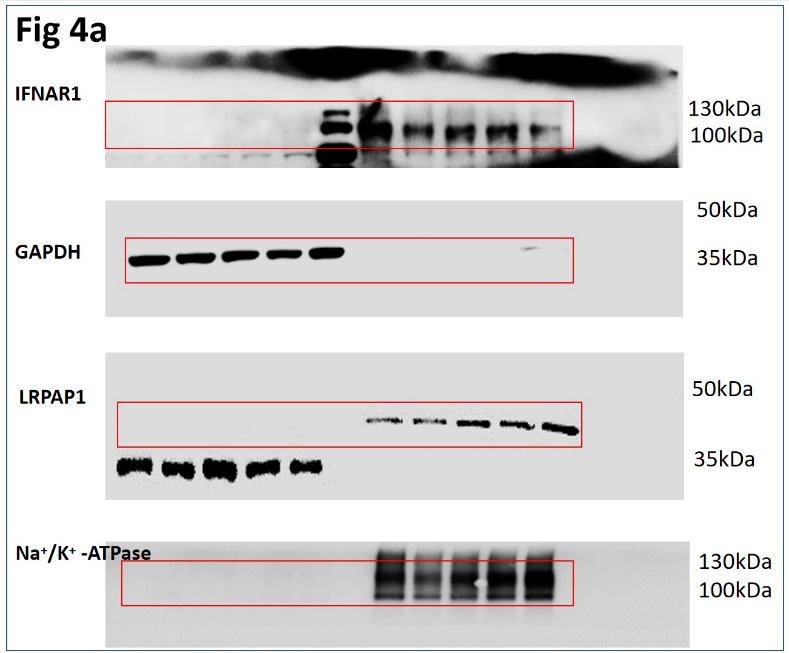

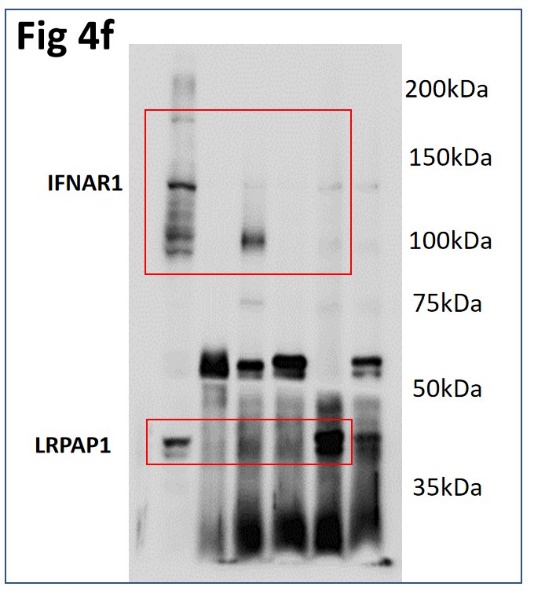


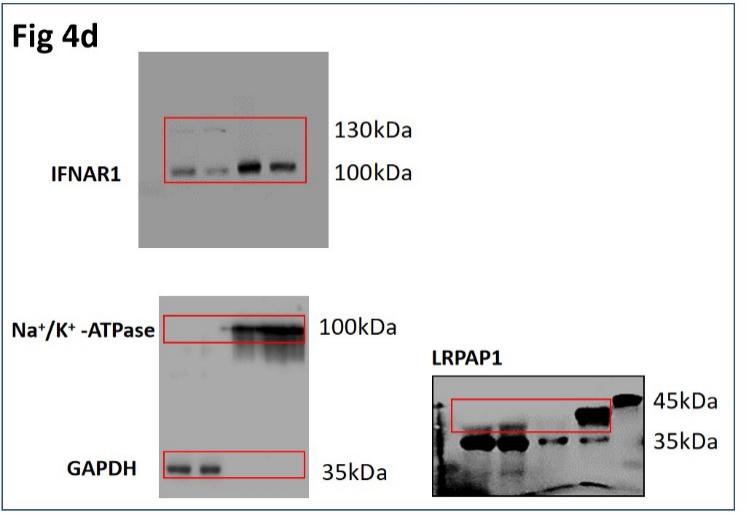

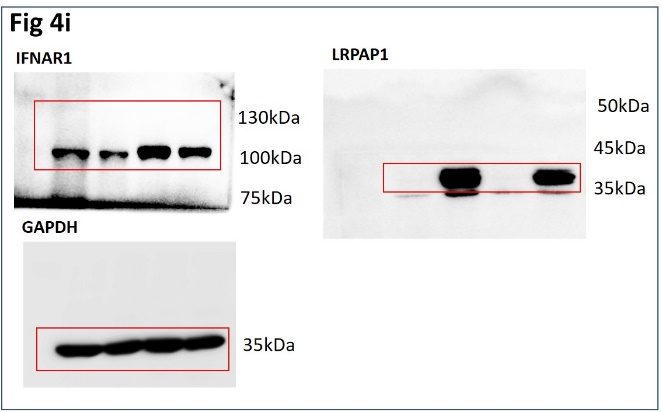

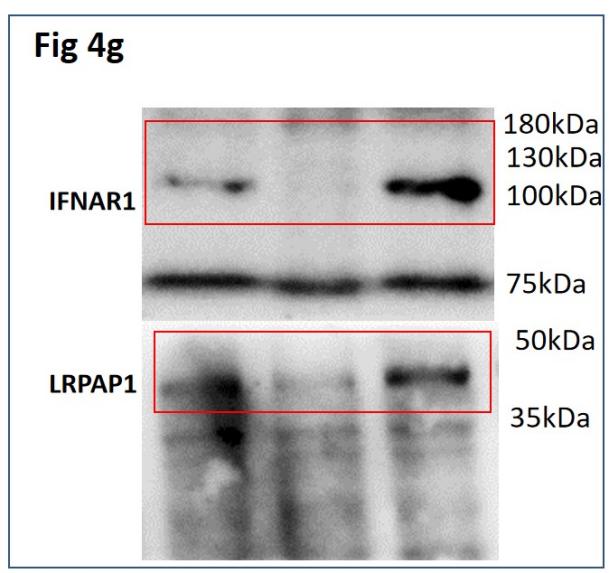

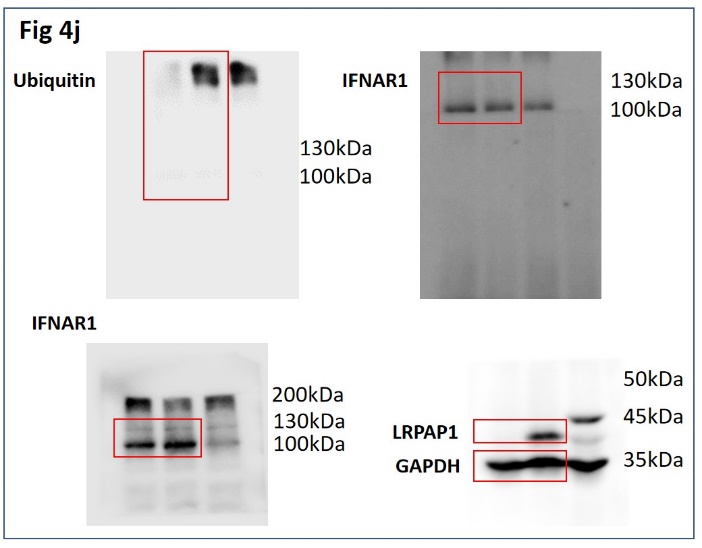


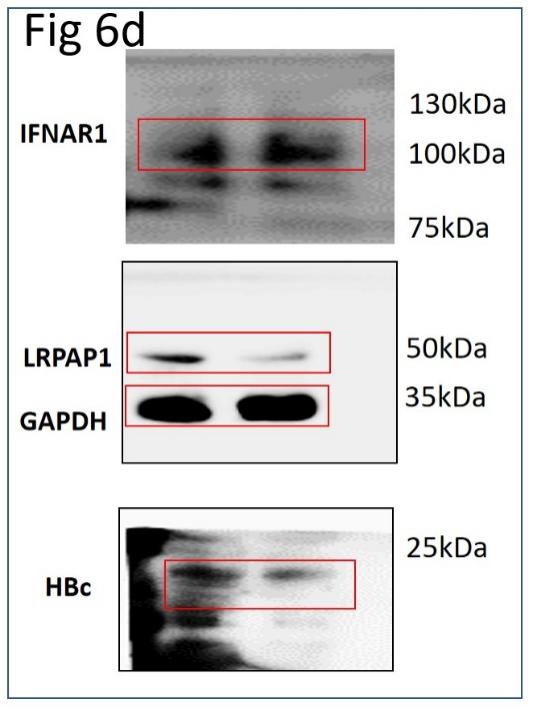

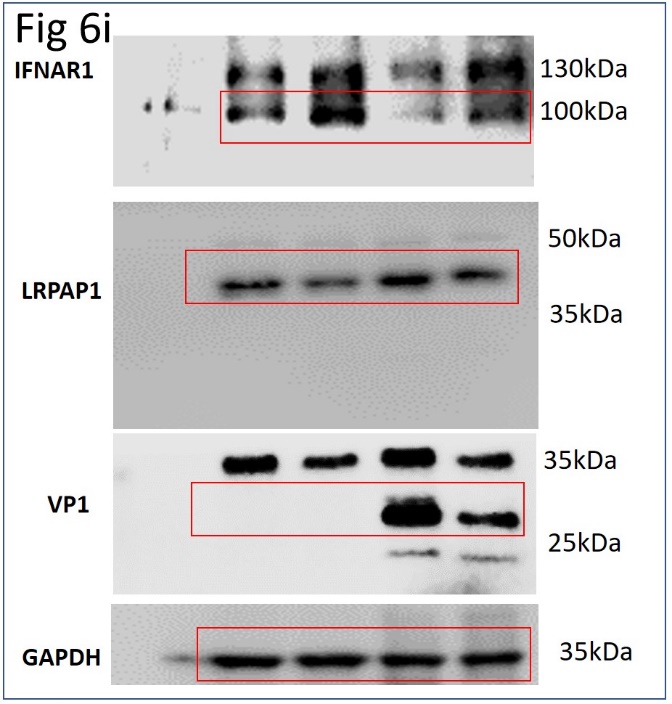


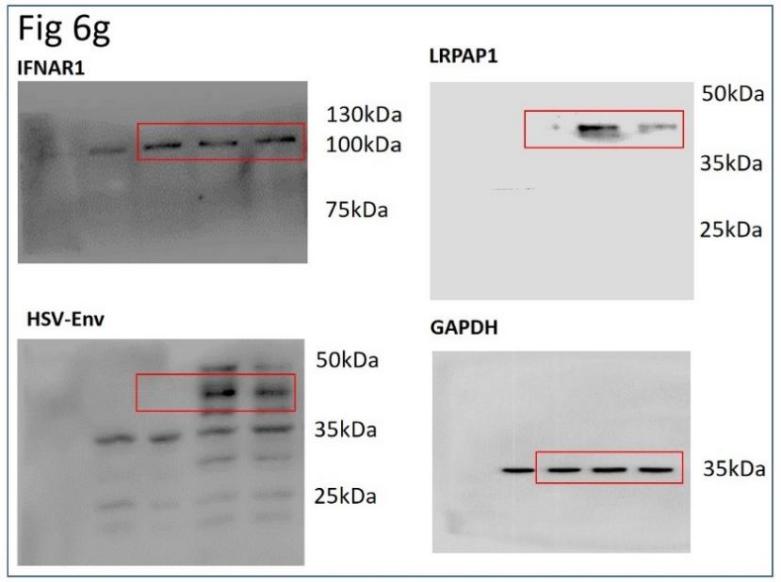

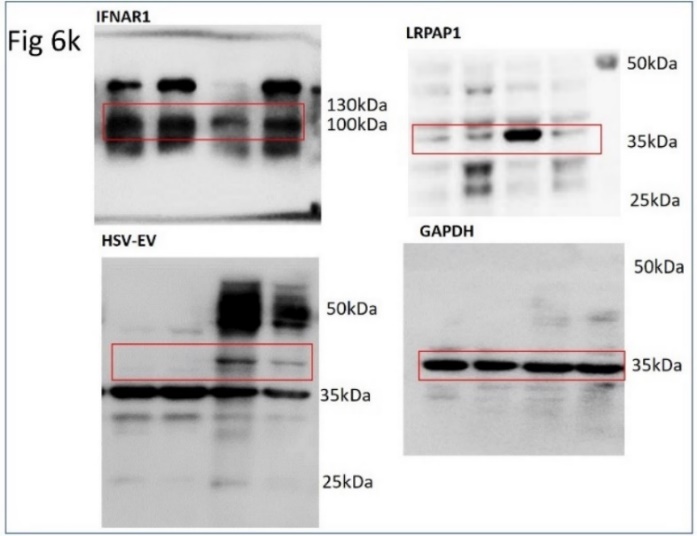


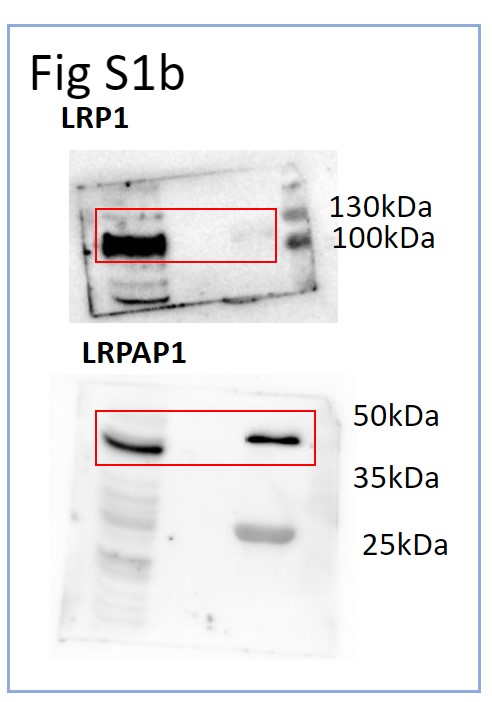

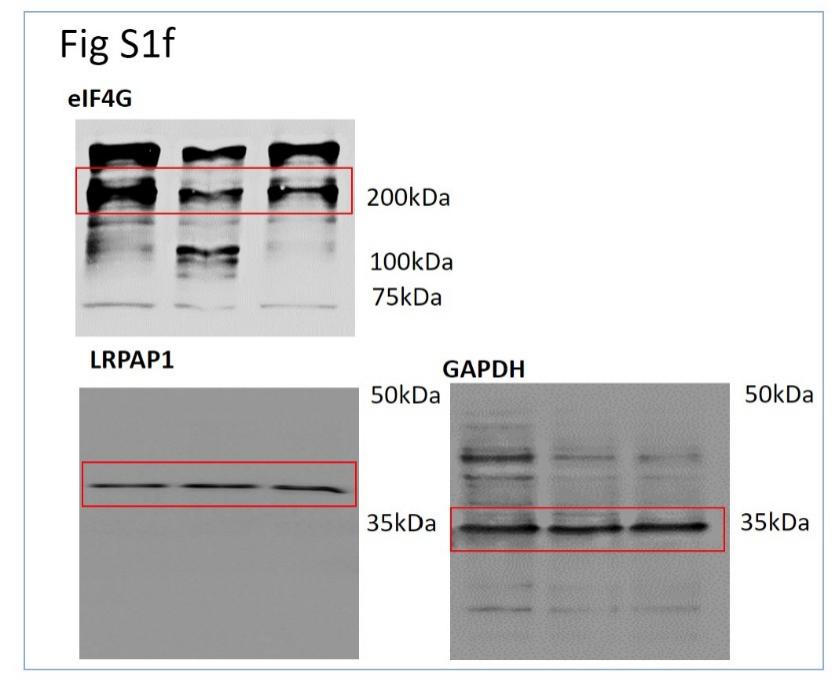

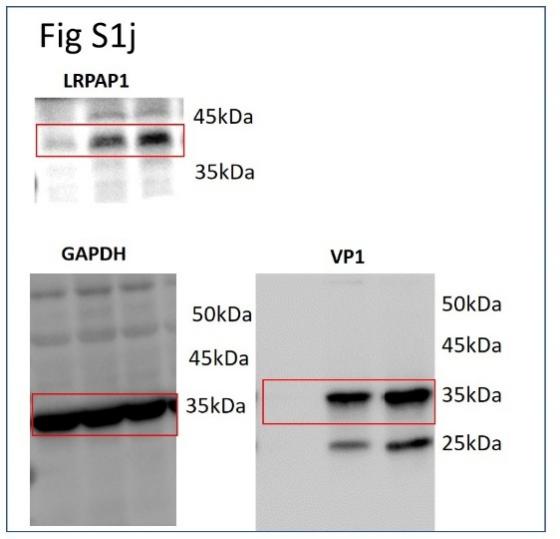

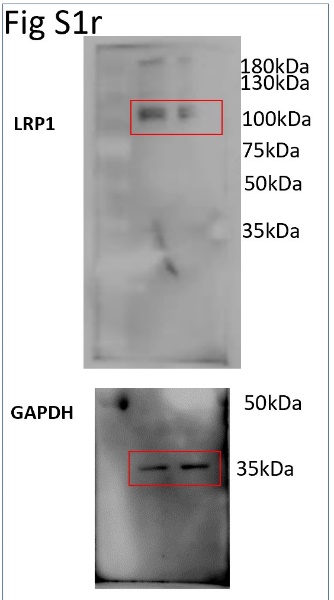


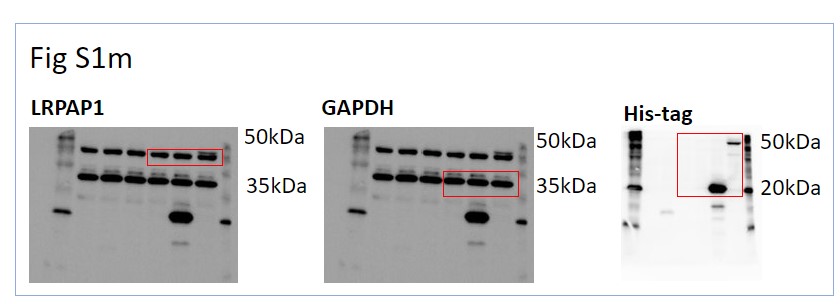

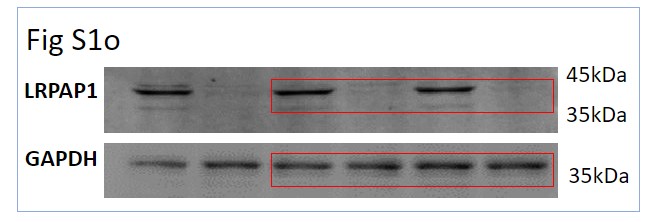


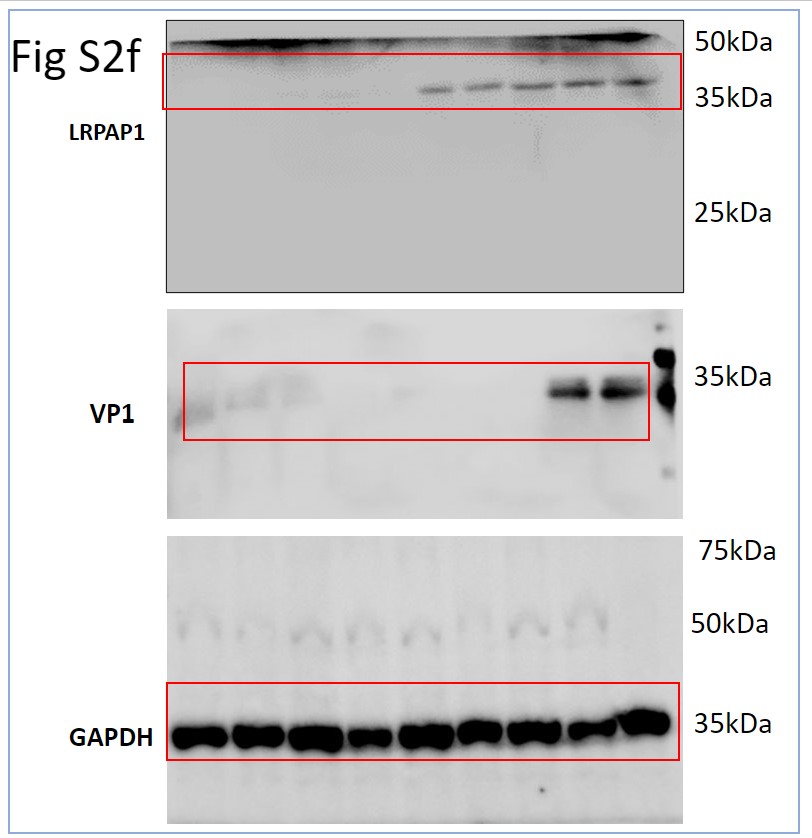


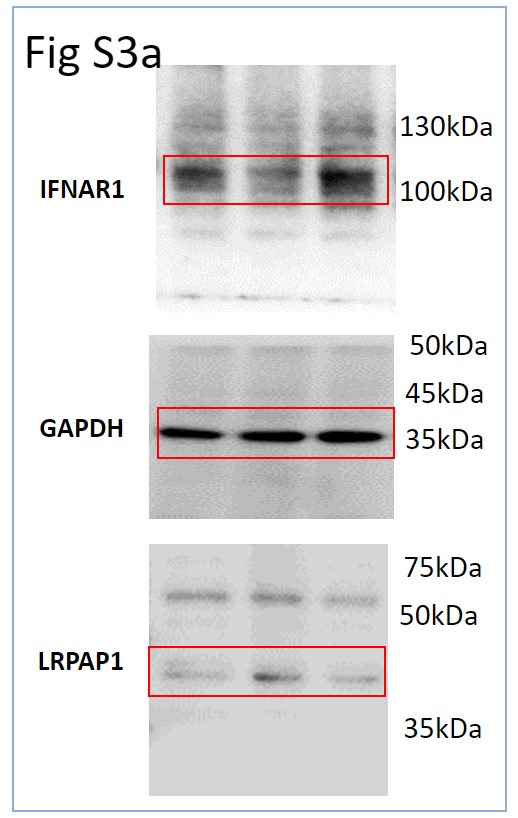

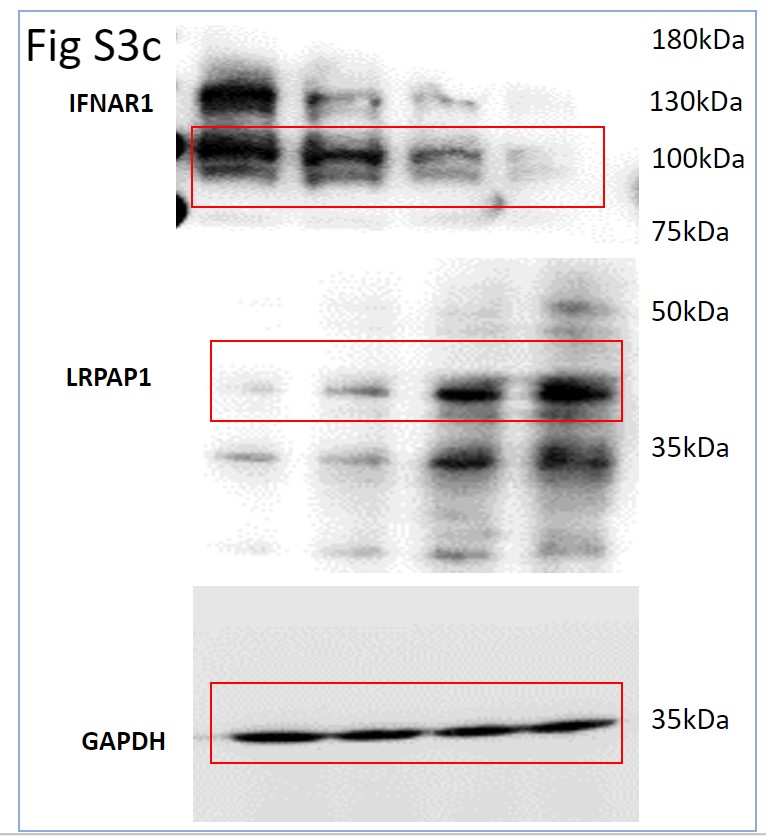

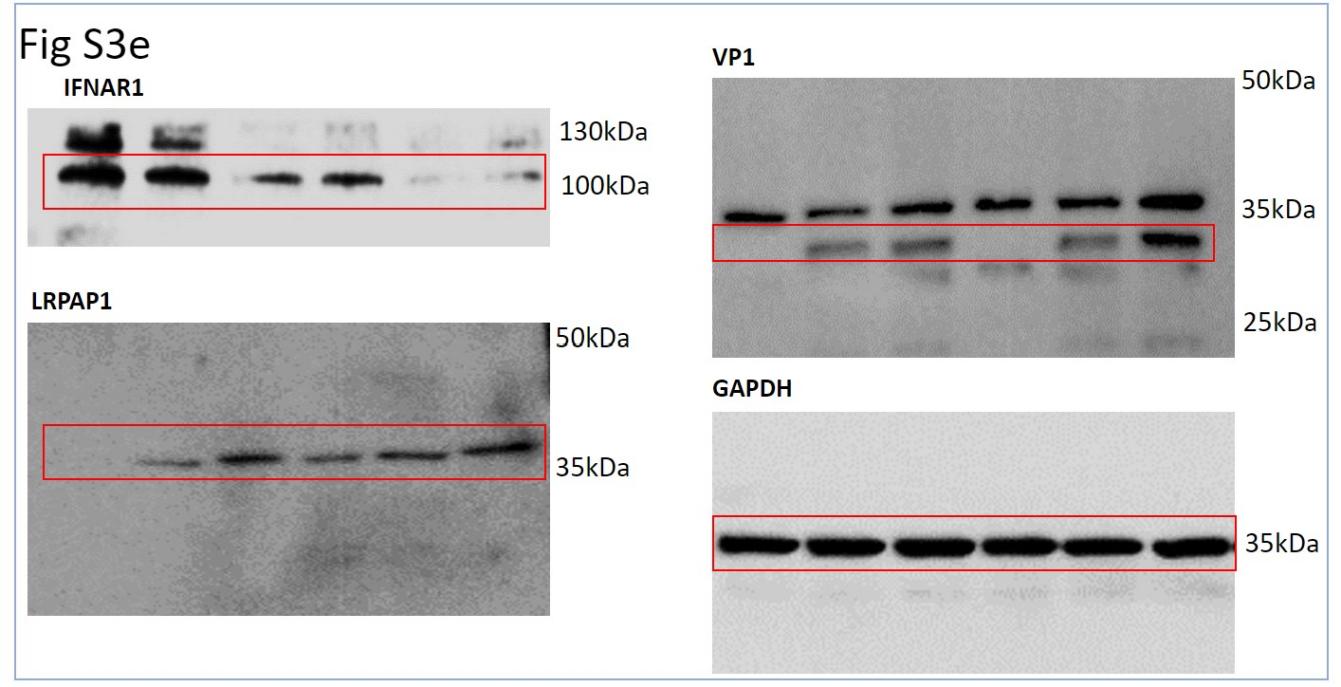


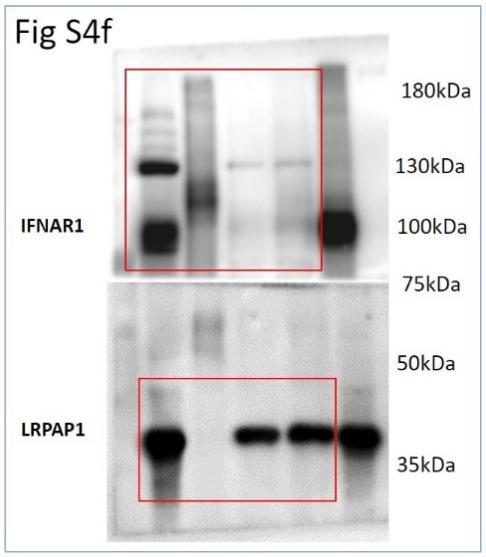

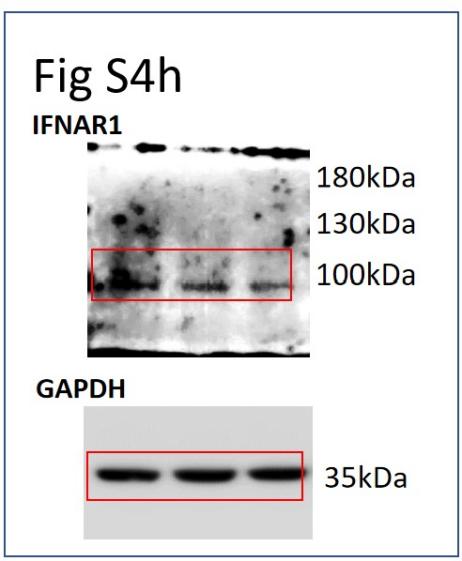


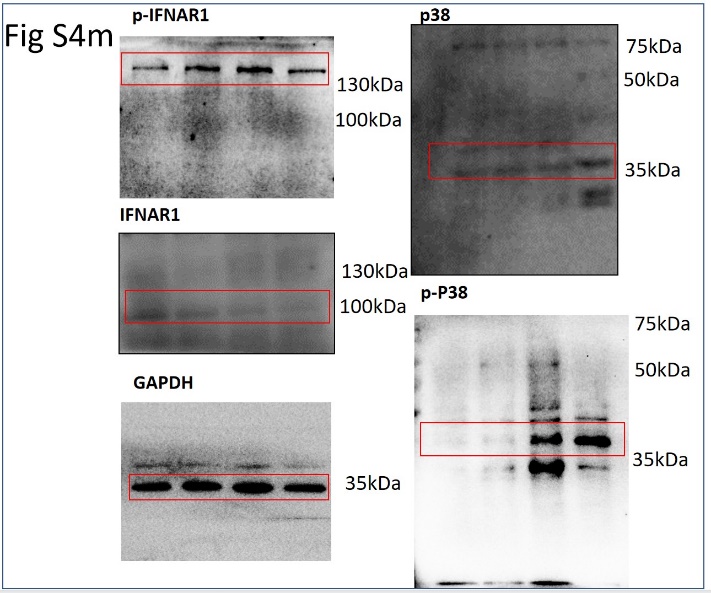

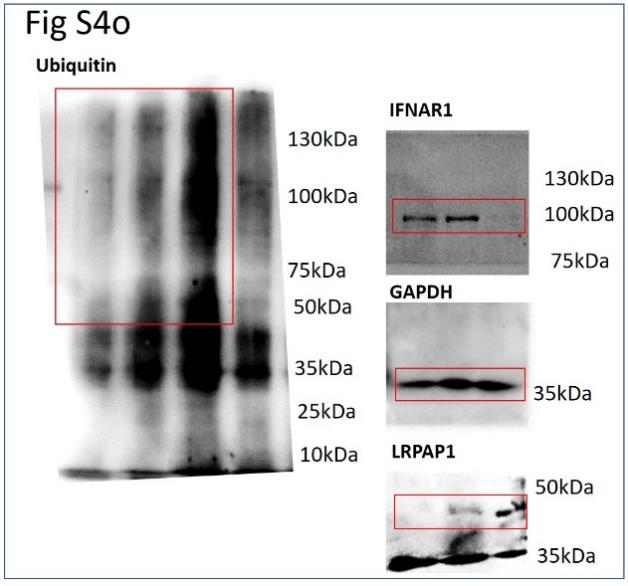

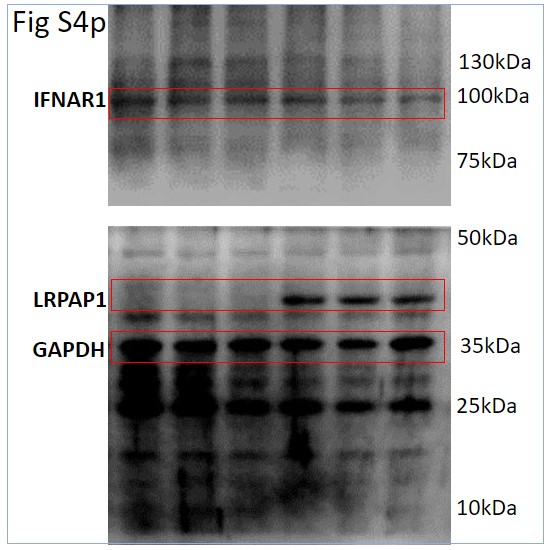

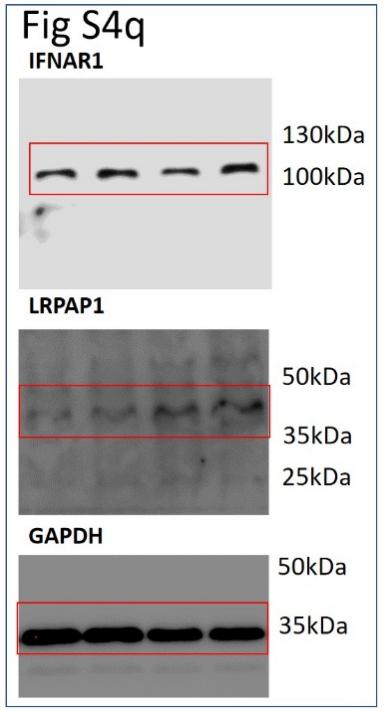


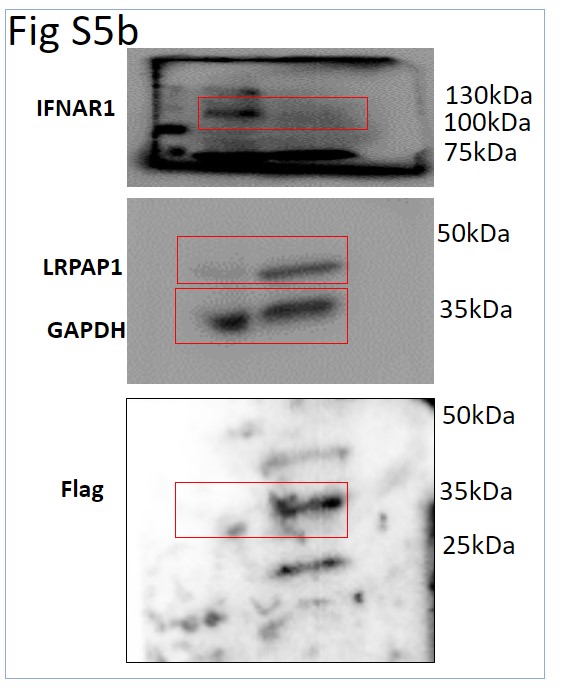


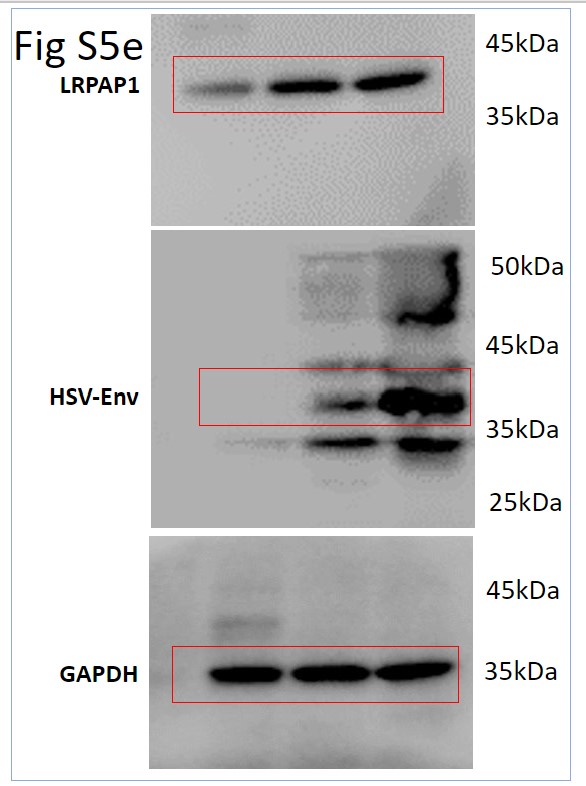


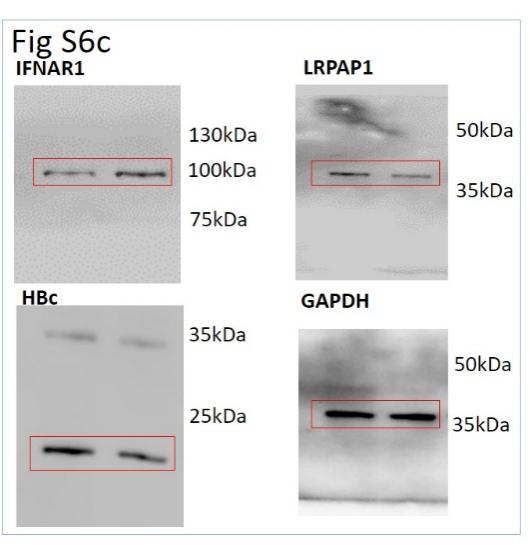

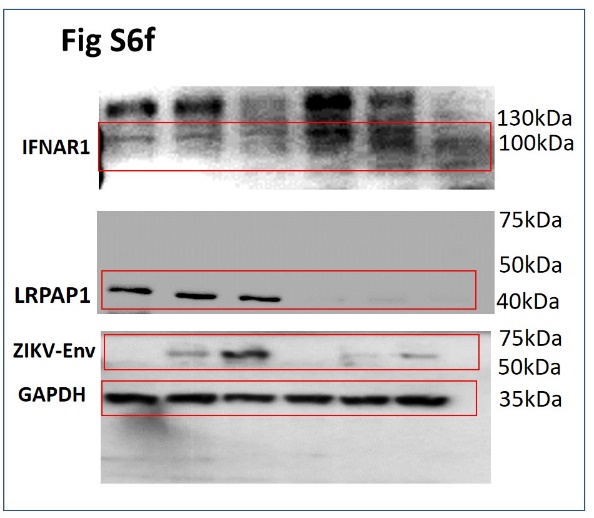

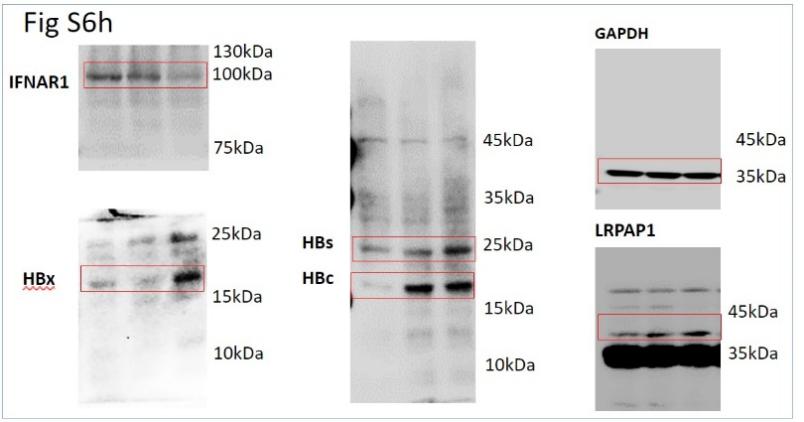

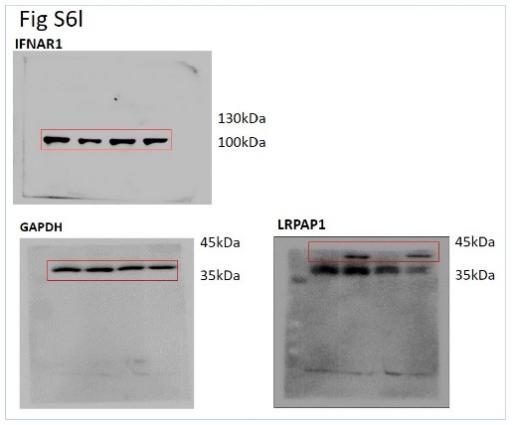

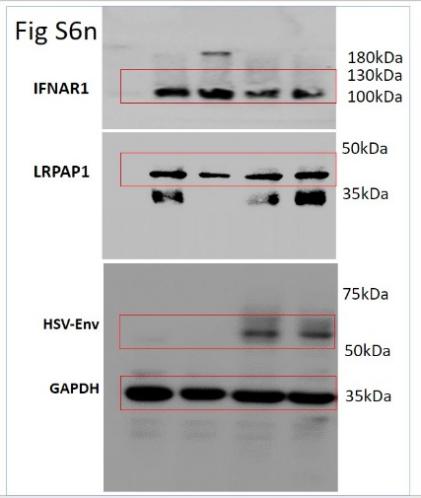

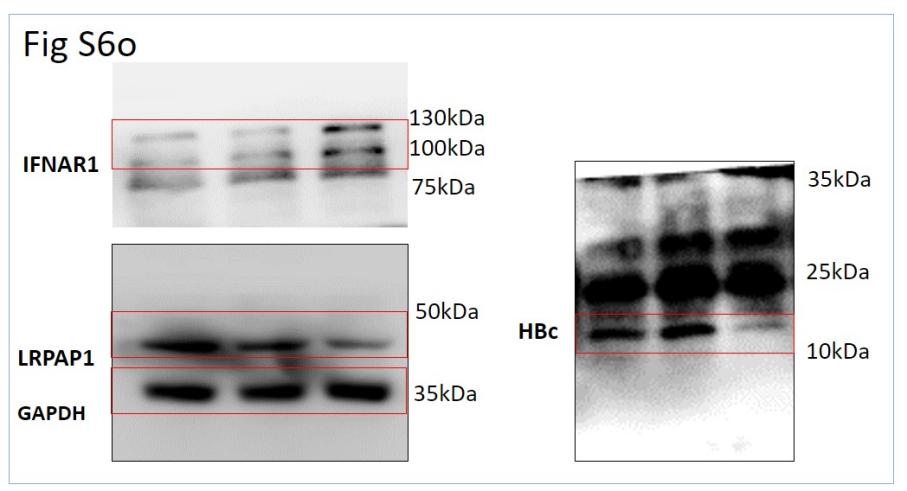


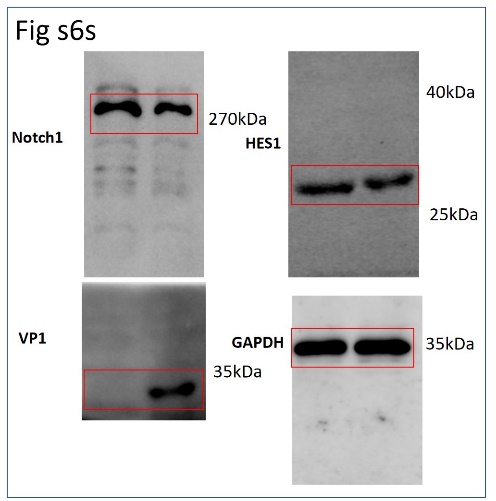

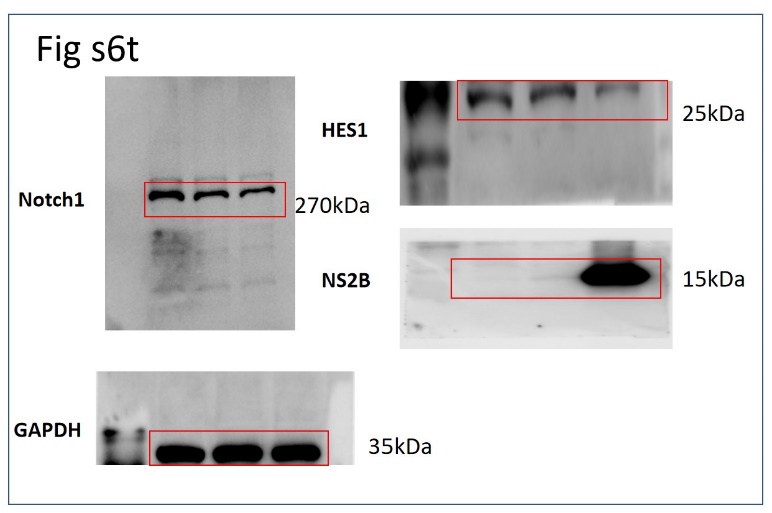

Supplement: Supplementary file 5 — Supplementary Materials [file 41392_2023_1630_MOESM5_ESM.docx]
